# Light-driven micro/nanobots

**DOI:** 10.1515/nanoph-2025-0152

**Published:** 2025-12-17

**Authors:** Rigvendra Kumar Vardhan, Manish Kumar, Jolly Xavier

**Affiliations:** MiD-Q-MiNT Lab, SeNSE, Indian Institute of Technology Dehi, Hauz Khas, New Delhi 110016, India

**Keywords:** micro/nanorobots, robotic control, light-driven machines, micro/nanomachines, optoelectronics, micro electro mechanical devices

## Abstract

Modern technological evolution witnesses a fast-paced progress in the design, science, and technology of light-driven micro/nanomachines in the recent past. These micromachines have found enormous applications as micro/nanoscale manipulators, micromachined space exploration components, nano-sized cell positioning and control, and micro/nanorobots for drug delivery to name a few. This is not only due to their smaller size but also due to an ever-demanding necessity of micro/nanoscale functionalities with touch-free optimum control incorporating features such as propulsion, self-powered and controlled activation, energy efficiency, intelligence, navigation, and tracking. It also motivates one for biomimicking the functionalities of several living organisms to mold the ideas into micro/nanorobots to understand their properties and the underlying physics. Incorporating the magical functionalities enabled by nano/micro photonics answer many a challenge while they also open a wide range of possibilities ahead. Here, we present light-driven micro/nanorobots (µn-Bots) whose robotic features and functionalities are envisaged to have potential applications in medicine, industry, rescue, and strategic deterrence, pertaining to all walks of life and spectrums. After giving a comparative as well as the state of art outline on advances on the diverse technological innovations of µn-Bots in general, we comprehensively go through the light-driven micro/nanorobot designs and explore their functionalities, materials, and micro/nanofabrication techniques concerning their recent advances and multifaceted applications. On the other hand, we also give an analysis on the performance matrix of the reported light-driven micro/nanorobots explicitly studied in the recent past and give an outlook on the future roadmap and trends.

## 1 Introduction

**Figure 1:**
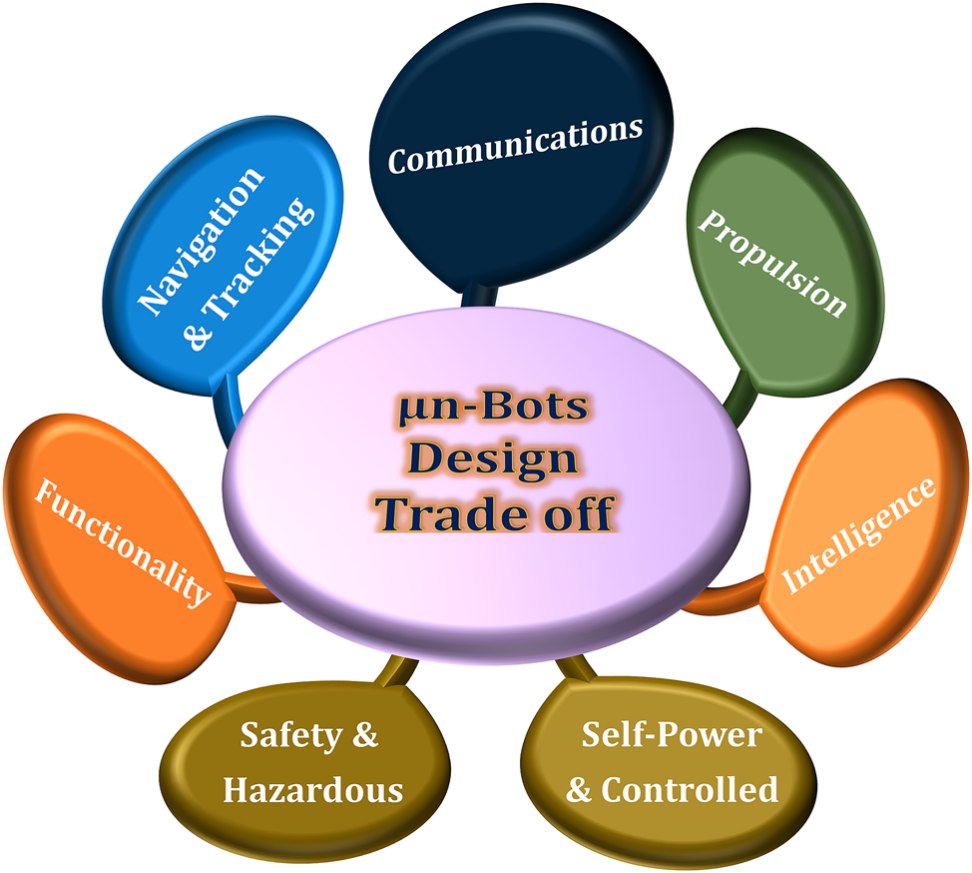
Micro/nanobots (µn-Bots) design trade off.

Miniaturization and automation are ever evolving technological innovation in all fields of engineering and science. So, it is true also in the field of robotic control and structural modules because of fast paced progress in micro/nanofabrication and enabling robotic technologies. Diverse types of micro/nanobots (µn-Bots) have been reported in the recent past for their distinct propulsion and ability to perform various functions in response to an external stimulus for carrying out the repetitive or hazardous tasks of our daily lives. Significant efforts in the development of μn-Bots have been made with diverse materials, control methods, and manufacturing techniques in order to evolve and optimize appropriate micro/nanoscale functionalities by incorporating features such as propulsion, self-powered and controlled activation, energy efficiency, intelligence, navigation, and tracking reported in the literature to be considered as µn-Bots design trade-offs being shown in Figure 1.

Nature-inspired innovations have been pursued to build µn-Bots. For example, inspiration from the natural movement of a biological microorganism caused by the hydrolysis of bioenergy, such as the swim propulsion of sperm and bacteria induced by the deformation of their bodies into an oar-like or screw-like shape, respectively, could lead to the motivation for the development of µn-Bots driven by chemical reactions [1], [2], [3], [4], [5]. Although any µn-Bot, whether human-controlled or self-driven, requires a power source to move at the either microscopic or nanoscopic scale, which further involve a design trade-off concerning their size and weight. However, such design trade-off between the power, size, and weight become severe in µn-Bots that can address with the utilization of soft materials or smart material like liquid crystal elastomer (LCE) [6], [7], [8], [9], [10], [11], [12], [13], [14], [15], shape memory alloy (SMA) [10], liquid crystal polymer (LCP) [9], [16], [17], [18], [19], [20], hydrogel [21], [22], [23], [24], [25], [26], [27], liquid metal [28], [29], [30], [31], [32], [33], [34], [35], [36], [37], [38], and many other. The µn-Bots with soft materials can be also driven by verities of external stimuli, including magnetic fields [39], [40], electric fields [41], [42], [43], [44], [45], [46], temperature gradients [47], [48], [49], [50], [51], chemical reactions [52], [53], [54], [55], acoustic fields [56], [57], [58], [59], [60], and light fields [4], [5], [61], [62], [63], [64], [65], [66], [67], [68], which is primarily background for their classification. Light, a potent and renewable source energy, plays a pivotal role in the very existence of life on earth [61], [69]. Nowadays, the potential of light will be seen in diverse areas, including integrated photonics circuits, micro/nanofabrication, material processing, information processing, quantum computing, single-molecule detection, and many more [62], [63]. So, to actuate and regulate the micro and nano-scaled structures with the light is not surprising. In this back drop, the technology has witnessed an evolution of light-driven micro/nanomachines in the recent past due to their enormous applications in micro/nanoscale manipulators, micromachined space exploration components, nano-sized cell positioning and control, micro/nanorobots for drug delivery, etc. Such µn-Bots not only have an attribute of their size but are also attributed to an escalating need for micro/nanoscale functionalities with touch-free optimum control, including propulsion, self-powered and controlled activation, energy efficiency, intelligence, navigation, and tracking [64], [65].

The general driving/actuation mechanism of the soft µn-Bots is classified here in the perspective of optical light functionality rather than the convention classification used previously into the nonoptical and optical actuation mechanisms discussed in detail at Section 2. However, optically triggered µn-Bots also have another classification based on their propulsion mechanism, which includes optical tweezers [66], [67], photochemical [68], photothermal [70], photomechanical [71], [72], and many other propulsion mechanisms. We present the design tradeoff, fabrication techniques, materials, and applications of optically triggered µn-Bots over other actuation methods.

The paper consists mainly of seven different sections, where the first section gives an introduction to the motivation for its latest research trends in general. The second section is more about the various actuation mechanisms of µn-Bots and why light actuation is gaining popularity in their design. Section 3 mainly discusses the numerous types of soft materials employed in designing nonoptical triggered µn-Bots prototypes for specific applications. Several optically induced techniques for the realization of µn-Bots are described in Section 4. Section 5 is mainly about the optically driven µn-Bots prototypes, which attract the research community and industry to quite a few interesting applications, including terrestrial micro/nanorobots, grabbing and transporting objects, environmental restoration and surface cleaning, bionic technology, biomedical applications, and biocompatibility insight in soft bio µn-Bots. Section 6 explores the potential and challenges of a multimodal collaborative control strategy combining optical actuation with another actuation mechanism. Finally, the challenges and advantages are summarized in Section 7 as a conclusion. So, the advances in optically driven µn-Bots aim to fascinate and motivate through their innovative design, functionalization, fabrication, and application in a broad horizon.

## 2 General actuation mechanism in soft µn-Bots

**Figure 2:**
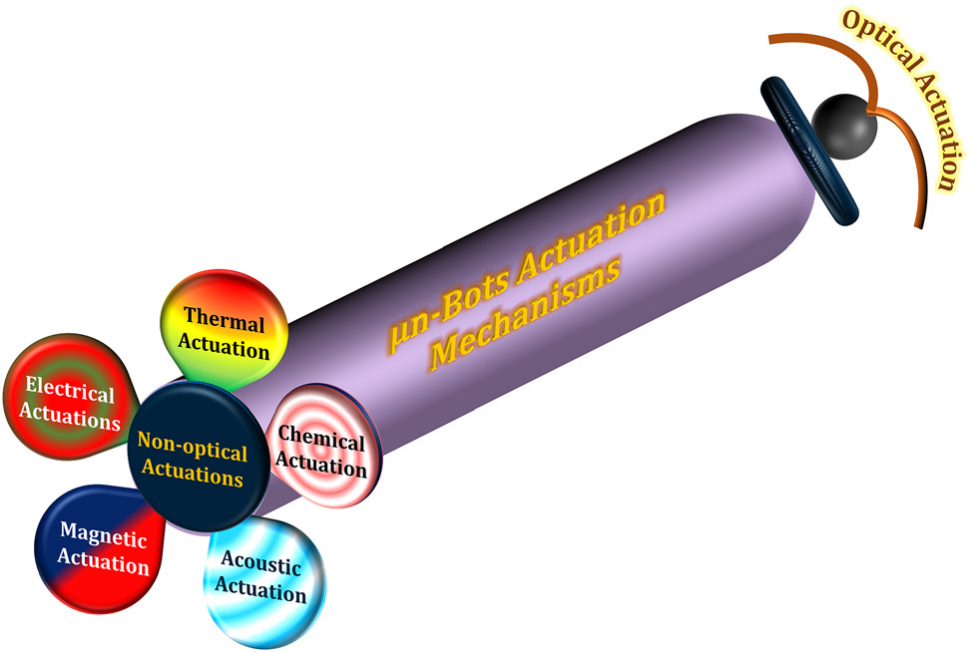
Actuation mechanism of soft µn-Bots.

**Figure 3:**
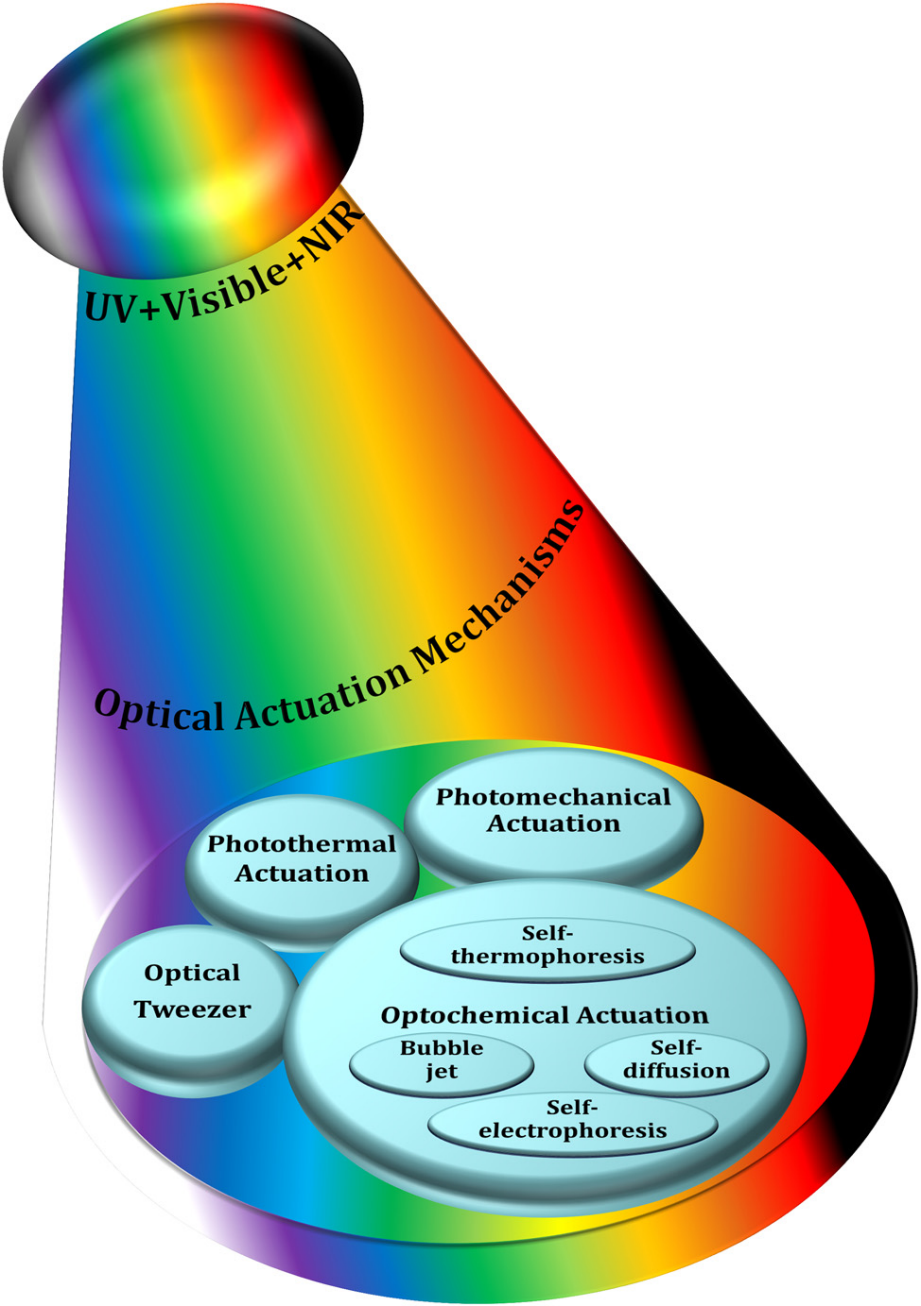
Classification of optically actuated soft µn-Bots.

Unlike their macro counter parts, micro/nanorobots rely heavily on their activation/driving mechanisms. The functional material body of the micro/nanomachine and its excitation signal plays a crucial role in the whole functioning and localized control of the device. So, for the ease of understanding, from the perspective of the present study on light-driven micro/nanorobots, the soft µn-Bots are categorized in general based on their activation/driving mechanisms whether they rely on nonoptical methods or that of optical actuation mechanisms illustrated in Figure 2. We have also tabulated the same in Table 1 for a quick comprehension. The nonoptical actuation mechanism mainly covers actuation due to stimuli other than light, including magnetic, electric, thermal, acoustic, and chemical. However, the optical actuation mechanism is preliminarily driven by the light–matter interaction that triggers one of the following: the chemical reaction, mechanical force, deformation, etc., and accordingly it could further subcategorized as shown in Figure 3.

**Table 1:** General actuation mechanism of µn-Bots.

Actuation mechanism	Material type		Applications	Ref.
Magnetic actuation	Magnetoactive elastomer polymer		Soft microrobot, microgripper, origami folding	[73], [74], [75]
	Ferromagnetic		Ferromagnetic soft catheter robot	[73], [76]
	Paramagnetic (NdFeB)		Millimeter untethered swimmers, microwire steering, and origami fish steering	[77]
	Magnetic microparticles		Microrobot, microgripper, helical microrobot	[74]
	Magnetized nanocomposite hydrogel		Actuators	[76]
	Magnetic liquid metal		Biomimetic soft robots, reconfigurable actuators	[31]
Electrical actuation	Silicon		Solar powered microrobot, energy harvesting, sensor and actuators	–
	Metal		Drug delivery, sensor, and actuators for robotics.	–
	Piezoelectric		Sensor, actuator, energy harvesting and robot propulsion	[44]
	Conductive polymer		Actuator, soft artificial muscles	[42], [43]
	Dielectric elastomer		Actuator, soft artificial muscles, and microrobots	[78], [79], [80], [81]
	Liquid metal		Soft robotics, micromotors	[32], [33], [34], [35], [36], [37], [38]
Thermal actuation	SMA		Soft robotics and actuators	[10], [82]
	Bimetallic strips		Multi DOF robot	–
	Metals		Thermal actuator for robotic propulsion	–
	Polymer		Soft robotics and actuators	[48], [49], [50], [51]
Chemical actuation	Polymer		Soft material, complex shape molding, microfluidic channel, and biocompatible shape modeling	–
	Hydrogel		Drug delivery release and control	[83]
	Shape memory polymer		Sensor application	–
	Metal		Catalyst for chemical reactions	[82], [83], [84]
	Liquid metal		Self-propelled micro/nanorobot	[29]
Acoustic actuation	Piezoelectric		Acoustic actuator and sensor	[85], [86], [87]
	Polymer		Complex shape molding, drug delivery, and biocompatible shape modeling	[85], [86], [87]
	Fluid		Propulsion medium	[58], [59], [60], [85], [86], [87]
Optical actuation	Photo sensing material		Self-diffusion propulsion	[88], [89], [90]
	Organic compound material		Surface topography, cell capturing, and microprobe	[91], [92]
	Photocatalytic material		Self-electrophoretic propulsion	[93], [94]
	Photothermal material		Photoacoustic tomography, cargo transport, water treatment, and swarming of micro/nanorobot	[7], [8], [95], [96], [97], [98], [99]
	Photo deformable material	Liquid crystal polymer	Programmable actuators and crawler	[9], [16], [17], [18], [19], [20]
		Hydrogel	Thermal responsive actuator, light-driven actuators	[21], [22], [23], [24], [25], [26], [27]
	Liquid metal		Opto-thermophoretic propulsion, opto-electrophoretic propulsion	[28], [30], [100]

### 2.1 Magnetic field–based excitation

The magnetic field–driven soft µn-Bots achieve propulsion by converting magnetic energy into mechanical energy, which is very prevalent, simple to make, and easy to use [39], [40]. Typically, magnetic material is incorporated on the surface of the soft µn-Bots or embedded inside the soft µn-Bots body to interact with externally controlled magnetic fields. However, the required magnetic field strength may change depending on the environment in which the soft µn-Bots need to move. For example, a soft body such as hydrogel can behave as a microrobot by incorporating ferromagnetic material evenly distributed on the actuating part or suspended in its body, whose orientation and strength are adjusted at the time of microfabrication using a permanent magnet or an electromagnet [76]. The propulsion of such a soft µn-Bots is controlled by an external magnetic field induced by a magnetic field generator, either an electromagnet or a permanent magnet. Precise movement of the soft µn-Bots is achieved by controlling the magnetic field strength and orientation without physical contact. This is a significant advantage where physical contact is susceptible to damaging the µn-Bots or its surrounding environment. It offers exceptional potential in medical applications because its penetration depth inside the nonpenetrating tissue is excellent, and it is commonly used for drug delivery [101], target therapy [102], [103], cargo transport [40], [104], and environmental remediation [105]. However, several challenges, such as magnetization, film coating, and fabrication methods, are encountered in the development of magnetically driven soft µn-Bots. Furthermore, magnetic actuation is ideal for driving soft µn-Bots with sizes ranging from tens to hundreds of microns. However, significant limitations in driving microrobots with sizes of several microns, particularly in magnetic actuation, necessitate more sophisticated auxiliary drive and control equipment, which in turn necessitate a complex hardware system.

### 2.2 Electrical actuation mechanism

Electrically powered µn-Bots are predominantly characterized by the properties that include charge, conductivity, and a semi-conductive nature that interacts with the activating electric field [41], [42], [43], [44], [45], [46]. The orientation, position, and speed of such µn-Bots are efficiently manipulated with the control of the applied electric field [42], [43], [44]. The electric field gradient in µn-Bots is harvested with numerous methods, such as applying the required voltage to an electrode, piezoelectric actuator, or using a microfabricated structure [42], [43], [44]. However, µn-Bots can be remotely controlled by electrorotation, electro-percolation, and electrophoretic motion, which are the foundations of wireless control driven by AC and DC electric fields [45], [46]. However, it needs a special buffer and has a significant compatibility issue with bio samples and highly ionic media. In addition, such µn-Bots movement is limited because the electric field’s strength decays rapidly with increasing distance. Microgripper, microsurgery [44], microassembly [42], and microfluidics [45], [46] are a few examples of the electrical control of µn-Bots utilized in manipulating biological cells or assembling microscale components in a controlled way.

### 2.3 Thermally driven µn-Bots

Thermal actuation is another method that uses heat to regulate the motion of µn-Bots. Thermally actuated µn-Bots, also known as thermos-bots or micro thermomechanical systems (MTMS), are tiny machines that use heat energy to operate. Typically, it is constructed from materials having both thermal and mechanical properties. Thermally sensitive materials like shape memory polymers or metals are utilized that involve heating or cooling a particular regime of the µn-Bots, causing it to expand or compress to produce motion. The benefit of thermal actuation in the µn-Bots is the fast response time that can be achieved by readily applying or withdrawing heat using various methods, such as joule heating, infrared radiation, or laser heating. It can provide a straightforward and effective way of controlling motion. Furthermore, complicated motion patterns can be produced using thermal actuation by meticulously regulating the temperature distribution across the microrobot. Microgripper, drug delivery, and microsurgery are just a few of the thermally actuated µn-Bots or thermo-bots. For instance, a thermos-bot can be used to assemble microscale parts, transport medications to specific areas of the body, or carry out minute surgical procedures [47], [48], [49], [50], [51].

### 2.4 Chemical actuation of µn-Bots

The chemical reaction is especially advantageous for µn-Bots propulsion intended to operate in fluid environments, such as the human body, over other actuation techniques because it enables motion generation without external forces or power sources. Such µn-Bots movements and motion are controlled through chemical processes induced by chemical reactions to produce gas or a change in solubility. It can offer a reasonably straightforward and affordable way of controlling motion, which is a key benefit, and can be used to accomplish particular motion patterns and reactions, such as reacting to pH or temperature changes. The microtubular jet microrobots [52], catalytic micro/nanomotors [53], and electro-osmotic microswimmers [54], [55] are a few examples of chemically actuated µn-Bots whose motion is mainly regulated by the involved reaction rates. Diverse chemically actuated µn-Bots are utilized in applications such as medication administration, environmental tracking, and microfluidics. It can be widely used to regulate fluid movement in microchannels, transport medications to particular body parts, and observe the environment in real time [52], [53], [54], [55].

### 2.5 Acoustic actuation mechanism

Acoustic field-propelled µn-Bots, also known as acousto-bots or micro-acousto-fluidic systems, have great compatibility with biological environments because they require on-demand and noninvasive motions. Such µn-Bots are driven by employing the acoustic field distribution, which can be altered through amplitude, frequency, and phase using the interference principle. In acoustic actuation, sound waves produce pressures that work on the structure of the µn-Bots, propelling them in one direction or allowing them to carry out a particular job. Different methods, such as acoustic streaming, radiation pressure, or acoustic levitation, can be used to accomplish acoustic actuation. Acoustic streaming happens when sound waves cause vortices to form in a fluid setting. These vortices then combine to form a net fluid flow, which powers the µn-Bots. The force that sound waves apply to the surface of the µn-Bots, known as radiation pressure, propels them in the direction of the auditory source. So, such µn-Bots can be captured and operated in a precise way using acoustic propulsion. Microbead rotation and metal nanowire propulsion are a few examples of µn-Bots driven by the acoustic field [56], [57], [106], but they are limited in many applications because of their low resolution. One benefit of acoustic actuation is that it can be used to concurrently operate many µn-Bots, which is challenging to accomplish with other actuation techniques. Acoustic actuation is a potential method for µn-Bots use in medicine, such as medication administration or targeted treatment, because acoustic waves can also enter deeply into tissues [58], [59], [60], [107].

### 2.6 Optical actuation and manipulation of µn-Bots

Light–matter interaction is the key in the optical actuation of µn-Bots that further depends on the operational wavelength regimes of light such as UV, visible, and IR and photosensitive properties of the functional material used for the fabrication of µn-Bots.

Here, we classify the principles and mechanisms of light-driven µn-Bots into primarily the categories of rigid type and nonrigid type. The rigid type soft µn-Bots typically has no deformation during the operation and manipulation. Also, it can be mostly driven with an optical tweezer, optical trapping, momentum transfer method, photochemical reaction, etc. However, the nonrigid type soft µn-Bots tend to deform during any operation, even during propulsion. Such nonrigid type soft µn-Bots can be designed in a materialistic way that is further controlled by light actuation, including the photothermal effect and photomechanical effect. The detailed classification of optical actuation schemes being reported in the literature, including an optical tweezer [69], [91], [92], [108], [109], [110], [111], [112], [113], [114], [115], [116], [117], [118], [119], [120], [121], [122], [123], [124], [125], [126], [127], optochemical reaction [88], [89], [90], [93], [94], [128], [129], [130], [131], [132], [133], [134], [135], [136], [137], [138], [139], [140], photothermal [7], [8], [9], [16], [17], [18], [19], [20], [21], [22], [23], [24], [25], [26], [27], [95], [96], [97], [98], [99], [141], photomechanical [97], [142], [143], [144], [145], [146], [147], [148], etc., is depicted in Figure 3.

Among all the above driving categories of µn-Bots, optical tweezers are one of the first µn-Bots propulsion mechanisms that are commonly used to control and manipulate the movement of 3D-printed rigid polymeric µn-Bots. It utilizes forces generated by highly focused light with steep intensity gradients, allowing for precise and noninvasive manipulation and actuation of tiny objects suspended in either air or liquid. It can serve as a tool for single-cell capture or as detecting probes for the object in the surface topography. However, the µn-Bots driven by optochemical actuation mechanisms are made with photoactive material, which often includes structures like Janus spheres, hollow tubes, and rods. It functioned with optochemical reactions such as photochromic and photocatalytic effects, converting chemical energy into motion. Such µn-Bots have potential applications for water purification and drug delivery in environmental protection and biomedical industries. On the other hand, optomechanical mechanisms are primarily used to drive nonrigid type µn-Bots made of soft materials like liquid crystal polymers (LCP). Also, photothermal actuation mechanisms can be used to drive optomechanical soft microrobots, optochemical microrobots, and 3D-printed hard polymeric microrobots, so they can be used to power a variety of µn-Bots, such as ones that pick up and place objects, mimic flowers, and act as fly traps. Section 4 will outline the working mechanisms and foundational principles behind such light-driven µn-Bots and their strengths and limitations.

We have summarized in general in Table 1, though it may not be all-exhaustive, the materials in the design of soft µn-Bots and their actuation/driving mechanisms. However, several materials show both the characteristics that make used to achieve additional features in soft µn-Bots designs. The material, which response either of the magnetic, electrical, thermal, chemical, or acoustic field, considered as the nonoptical actuations. However, optically triggered µn-Bots are a fascinating and rapidly developing field of research that holds promise for a wide range of applications. Several types of optical materials are commonly used to develop light-driven soft µn-Bots, including photo-sensing, photocatalytic, photothermal, and photo-deformable materials. Based on the studies of various nonoptical and optical materials, Table 2 summarized the optimum materials that can be utilized for the design of µn-Bots in the optical and nonoptical actuations methods.

**Table 2:** Materials in µn-Bots actuation.

Materials	µn-Bots actuation mechanism					
	Optical	Nonoptical				
		Magnetic	Electrical	Thermal	Chemical	Acoustic
Metal thin film/nanoparticles	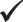		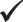	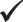	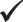	
Liquid metal	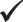	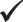	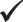		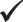	
SMA	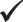		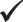	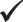		
Photoresists	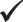			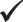		
Polymers	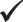			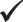		
Photo sensing materials	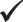					
Organic compound materials	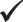					
Photocatalytic materials	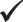				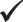	
Photothermal materials	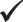					
Liquid crystal polymer	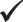					
Hydrogel	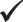	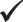				
Silicone/PDMS	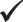	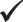	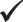	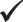		
Silicon			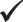			
Conductive polymers			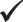			
Carbon nanotubes			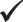			
Bimetallic strips				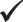		
Ceramics			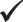			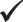
Piezoelectric materials			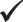			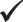
Microbeads						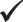
Fluids						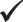
Magnetic microparticles		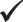				
Magnetoactive polymers		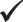				
Magnetized nanocomposite hydrogels		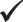				
Paramagnetic (NdFeB)		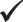				
Ferromagnetic		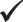				

## 3 Nonoptical actuation mechanism in soft µn-Bots: applications

Here, we outline in detail the state of art in the design of various soft µn-Bots by utilizing the nonoptical actuation methods includes magnetic, electric, thermal, chemical, and acoustic actuation. Also, it infers the advantages of soft material even in the other nonoptical actuation methods in the improvement of the power to weight trade-off, efficiency, size, and weights.

### 3.1 Magnetic field–driven soft µn-Bots

**Figure 4:**
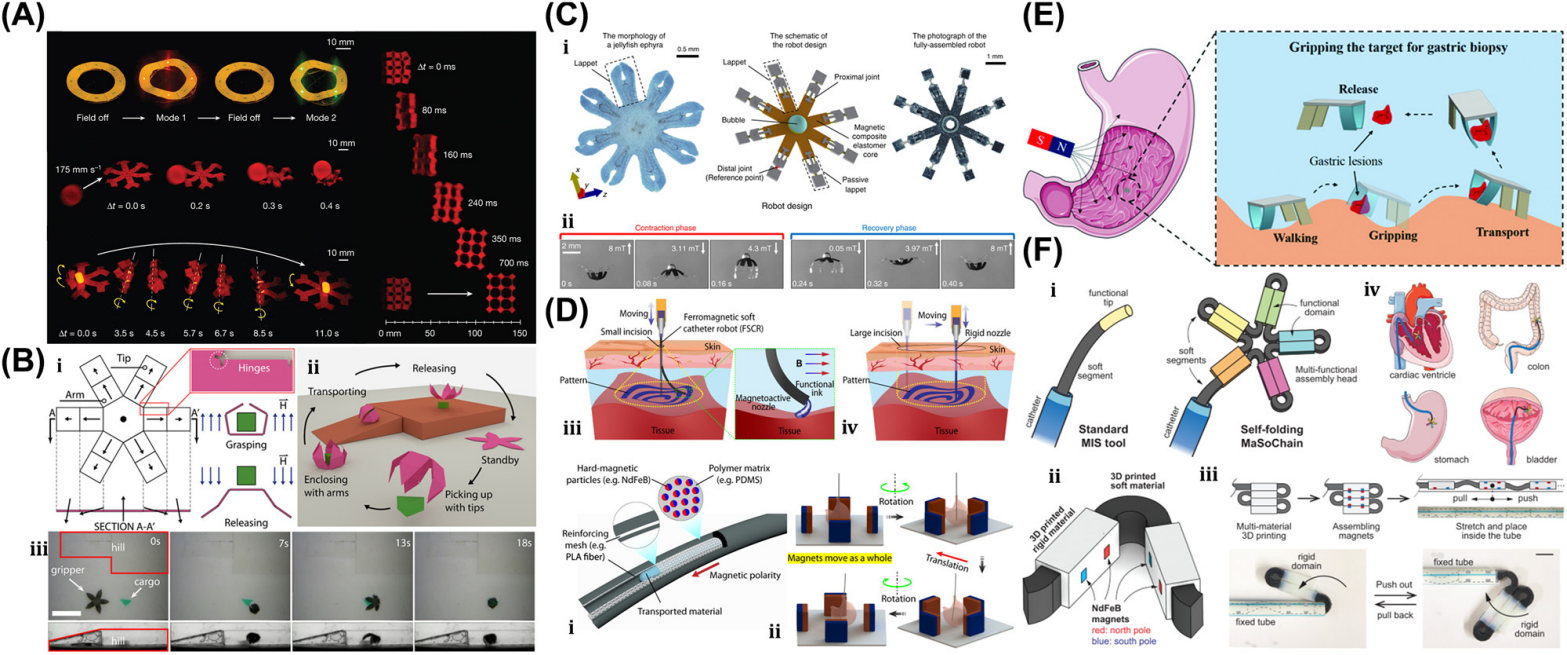
Magnetic field–driven soft bots. (A) Locomotion of hexapedal structure under a rotating magnetic field through a permanent magnet. Reproduced with copyright permission from [73]. (B) (i) Schematic, magnetization profile, and principle mechanism of a magnetic microgripper. (ii) Cargo transportation task. (iii) Cargo transportation in silicone oil environment. Reproduced with copyright permission from [74]. (C) (i) Schematic of jellyfish-inspired soft magnetic microrobot made of magnetic elastomer composites. (ii) Microrobot locomotion in the contraction and recovery phase. Reproduced with copyright permission from [75]. (D) (i) Bioprinted ferromagnetic soft catheter robot inside the human body through small incisions. (ii) Comparison of traditional printing systems with rigid nozzles and a soft polymer matrix with hard-magnetic particles and polylactide mesh. (iii) FSCR fabrication overview. (iv) Translational and rotational motion using four permanent magnets. Reproduced with copyright permission from [76]. (E) Conceptual model of magnetically actuated quadruped soft bionic microrobot locomotion includes walking, gripping, and transport in gastric biopsy. Reproduced with copyright permission from [149]. (F) (i) Self-folding catheter tool using MaSoChain. (ii) Fabrication and components of MaSoChain. (iii) Push out and pull back mechanism of the MaSoChain inside the sheathing tube. (iv) Demonstration of MaSoChain folding in the human body, such as heart ventricles, colon, stomach, and bladder. Reproduced with copyright permission from [77].

A unique method for manufacturing flexible, untethered, fast-transforming soft miniature robots by 3D printing ferromagnetic domains onto elastomers using Magnetic Field Assisted Projection Printing (M-FAPP) [73]. While printing, a magnetic field is applied to the dispensing nozzle to reorient the particle to achieve patterned magnetic polarity, which has better power density and actuation speed compared to existing active 3D-printed materials. This technique can be utilized for reconfigurable flexible soft electronics, enabling the soft robot to roll, crawl, transport drugs, and microgripping. The structure’s fold is encoded with alternating oblique patterns of ferromagnetic domains, hollow cross encoded with alternating ferromagnetic domains along the perimeter, quadrupedal and hexa-pedal structures enabled by folding of the magnetically active segments surrounding the magnetically inactive segments. A hexa-pedal structure demonstrated wrapping an oblong pharmaceutical pill, and a horizontal leap of a 3D auxetic structure upon sudden reversal of the applied magnetic field direction while attenuating the field strength by rotating a permanent magnet by 90° as shown in Figure 4(A). Another approach has been proposed to develop small-scale, flexible robots capable of fine control and diverse locomotion. This has potential applications in object manipulation, drug delivery, and minimally invasive procedures. The robot is made by patterning magnetic microparticles in an elastomer matrix. The control of magnetic particle reorientation and encoding can be done using selective UV lithography. This magnetization profile allows multiaxis and higher order bending at large angles. The physics-based model accurately predicts the change in shape due to magnetic actuation [74]. The study presents a system for patterning discrete 3D magnetization using a UV-curable elastomeric matrix composite. The materials are about 80 μm thick and have an actuating magnetic field of 200 mT for the “accordion” and less than 20 mT for all others. The schematic of an untethered multi-arm magnetic microgripper, magnetization profile, and working mechanism is shown in Figure 4(B){i}. Also, the conceptual model of a cargo transportation task is illustrated in Figure 4(B){ii}. The locomotion of the microrobot is demonstrated in silicone oil, as shown in Figure 4(B){iii}, which lifts the body weight and slows down shape changes of the gripper in response to an open-loop controlled magnetic field. Also, microrobot locomotion and velocity are slower compared to water environments. An untethered jellyfish-inspired soft miniature robot has multiple functionalities by generating a controlled flow of fluid around its body using elastomer lappets, which are magnetic composite [75]. These elastomer lappets are actuated in response to external oscillating magnetic fields. Predation-inspired manipulation of objects was achieved by the physical interaction of the soft microrobot’s motion and incurred fluidic flow due to it. The study explores the design and behavior of a jellyfish-inspired swimming soft millirobot, which mimics the deep clefts between two adjacent lappets. Kinematics and flow structures are achieved with the motion sequence, velocity and vorticity fields, and wake structures visualized by fluorescein dye. The robot and animal were compared in two kinematic metrics: bell fineness and lappet velocity, as shown in Figure 4(C).

Furthermore, a bioprinting method has been employed to fabricate a soft catheter ferromagnetic robot (FSCR) capable of minimally invasive in-the-place bioprinting based on computer-controlled magnetic actuation [76]. This robot is designed by dispersing ferromagnetic particles in a polymer fiber–reinforced matrix. It can be driven by a magnetic field, which is superimposed to get controlled and precise printing. Multiple patterns are printed on a planar surface, and minimally invasive hydrogel bioprinting is demonstrated on a rat model considering the natural organ’s nonplanar surface. The FSCR comprises a soft polymer matrix with dispersed hard-magnetic particles and a polylactide (PLA) reinforcing mesh with magnetic polarity programmed along its axial direction. Digital data instruct the numerical control strategy of the FSCR, and operations are instructed via the rotation and translation of four permanent magnets. *In vitro*, minimally invasive bioprinting of conducting hydrogel on porcine tissue surfaces is explored. The 3D scan and reconstruction of the curved surface of porcine tissue are conducted, and the 3D spiral printing path is designed on the reconstructed surface. Images of the minimally invasive bioprinting process of conducting hydrogel on porcine tissue at various times are shown in Figure 4(D). An untethered soft, quadruped, thin-film magnetic microrobot has been demonstrated for biomedical applications and small-scale micromanipulation [149]. The soft material makes it deformable and minimally invasive. This robot has four flexible magnetic legs and a nonmagnetic body that achieves controllable locomotion in an external magnetic field, which gives it the walking ability to cross obstacles in the stomach model. Gripping, transportation, and release of a microbead were demonstrated by controlling the conical angle of the externally applied magnetic field, as shown in Figure 4(E). A chain of magnetic soft robots that can form large assemblies by self-folding using a combination of magnetic and elastic energies in a stable configuration has been presented [77]. Repeated disassembly and assembly with programmable functions and shapes are achieved by pulling and pushing the soft robotic chain with respect to the catheter sheath. These chains are compatible with magnetic navigation and produce desirable functions and features that are difficult to obtain with surgical tools. The basic self-folding unit of MaSoChains is composed of rigid segments connected by soft segments, with small NdFeB magnets embedded at the same height as the surrounding surface. The folding process is initiated when a new segment is pushed out of the sheathing tube, where elastic and magnetic energies are stored. The MaSoChain is disassembled by pulling back with the guiding of the fixed tube. The three-segment MaSoChain structure is a foldable endoscope designed with three functional segments: the camera module, the magnet module with a large steering magnet, and the channel module through which a biopsy gripper can pass. The capsule endoscope can be actively steered using an external magnetic field. The process includes complete assembly, magnetic navigation, locking on the target, insertion of biopsy forceps, performing a biopsy, and retraction of the capsule, as shown in Figure 4(F). In addition to the above, the magnetic composite liquid metal droplet (LMD) was demonstrated by immersing a Galinstan liquid metal droplet in HCL, mixing it with 50 μm mesh-sized Fe powder, and gently stirring it at room temperature for 3 min. Such composite magnetic LMDs have reversible deformation, stretchability, and motion in response to applied magnetic fields. The composite magnetic LMD appeals to the potential application in reconfigurable electronics, soft µn-Bots, and micromotors [31]. Recently, a new class of magnetically controlled microscopic robots “diffractive robots” has been introduced that function at the visible light diffraction limit. The untethered microbots incorporated the nanometer-thick mechanical membranes, programmable nanomagnets, and diffractive optical elements to enable visible light diffraction and complex reconfigurations under millitesla scale magnetic fields. Such microbots demonstrated applications include subdiffraction imaging, tunable optical beam steering and focusing, and highly sensitive force sensing [150].

### 3.2 Electrically actuated soft µn-Bots

**Figure 5:**
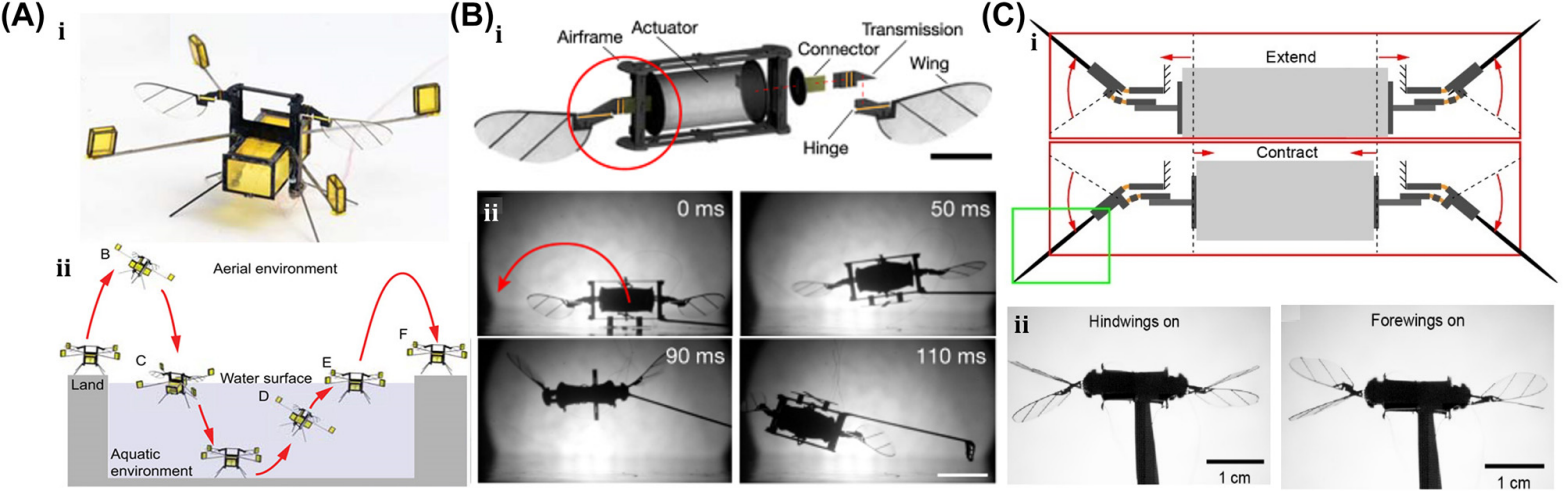
Electrically actuated soft bots. (A) (i) Schematic of a hybrid aquatic and aerial multimodal miniature-scale robot. (ii) Demonstration of robot locomotion, such as aerial hovering, air-to-water transition, swimming, water-to-air transition, impulsive take off, and landing. Reproduced with copyright permission from [78]. (B) (i) A CAD model of a flapping-wing robot, driven by a DEA. (ii) The unstable lift-off of a 155-mg robot driven by one DEA, which flips upside down due to unstable body pitch rotation. Reproduced with copyright permission from [80]. (C) (i) The perspective view of a flying insect-inspired miniature robot using two DEAs. (ii) Independent motion of two DEAs in either forewings or hindwings on condition. Reproduced with copyright permission from [81].

A 175-mg hybrid aquatic and aerial multimodal miniature-scale robot with versatile propulsion was developed to use the multimodal flapping technique to transition between aquatic and aerial environments [78]. Climbing and turning speeds achieved by them on acrylic surfaces are 0.3 cm/s and 23.6°/s, respectively. However, on a 30° inclined acrylic surface, its speed was 0.04 cm/s. This robot is lightweight, stable, equipped with controllable adhesion, and has limited sensing and actuation capabilities. The titanium top plate of the chamber includes porous apertures, while the sparking plate is made up of stainless-steel plates and a copper sparker. The robot is capable of aerial hovering, air-to-water transition, swimming, water-to-air transition, impulsive takeoff, and landing. It can transition from air to water, swim to the water surface, emerge from the water surface by capturing gas from electrolysis, and takeoff and landing as shown in Figure 5(A). The locomotion of a small-scale quadrupedal robot weighing 1.4 g attached to a 47 mg adhesion pad was demonstrated on inclined and inverted surfaces [79]. It measures 4 cm × 2 cm × 2 cm and weighs 47 mg with a compliant adhesive pad. A robot climbing a 30-degree gradient is demonstrated with an average speed of 0.04 cm/s, the robot climbs 13 cm in 311 s. The robot dragged down a horizontal surface while remaining attached via capillary adhesion. On a dry surface, the robot is pulled at 0.6 cm/s.

Recently, a flying insect–inspired miniature robot developed with the dielectric elastomer actuator (DEA)-based soft artificial muscle flapping–wing robot as shown in Figure 5(B){i}, which contains an actuator, connector, four-bar transmission, wing, and hinge capable of large deformation in an open-loop condition, showed relatively stable behavior [80]. This multilayer DEA has a power density of 600 W/kg and a resonance working frequency of 500 Hz, weighing 100 mg. When connected to the robot’s transmissions, the DEA is prestrained by 2 %, resulting in a static stroke angle bias of about 15°. The rotary wing stroke motion is translated from the DEA extension and contraction. The active and passive wing strokes and pitch motion of the robot are depicted in Figure 5(B){ii}. The flapping wing action is carried out at a frequency of 280 Hz, with time normalized to a flapping period. The passive wing pitch rotation is caused by the left-wing stroke rotation. The robot is tethered, requiring off-board amplifiers and external image processing to control flight. Utilizing such 4-DEA flapping wings as shown in Figure 5(C){i} demonstrates that the dragonfly-inspired aerial maneuvering robot weights 317 mg with a maximum lift-to-weight ratio of 1.49 at a 350 Hz operating frequency [81]. An at-scale soft robotic dragonfly is designed with two DEAs that individually drive a pair of wings. In reaction to a high-voltage driving signal, the DEA expands or contracts, connecting to linear four-bar transmissions. The DEA’s translational motion is turned into a rotating wing stroke action. The DEA directly controls the wing stroke action, while a compliant wing hinge passively mediates the wing pitch rotation. High-speed camera shots show tests with either the forewings or the hindwings turned on, as shown in Figure 5(C){ii}.

Subsequently, low voltage and high endurance flight for power-dense DEA-based soft miniature robots were demonstrated. It showed that a 143 mg DEA generates 1.15 mm displacement at 0.36 N force when operated at 400 Hz and achieved a lift-to-weight ratio of 3.7 at 500 V. Such DEA requires a high driving voltage compared to electromagnetic motors and piezoelectric bimorphs [151]. Also, the electro-luminance property was introduced into DEA to realize the concept of Firefly, which has the additional benefit of communication and motion tracking for such an aerial microrobot [152]. It consists of highly transparent electroluminescent particles embedded in four DEA, which light up simultaneously when electro-luminance particles are excited with a high-frequency electric field (>40 V/μm) generated at 400 Hz during the flying condition. The electrostatic flexible film actuator-based millimeter-scale robot was demonstrated in Figure 19(E) [153]. This, being robust and compact, has a power density of 61 W/kg. These actuators are like biological muscles, have a hierarchical structure with components ranging from electrodes to arrays to laminates, and are often constructed of flexible materials. As a result, these actuators may be designed to perform a wide range of manipulation and movement activities similar to actual muscle while being robust and compact. A typical actuator can provide 85 mN of force with a 15 mm stroke, a size of 28 × 5.7 × 0.3 mm^3^, and a weight of 92 mg. Two millimeters-sized robots, an ultra-thin earthworm-inspired robot, and an intestinal muscle-inspired endoscopic device for tissue excision demonstrate the use of these actuators. The earthworm robot conducts inspections in a small area with an onboard camera. They also performed tissue-cutting and piercing tasks, as shown in Figure 5(E){ii}. Furthermore, in recent years, electrical actuation techniques of soft materials like LMD (Galinstan, E-Galinstan) have been demonstrated for several applications, including microactuators, micromixers, micropumps, and tunable antennas [32], [33], [34], [35], [36]. An innovative composite LMDs based soft µn-Bots have been demonstrated by engulfing a functional magnetic framework into an LMD. The propulsion of LMD is based on the Marangoni effect, in which liquid metal experiences motion in an immersed NaOH solution due to the surface tension gradient formed on the LMD surface due to the applied electrical stimulus. LMD has been entrusted with actuating and enhancing cargo-carrying capability without destroying the intrinsic properties of LMD. An external electric or magnetic field can assemble or disassemble the magnetic framework from the LMD. Complex motions of liquid metal marble, like climbing, jumping, and rotation, have been demonstrated in NaOH solution environments with the applied electrical stimulus. Also, these LMD are loaded with drugs that can be released by laser heating [37]. Apart from above, a pioneering design of tiny motors based on LMD has been demonstrated to offer significant benefits, including ease of fabrication, maintenance-free, and relatively low cost over conventional motor technology. This technology holds immense potential in a wide range of fields, including soft electronics, soft robotics, microelectromechanical systems, and microfluidics [38].

### 3.3 Thermally actuated soft µn-Bots

**Figure 6:**
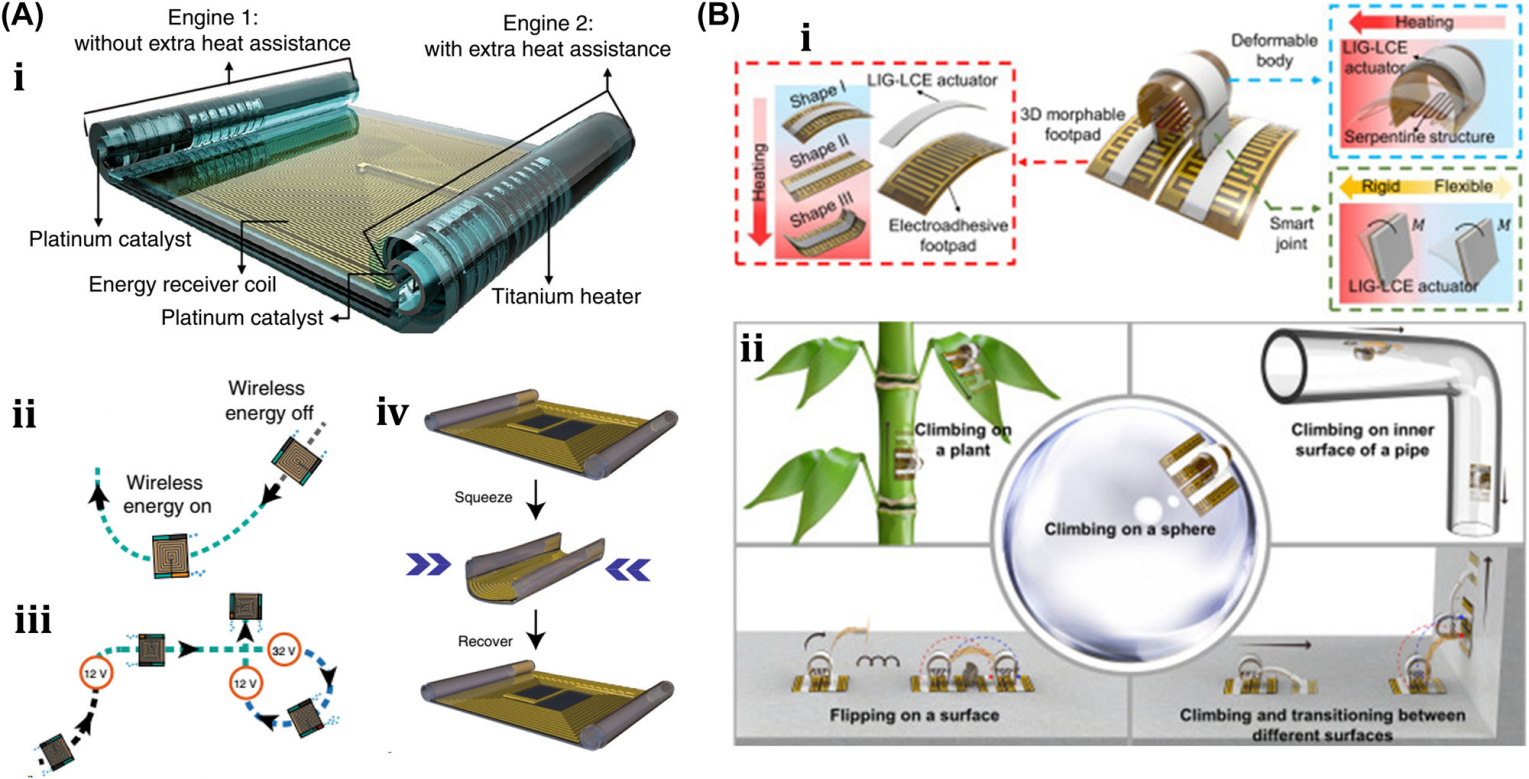
Thermally actuated soft bots. (A) (i) Design schematic and components of the MTJEMS. (ii) Trajectory of MTJEMS with 12 V supply. (iii) Trajectory of MTJEMS with different supply voltages. (iv) Shape recovery of MTJEMS. (v) Switching of micro-arm from “closed” to “open” with the remote-controlled inductive heating. Reproduced with copyright permission from [84]. (B) (i) Conceptual design and working mechanism of soft climbing microrobot. (ii) Soft microrobot capability of climbing on various surfaces, flipping on the surface, and transitioning between them. Reproduced with copyright permission from [6].

An untethered microscale system is developed to combine multiple functionalities such as actuation, motion, and communication has potential applications in drug delivery and robotics [84]. The system comprises flexible and rigid components, including sensors, actuators, microelectronic circuits, power supplies, engines, and controllers integrated into one platform. This flexible microsystem can control actuation and locomotion, driven by wireless power. The microsystem called the motile twin-jet-engine microsystem (MTJEMS) consists of two catalytic micro-engines shaped in tubes connected by a flat polymeric structure, as shown in Figure 6(A){i}. A square coil integrated into the platform receives inductive power through inductive coupling. The direction of the motion is controlled by locally heating the catalytic engine. This platform can also integrate LEDs and a thermos-responsive micro-arm to perform grasping and releasing tasks. MTJEMS are made in several steps, such as strained polymeric layer stacks joined by a polyimide layer, symmetric MTJEMS with a power receiver coil, heater wires, and symmetric Pt catalyst pads, and asymmetric MTJEMS with a power receiver coil, heater wires, and asymmetric Pt catalyst pads. The study explores the local heating effect of the MTJEMS, a catalytic engine, through wireless energy transfer and locomotion. Heating the Pt area in one catalytic engine results in a faster emission rate of O_2_ gas bubbles. Figure 6(A){ii} shows bubble generation without wireless energy transfer and with wireless energy transfer (voltage of transmitter coil, 12 V). The complex trajectory of the asymmetric MTJEMS for different voltages of the transmitter coil is also examined in Figure 6(A){iii}. The inductive heating of coils enables remote control of the opening and closing of the soft micro-arm on the MTJEMS. As for mechanical performance, the MTJEMS can also recover shape by squeezing its shape with a tweezer in the DI water environment depicted in Figure 6(A){iv}. The results provide insights into the catalytic engine’s performance and potential applications in various fields.

Researchers have developed another unique thermally actuated design for microrobots by integrating highly malleable 3D actuators capable of climbing and transitioning on complex surfaces or rough terrains [6]. This small-scale voltage-driven soft actuator consists of liquid crystal elastomers (LCEs) and a bending 3D assembly that measures 10 mm and weighs up to 3 gm. It is capable of achieving an angle greater than 200° due to thermal effects. However, for climbing, it has a footpad with electro-adhesive properties. The conceptual design and illustrations of soft microrobots capable of climbing on both planar and curved surfaces are shown in Figure 6(B){i}. Their body length is approximately 6 mm. The microrobot can climb on various surfaces, flip over barriers, and transition between different surfaces, as shown in Figure 6(B){ii}, including climbing on glass ceilings, PI cylinders, glass spheres, and *E. aureum* leaves. It also shows three locomotion gaits: stepping gait on the same surface, transition-type flipping gait for transition, and forward-type flipping gait. Potential applications of these microrobots include search and rescue, environmental monitoring, and industrial inspection.

### 3.4 Chemical reaction driven soft µn-Bots

**Figure 7:**
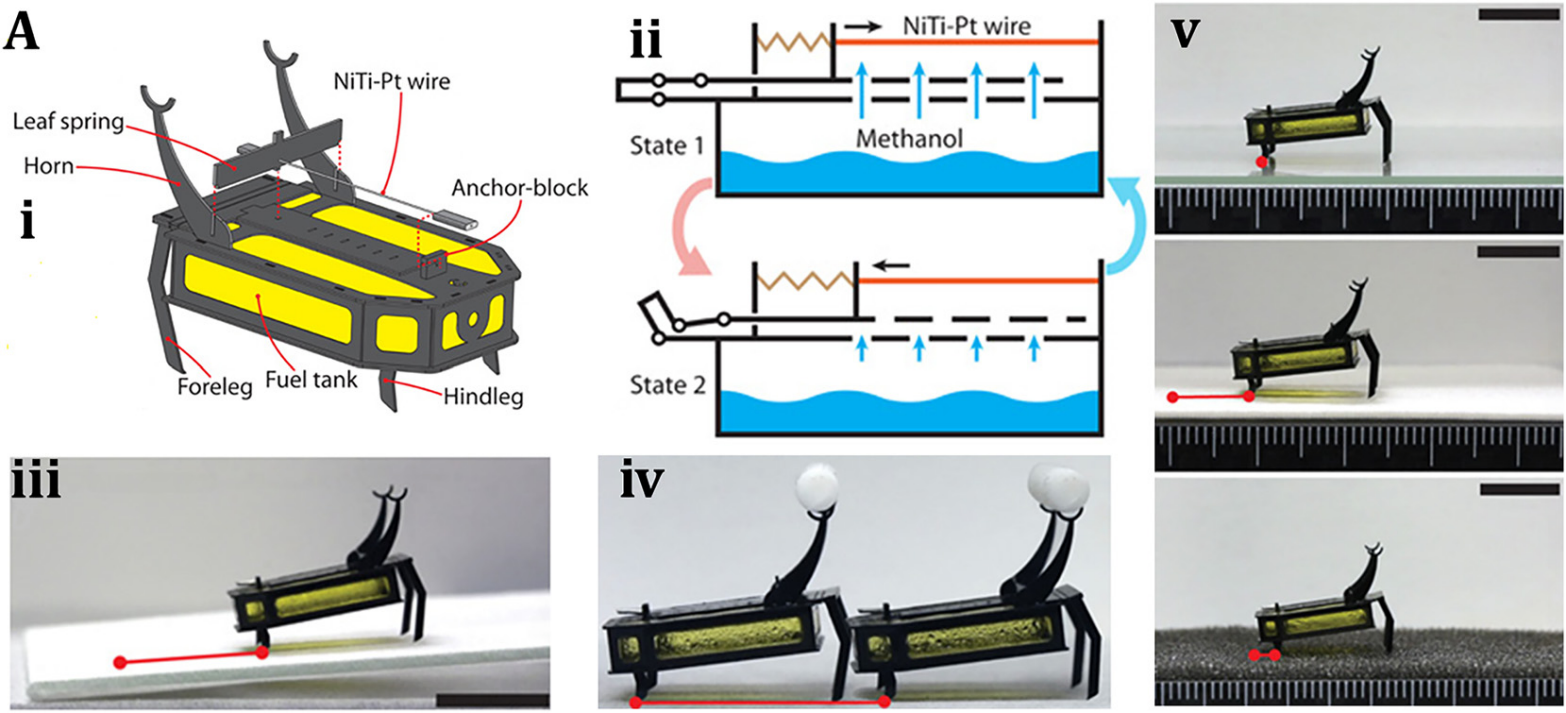
Chemical reaction driven soft bots. (A) (i) Exploded view of the RoBeetle’s assembly. (ii) Schematic diagram of robotic actuation mechanism. (iii) Crawling on the sliding surface. (iv) Crawling with payloads. (v) Crawling on glass, pacopad, and polyurethane charcoal foam surfaces. Reproduced with copyright permission from [82].

An 88 mg insect-sized crawling autonomous robot called RoBeetle was developed and powered by methanol’s catalytic combustion, which has 20 MJ/kg specific energy. The design consists of catalytic artificial micromuscles based on controllable Niti-Pt integrated with mm-scale mechanical control, as shown in Figure 7(A){i–ii}. The catalytic combustion of methanol under a Pt catalyst given below produces heat to raise the temperature of the nitinol SMA material, enabling martensite to undergo an austenite phase transformation.

$$\text{C}{\text{H}}_{3}\text{OH}\left(g\right)+3/2{\text{O}}_{2}\left(g\right)\to 2{\text{H}}_{2}\text{O}\left(g\right)+\text{C}{\text{O}}_{2}\left(g\right)$$

The contraction occurs when fuel contacts the catalytic surface of the Nitinol-Pt composite wire, enabling microrobot actuation. Microrobot crawling was successfully demonstrated on different surfaces (such as glass, pacopad, and polyurethane charcoal foam) under different atmospheric conditions, climbing on inclines of different slopes, transporting payloads, and engaging in outdoor locomotion, as shown in Figure 7(A){iii–v}. Also, a writable 6-mg radio-frequency identification (RFID) chip and a booster antenna were put on the robot to enable simple interactions and identification in the environment through a microcontroller [82].

Recently, another moisture-induced electric power insect-scale untethered robot inspired by cockroach locomotion has been demonstrated. The design, assembly, and working mechanism of a microrobot powered by moisture, along with a comparison of the mass path of a cockroach with the robot’s center, are shown in Figure 7(B){i}. However, the moisture-based energy harvesting device also examines their electrical output characteristics under different RH conditions. Under various atmospheric conditions, the robot captures atmospheric water using a hygroscopic gel and utilizes a redox reaction to generate electricity of around 1.4 V voltage and 43 mA current. The energy-harvesting device embedded in the microrobot became a battery-less, self-powered microrobot whose asymmetric structural design enables forward locomotion with an average speed of around 4 cm/s [83]. Also, a biomimetic self-power LMD motor inspired by mollusk has been demonstrated [29] and can be subsequently moved by eating the aluminum flakes in the NaOH solutions. Without external energy input, the LMD motor maintains its autonomous and rapid motion for over an hour. The pliable LMD allows the motor to self-deform in response to varying conditions, enhancing its adaptability for specific tasks. This self-powered LMD motor holds significant potential to advance the domains of self-motion in robotic design, microfluidic systems, and reconfigurable intelligent devices in the foreseeable future.

### 3.5 Acoustic field–driven soft µn-Bots

**Figure 8:**
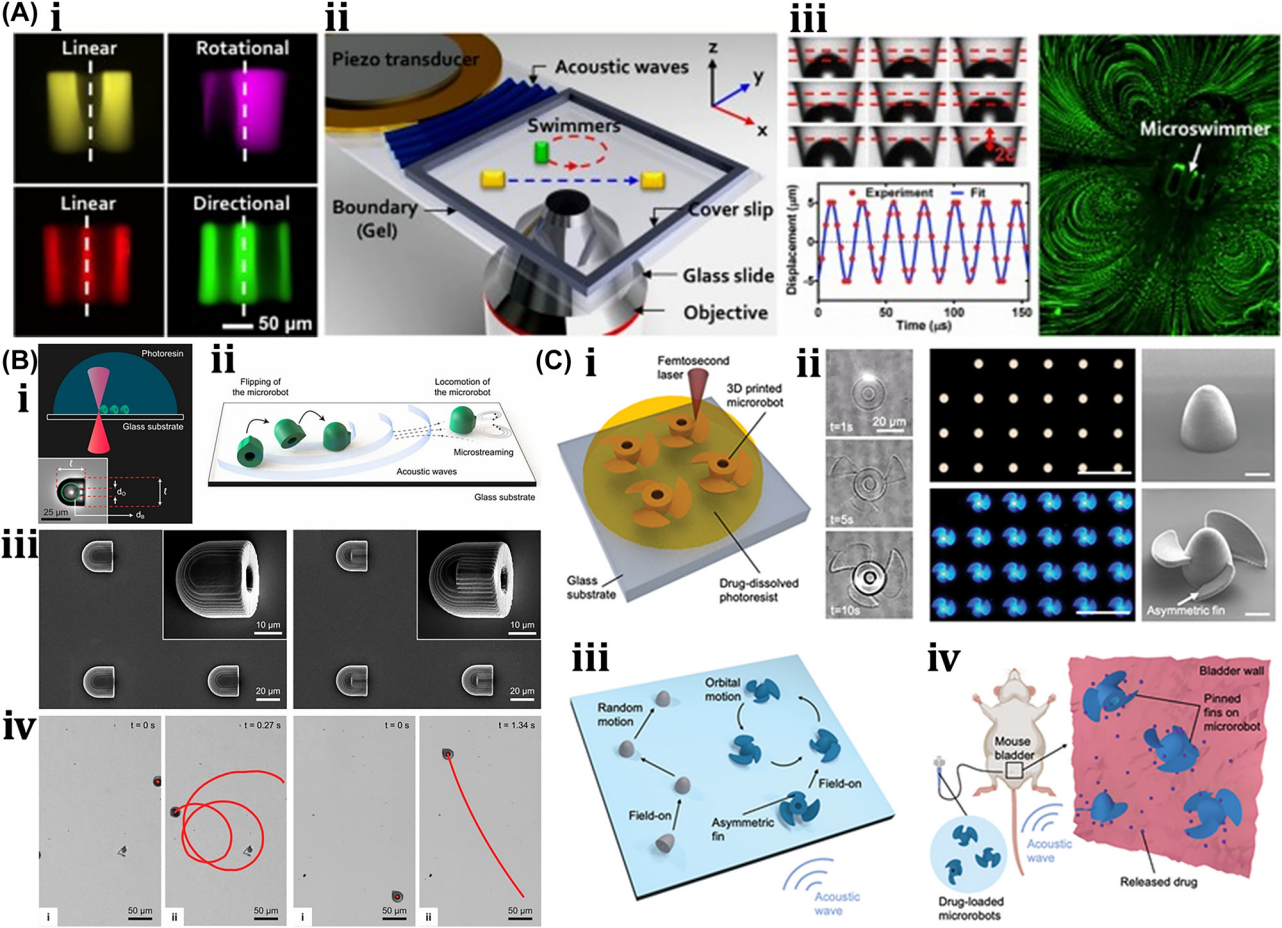
Acoustic field–driven soft µn-bots. (A) (i) Fluorescent images microswimmer. (ii) Microswimmer experimental demonstration with a piezoelectric transducer. (iii) Acoustic oscillation of microswimmer bubbles results in significant acoustic microstreaming in water. Reproduced with copyright permission from [85]. (B) (i) Nanoprinted microrobot on a glass slide. (ii) Nanoprinted microrobot propulsion mechanism under acoustic waves. (iii) SEM image of fully anisotropic and symmetric microrobot. (iii) Trochoidal random propulsion and directional forward motion of microrobots under ultrasound. Reproduced with copyright permission from [86]. (C) (i) Drug encapsulated 3D printed microrobot within its polymer matrix during cross-linking. (ii) Fluorescent microscope & SEM images of 3D-printed microrobot. (iii) Influence of asymmetric fins on the microrobots propulsion trajectories in response to acoustic waves. (iv) Demonstration of drug-loaded microrobots propulsion in mouse bladder excited by ultrasound. Reproduced with copyright permission from [87].

A unique method for regulating the motion of microswimmers using sound waves is “acoustophoretic actuation,” which produces controlled fluid flows that can be used to push and manipulate them selectively [85]. The oscillatory motion of the trapped air bubble inside the polymer body of the swimmer propels it at resonance, enabling controlled, rapid rotational, and translational motion even in highly viscous liquids. A group of microswimmers can also be achieved by incorporating unique bubble sizes with resonance frequencies. The microswimmers were developed by exposing a PEG solution containing a photosensitive initiator called an oligomer solution to UV light through the swimmer’s mask. Also, the swimmers’ geometries and conical indents were controlled through UV exposure duration. The geometry and experimental design of acoustic microswimmers are emphasized by different types of swimmers: linear, rotational, and directional as shown in Figure 8(A){i}. A piezoelectric transducer delivers acoustic energy into a fluid-filled container lined with an acoustically absorbent putty surrounded by glass slides shown in Figure 8{A}{ii}. However, Figure 8(A){iii} depicts the acoustic oscillation of microswimmer bubbles causes significant acoustic microstreaming in water.

Another untethered, acoustically powered 3D-printed bullet-shaped microrobot was developed in which spherical air bubbles are trapped inside the body cavity resonated according to acoustic waves. The net fluidic flow steers the microrobot and induces attractive force toward the wall. A small fin is attached to the cylindrical body to enable unidirectional locomotion. Anisotropic coating with a soft magnetic nanofilm layer allows motion direction control under a uniform magnetic field. The combination of magnetic steering and acoustic power enables it to navigate and actuate the microrobot in hard-to-reach. confined areas minimally invasively [86]. The microrobots were built with two-photon lithography and displayed in water. Asymmetric microstreaming patterns formed by the pulsating microbubble and the planned “fin” structure were used to model the robot’s propulsion depicted in Figure 8(B){i–ii}. The totally symmetric and anisotropic designs were studied using scanning electron microscope images depicted in Figure 8(B){iii}. Under ultrasonic actuation, the symmetric microrobot’s trochoidal random propulsion path was observed, while the directional forward motion of the microrobot with a fin structure was detected. The fin structure was critical in establishing flow asymmetry, which allowed for unidirectional motion. The research looks at the resonance frequency of microbubbles trapped in the chamber of a robot. It consists of a microrobot array, a confocal image of the shell, and a microstreaming pattern with resonance frequency of *f*_res_ = 237 kHz. The microrobot is submerged in a fluid medium, and an acoustic pressure map is displayed. The researchers also measured the average speeds of 2 μm tracer particles at various frequencies, normalized by the piezoelectric sensor voltage depicted in Figure 8(B){iv}. Another novel approach using bubble-based microrobots with complex geometries can efficiently swim with nonlinear trajectories in a mouse bladder, pin to the epithelium, and release therapeutic drugs slowly [87]. The asymmetric fins on the exterior bodies enable rapid rotational motions, and the encapsulation of dexamethasone in the microrobots tempers inflammation. These drugs carrying microrobots are 3D-printed with individual air bubbles using two-photon lithography depicted in Figure 8(C){i–ii}. Microrobots were stimulated with an acoustic traveling wave using a silicone spacer between the transducer and sensor. PBS was used as the propulsion medium, increasing air bubble stability due to increased surface tension. A sinusoidal AC signal was applied to the piezoelectric transducer, causing the microrobots to reorient, resulting in an air/liquid interface parallel to the substrate. This reorientation is due to secondary Bjerknes forces, which are attractive between the air bubble and the substrate depicted in Figure 8(C){iii}. Asymmetric fins aid in a variety of propulsion trajectories. Drug-loaded microrobots reduce inflammation in bladder tissue depicted in Figure 8(C){iv}. When stimulated by ultrasound, the bubble-based microrobots migrate through a mouse bladder until they come into contact with the bladder wall. Fins on the microrobots strengthen their pinning on the wall, allowing the drug to be gradually released to treat immune cells indefinitely. This system has the potential to provide an efficient medication delivery strategy in complex biological contexts.

## 4 Optically driven soft µn-Bots: principle & mechanisms

In the continuance of Section 2.6, this section explores the principles and mechanisms of light-driven soft µn-Bots that are categorized primarily into rigid and nonrigid types. The rigid type soft µn-Bots are typically driven with an optical tweezer, optical trapping, momentum transfer method, optochemical reaction, etc. However, the nonrigid type soft µn-Bots have the tendency to deform, including the photothermal effect and photomechanical effect.

### 4.1 Optically driven rigid µn-Bots

#### 4.1.1 Two-photon polymerization technology for the fabrication of rigid µn-Bots

The two-photon polymerization (2 PP) technology is the most prevalent method for fabricating 3D-printed rigid microrobots. In 1931, Goppert-Mayer et al. first explained 2 PP theoretically [154], and Kaiser et al. experimentally validated it theoretically 30 years later [155], [156].

**Figure 9:**
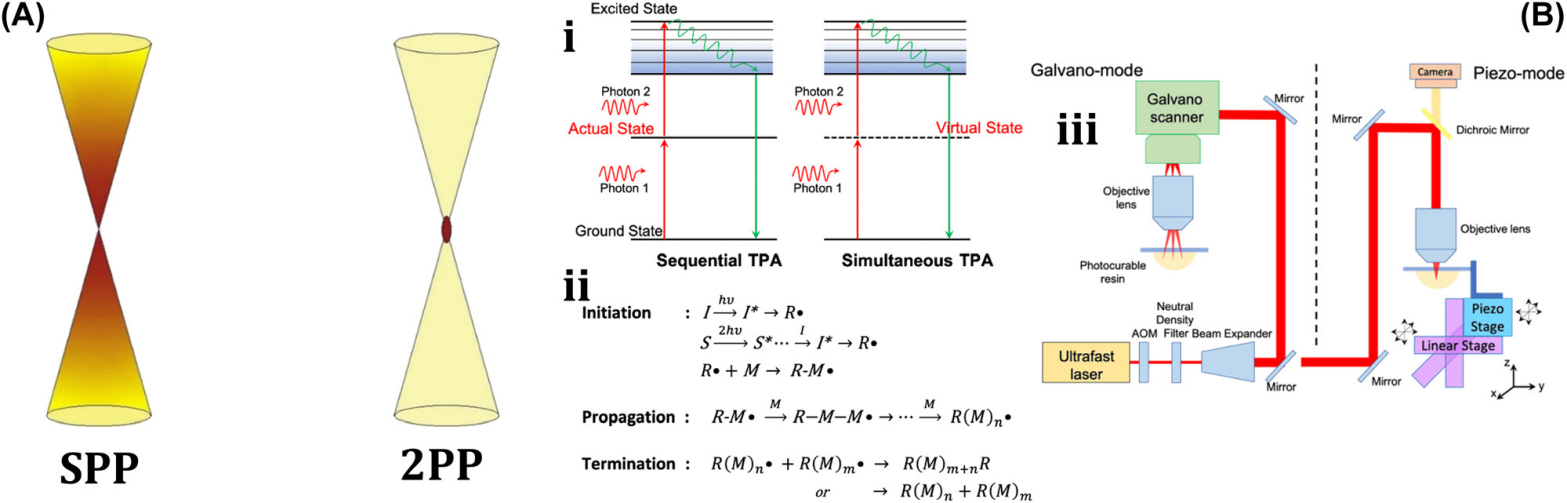
Polymerization mechanism. (A) Conceptual diagram of single-photon polymerization (SPP) and two-photon polymerization (2 PP). Reproduced with copyright permission from [158]. (B) (i) Two photon absorption mechanisms. (ii) Process steps for 2 PP. (c) Schematic of 2 PP device setups. Reproduced with copyright permission from [159].

Typically, 2 PP is a micromachining technology that depends on the nonlinear interaction of photosensitive resin and a femtosecond light pulse [157]. Figure 9(A) shows single-photon polymerization (SPP) and two-photon polymerization (2 PP) [158]. Unlike standard single-photon polymerization, the 3D printing method is similar to 2 PP laser direct writing and also involves the 2 PP principle mechanism. 2 PP techniques involve the photopolymerization phenomenon caused by the two-photon absorption of the material. Two-photon absorption (TPA) occurs when a material molecule absorbs two photons at the same instant in time, which involves two absorption processes: sequential absorption and simultaneous absorption. Figure 9(B){i} shows the sequential absorption process that involves the transition of an electron from the intermediate state to the excited state. The first photon absorption transforms the absorbing material into an intermediate state, and then the second photon absorption transforms it into an excited state. The other simultaneous absorption process is also shown in Figure 9(B){i}, where the electron absorbs two photons of quantum states at the same time and is excited to a higher state without undergoing an intermediate state.

Figure 9(B){ii–iii} shows the primary mechanisms and processes involved in 2 PP technology, which have been recently used in the micromachining of 3D-printed rigid polymeric microrobots [159]. The light-driven lobed micropump fabricated through 2 PP techniques can be used for fluid delivery by controlling two rotors with laser light [160]. A lightweight turbine similar to a micromotor, through the 2 PP techniques, efficiently transforms light energy into driving torque compared to earlier reported rotors and can be potentially used in the applications of fluid pumping and mixing [161].

#### 4.1.2 Optical tweezer enabled rigid µn-Bots

In 1970, Ashkin et al. pioneered optical trapping, which became a burgeoning field that was accredited with the 2018 Nobel Prize in Physics [162]. Optical trapping showed the capability of precisely manipulating differently shaped micro/nanoscale objects and materials like living cells, polymerized microspheres, and metal nanoparticles. So, the microrobot examples covered in this section are all controlled by optical trapping or optical tweezers (OT).

**Figure 10:**
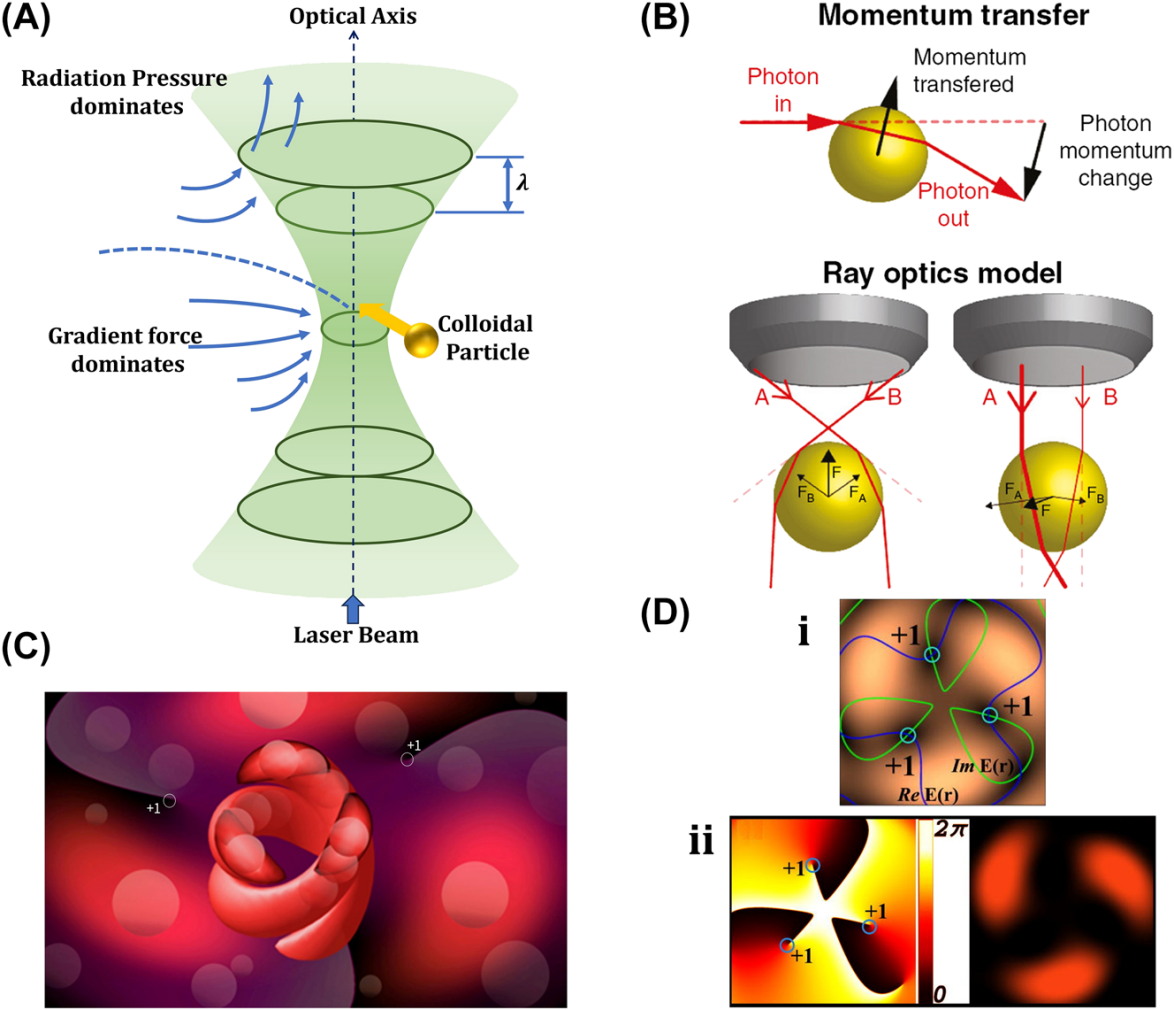
Fundamentals of optical tweezer mechanism. (A) Particles trapping through OT assisted strongly focused Gaussian beam [108]. (B) Illustration of momentum transfer between photon to particles and ray optics model for the gradient of forces due to light. Reproduced with copyright permission from [109]. (C) 3D OT demonstration induced by multiple plane waves. (D) Simulated profile for rotation phase and intensity of 3D OT. Reproduced with copyright permission from [110].

**Figure 11:**
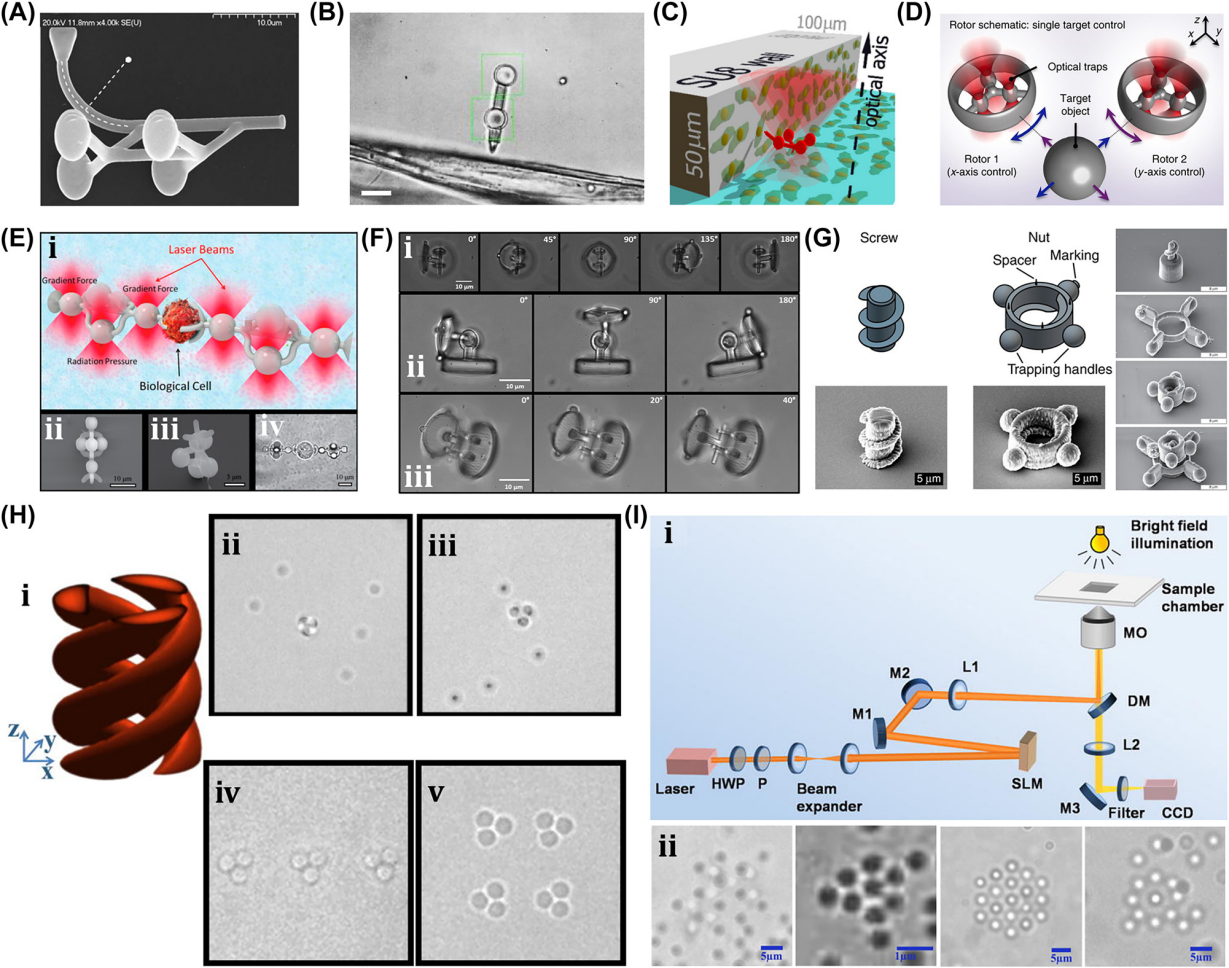
OT-driven rigid µn-Bots. (A) SEM image of 2 PP structured cylindrical probes. Reproduced with copyright permission from [111]. (B) Probe for the vibration measurement of floating cotton fibers in water. Reproduced with copyright permission from [112]. (C) Probe for the elasticity modulus measurement of cell membrane. Reproduced with copyright permission from [113]. (D) Microrotor to capture single target object. Reproduced with copyright permission from [92]. (E) Fabricated microclamp and their capturing process of cells. Reproduced with copyright permission from [114]. (F) Articulated microrobot motion and manipulation at several angles. Reproduced with copyright permission from [115]. (G) Structure, capture, movement and assembly of micro screw & nuts. Reproduced with copyright permission from [116]. (H) (i) Schematic of helical stacked microrotors profile. (ii) Tapping. (iii) Rotational locomotions. (iv-v) Multiple microrotor locomotions. Reproduced with copyright permission from [110]. (I) (i) Experimental setup of 3D OT. (ii) Optical trapping of in various lattice patterns. Reproduced with copyright permission from [117].

The generic OT system is developed with a laser, a microscope, and a pair of optical devices. Figure 10(A) shows that the schematics OT utilizes a strongly focused Gaussian beam to generate the optical operational force to operate and control the 3D-printed hard microrobots made of 2 PP technology [108]. However, the spheroid noticed by the researcher is the most stable capture, which has been validated through the use of beads of different materials and sizes; further, such shapes and structures could be utilized as handles, probes, and many more. The OT shown in Figure 10(B) ensures the momentum transfer between the photons and particles through the light–matter interactions [109]. Near the focus, the laser beams generate a well that induces potential due to repulsive and gravitational force interactions. A high refractive index (RI) object can be grabbed near the beam focus with the application of a strong gradient force. A 3D trapped OT was developed with the individually tunable 3D helical pattern designed by superposition of multiple plane waves that can be phase-engineered with a programmable spatial light modulator, as shown in Figure 10(C){i} [110]. Figure 10(C){ii} shows the simulated profile of the rotating phase and intensity of the triple arm rotor trap induced by a 3D helical pattern. Such an optical tweezer can be utilized for optical trapping and helically stacked micromotors. A few examples of OT-driven 3D-printed hard microrobots are shown in Figure 11, which are mostly fabricated with 2 PP techniques and typically small in size. The first probe designed for the surface topography analysis consists of four cylindrical capture handles that could be driven themselves and manipulated through OT as shown in Figure 11(A) [111]. In the surface topography measurement of the sample, the probe tip is close to the sample, where the tip trajectory is evaluated from its transverse scanning. After 1 year, an OT-driven probe was also developed for scanning the profile morphology of the samples [91]. A microtool-like probe was devised to detect the cotton fibers vibrations floating on water, as shown in Figure 11(B) [112]. It may be used in the identification of yeast, bacteria, and other self-vibrating specimens. A probe for cell elasticity measurement was developed, and their experiment schematic is depicted in Figure 11(C), where the probe detected the lateral growth of endothelial cells on the vertical polymer wall [113]. The obtained value of elastic modulus in this experiment is similar to other reported methods. Also, the probe prevents cell damage in the optical capturing process by using a spherical light capture point. Instead of serving as a probe, OT drives could use 3D-printed hard microrobots to capture the target objects. The OT-driven microrotor depicted in Figure 11(D) shows the capture of a single target object with the formation of six traps by utilizing two OT-driven microrotors [92]. Also, it can be repositioned in a 2D plane in any direction, with the direction and rotation rate of the microrotor controlled through the OT. However, the multiple target object capture relies on five OT-driven microrotors that limit the four DOFs of the target object for their capture. An OT-driven clamp was developed to capture live cells. It comprises a fork to clamp the cells and a sphere for optical capture, and Figure 11(E) shows that the cells can be captured by the OT-controlled forked clamp [114]. The indirect manipulation of biological particles with the hard microrobot avoids cell damage from the optical tweezer. Also, it has limitations on translational and rotational motion in two dimensions only, whereas rotation in three dimensions is a critical requirement in many cell operations. So, an OT-driven articulated microrobot was developed, as shown in Figure 11(F), which can spin at a different angle under the control of OT and is also capable of cell manipulation [115]. That makes it useful in sophisticated biological applications that need three-dimensional spatial control, such as nuclear transfer, embryo injection, single-cell analysis, and others. The OT-driven microrotor has four optical capture points for moving, capturing, and screwing the nuts, as shown in Figure 11(G) [116]. Also, this technique could be helpful in a microfluidic chip for fluid mixing and pumping. 3D optical twister-assisted reconfigurable helically stacked microrotors demonstrated through the light field generated with the interference of phase-engineered multiple plane waves [110], as shown in Figure 11(H){i}. Figure 11(H){ii–iii} demonstrates the trapping of 2 μm silica particles and the rotational locomotions of stacked helical microrotors with the 3D optical twister, respectively. Further, the multiple OT-driven microrotors are generated in rows, as shown in Figure 6(H){iv}, and in two rows, where their rotation directions are opposite each other, as shown in Figure 11(H){v}. Such microrotors have numerous applications, including micropumps, micromixers, microfluidics, and particle sorting. Similarly, 3D optical twister–assisted dynamic and reconfigurable optical trapping of microparticles was also demonstrated [117]. The experimental setup of dynamic and reconfigurable optical trapping is shown in Figure 11(I){i}. Figure 11(I){ii} demonstrates the trapping of the 2 μm silica beads and 0.5 μm polystyrene spheres in various lattice patterns. Such optical trapping can be efficiently utilized in biomedical applications, including kidney stone removal, drug delivery, etc. OT is easy to control and has potential applications in biomedicine. Also, it can precisely control the microrobot’s position within a position error of 20 nm. Typically, light-induced force in OT is in the order of pico-newton (*pN*), which effectively drives only tiny objects, so the perspective of microrobots is constrained by the material, structure, shape, and their applications. In addition, OT could be able to control numerous microrobots simultaneously, resulting in complex functionality and making it costlier due to the necessity of the hardware for beam modification and a software controller. To circumvent these constraints, the researchers introduce other light-induced driving mechanisms, including photo-induced dielectric electrophoresis, the photothermal effect, and a momentum transfer drive mechanism, to power the microrobot.

#### 4.1.3 OET-assisted rigid µn-Bots

**Figure 12:**
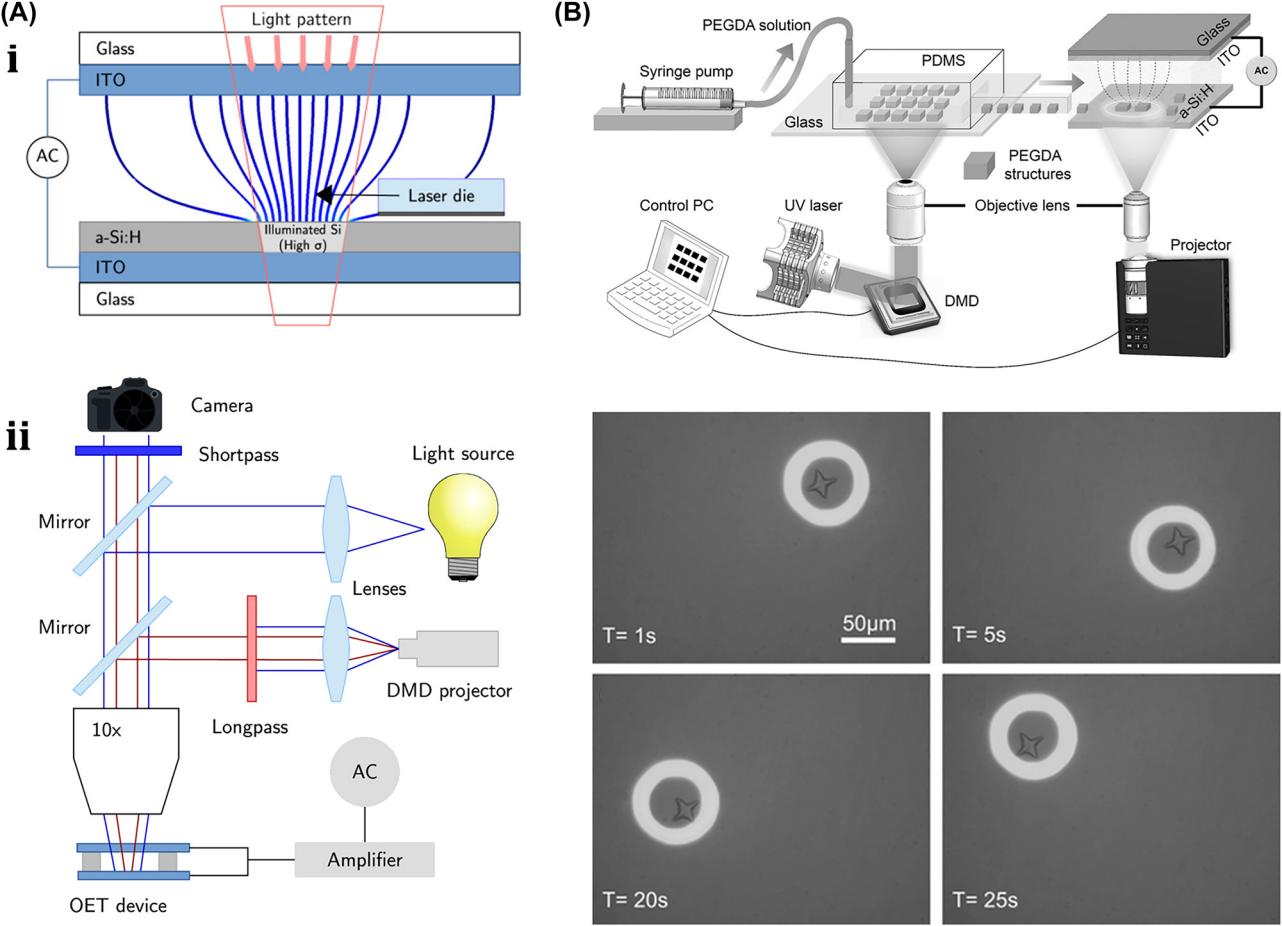
OET-assisted rigid µn-Bots. (A) (i) Generic diagram of OETs indicating asymmetrical distribution of electric field. (ii) Device required for OET setups. Reproduced with copyright permission from [119]. (B) OET device setup for the manipulation of star shaped hydrogel microstructure manipulation. Reproduced with copyright permission from [120].

In 2005, Chiou et al. discovered the opto-induced electrophoretic technique (OET), which gained notable interest and has become a promising technology in µn-Bots design [118]. The µn-Bots driven with OET utilized the opto-induced DEP forces that integrate together the features of optics and electronics in the programming and contactless driving of µn-Bots. Compared to the OT system, the light source used in the OET system does not have a strange requirement, so an LED source with a digital micromirror device (DMD) can be used as a light source. The OET mechanism depends mainly on the distinctive properties of photoconductive substrates, which have a high impedance similar to resistors in the dark. However, when the photoconductive substrate is exposed to light, its impedance substantially decreases, and it becomes analogous to the conductor. So, Figure 12(A){i} shows an induced asymmetrical electric field in a liquid medium on the photoconductive substrate caused by a photoactivated virtual electrode developed through the dark and light patches created on the photoconductive substrate [119]. The device used in the OET drive, as shown in Figure 12(A){ii}, is easy to operate and can drive a large microrobot with a strong control force compared to OT.

Figure 12(B) shows a star-shaped hydrogel microrobot that utilized OET for transportation [120]. Also, the Tetris architecture has been made of a swarm of hydrogel microrobots that use assembly and manipulation through OET. Due to the biocompatible nature of hydrogel, hydrogel microrobots can encapsulate various cells. Also, it can be grouped into structures of desired shapes and sizes, which is extremely valuable in the application of microscale tissue engineering. The OET offers the benefits of rich biocompatibility, flexibility, and high efficiency. The OET-based system is able to sense complicated operations and causes numerous µn-Bots to work in tandem and autonomously. Such a system requires less equipment and can be implemented even with a basic microscope and low-cost optical projector. Such technology will greatly benefit the field of biological sciences.

#### 4.1.4 Momentum-assisted rigid µn-Bots

Momentum is a physical quantity that quantifies the motion attributes, motion capabilities, and motion state of an object. It includes motion properties such as magnitude and direction that can be transferred from one object to another. The researcher has explored the potential to drive the micro/nano-object with photon momentum transfer [163]. A light-driven, reflective, surface-coated micron-shaped wedge can use the momentum of incident photons as a driving force to drive itself [69]. When the surface of the top wedge is illuminated by NIR laser light, the deflection of light from its reflected surface causes a change in momentum, resulting in the movement of the wedge in a particular direction. Micromotor rotational motion with laser light has been demonstrated with the nanoscale gold motor sandwiched between SiO2 square microdisk that can be spun by laser light [121]. It rotates by transforming the photon’s angular momentum into torque.

**Figure 13:**
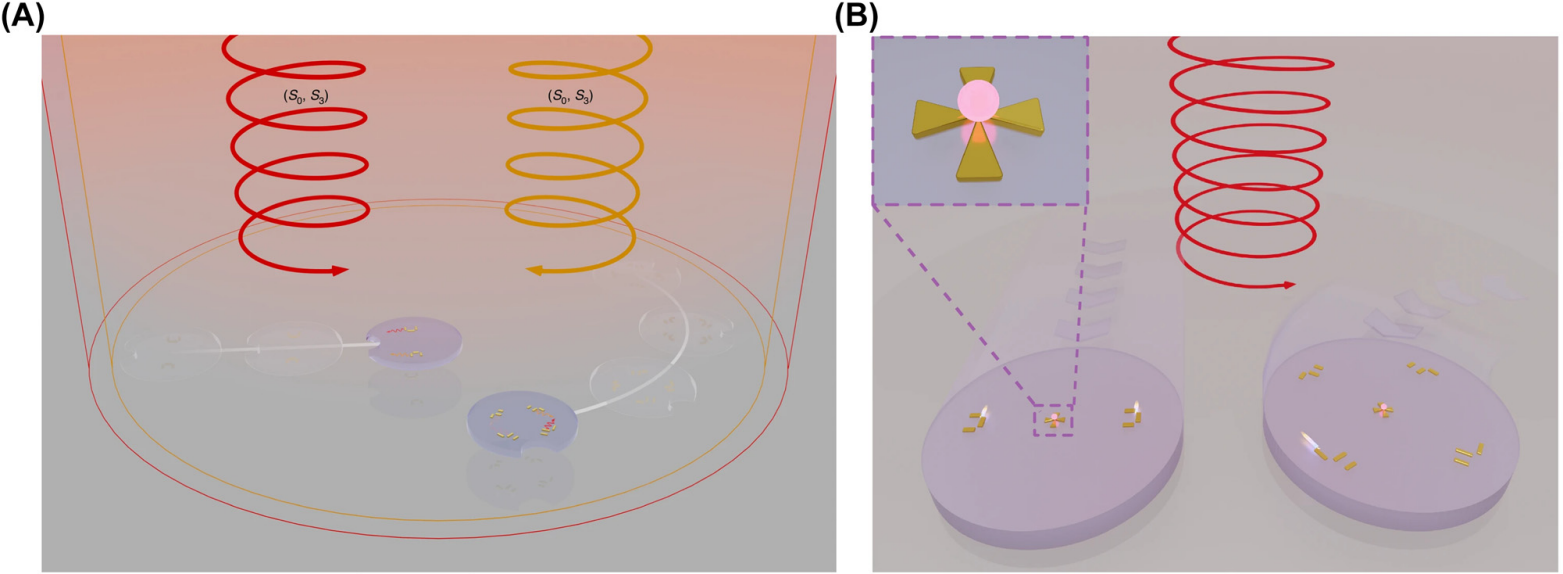
Momentum-assisted rigid µn-Bots. (A) Light-driven microdrones. Reproduced with copyright permission from [165]. (B) Manipulation of plasmonic microdrones embedded with tweezer under the circular polarized light. Reproduced with copyright permission from [166].

The optical metasurface-powered microscopic metavehicles have been designed to operate in water through 1,064 nm plane-wave illumination [164]. These metavehicles harness the momentum of light for propulsion and steering, showcasing the integration of metasurface design with light-driven motion at the microscale for complex patterns, self-correcting motion, and an application as transport vehicles for microscopic cargoes, which include unicellular organisms. Furthermore, light-driven microdrones equipped with individually addressable chiral plasmonic nanomotors as shown in Figure 13(A) have been designed, where two unfocused, circularly polarized beams at distinct wavelengths (830 nm and 980 nm) can be precisely controlled into two dimensions with all three independent degrees of freedom [165]. The approach, mimicking macroscopic quadcopters, overcame the limitations of optical gradient traps by enabling orientation-independent actuation using photon recoil from circular polarization. Recently, a gold cross antenna-based plasmonic nano-tweezer integrated into a microdrone transformed it into a microrobot capable of precise optical trapping and manipulating single nanoparticles as shown in Figure 13(B) [166]. The motors and tweezers were powered simultaneously by circularly polarized light, enabling seamless trap-transport-release cycles of fluorescent nanodiamonds, a feat unattainable with traditional optical tweezers. This advancement underscores the synergy between propulsion and functional manipulation in light-actuated µn-Bots.

The significant benefit of momentum-based µn-Bots actuation is its long duration due to a continuous energy supply for motion. Light provides the driving energy for the µn-Bots that use it to move in the direction defined by their position and shape. Such an approach is much more efficient than others and particularly well suited to analyzing µn-Bots swarm motions.

#### 4.1.5 Photothermal-induced manipulation

**Figure 14:**
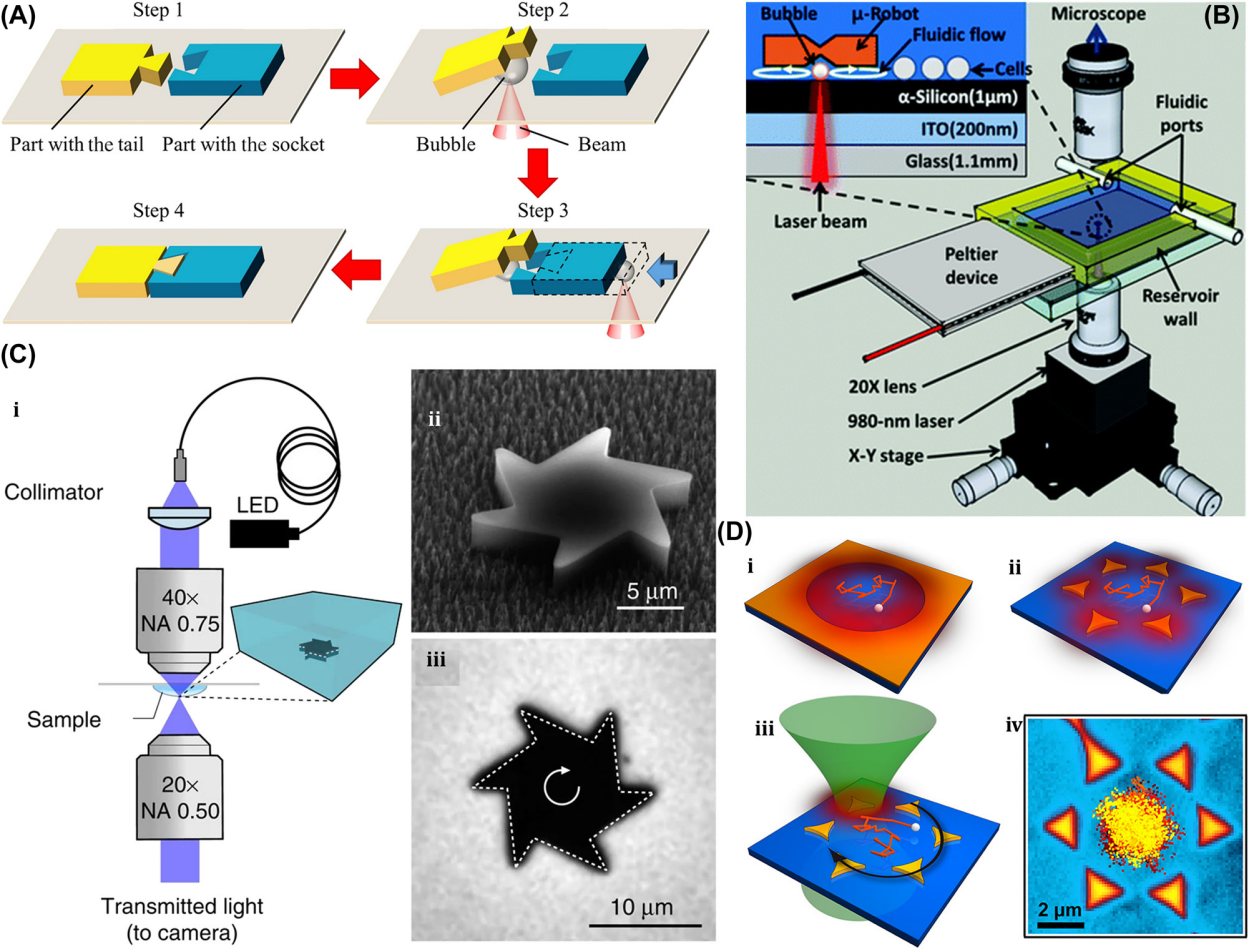
Photothermal-induced manipulation of rigid µn-Bots. (A) Process steps of light-induced bubble-driven assembly of two microparts. Reproduced with copyright permission from [122]. (B) Experimental setup of bubble-driven hydrogel, PEGDA disc microrobot. Reproduced with copyright permission from [123]. (C) (i) Experimental setup for microgear. (ii) SEM image of fabricated microgear. (iii) Microgear rotational motion under LED illumination. Reproduced with copyright permission from [126]. (D) (i) Closed gold microstructure. (ii) Open hexagonal microstructure. (iii–iv) Single particle capture in hexagonal array microstructure under the influence of laser. Reproduced with copyright permission from [127].

Photothermal effect–induced optical manipulation could also be capable of driving 3D-printed hard microrobots. Such microrobot driving techniques use photothermal material to convert absorbed light into heat, which further develops a temperature gradient with the heat diffusion around the microrobot, causing motion in the microrobot. The microrobot motion with surface bubbles is shown in Figure 14(A), where the laser beam is focused on a chip made of layered amorphous silicon that converts absorbed light into heat [122]. The produced heat generates bubbles on the surface, enabling the movement, fixing, raising, and assembling of a tiny object. For example, assembling parts of a pair of dovetail miniatures begins with placing the bossed and dented parts together. Then, the laser light induces a bubble robot underneath the bossed part and lifts it up like a jack. Similarly, another bubble robot pushed the dented part into the specified position. At last, the bossed part falls with the dissolution of the first bubble and achieved integrated assembly of two microparts. Furthermore, the assembly of two gears, the three-tooth and the four-tooth gears, is also demonstrated respectively. Moreover, it demonstrates the assembly of chains and automobile structures [122]. Similarly, a laser-powered, bubble-driven microrobot made of hydrogel, PEGDA was also developed in a disc shape, and the experimental setup is shown in Figure 14(B). With the focus of the laser on the substrate of the microrobot disc center, it caused light absorption to induce bubbles only in the concave part of the microrobot because it is made of the α-Si layer, resulting in the transformation of light energy into heat energy. The assembly of polystyrene beads in various patterns as well as the module of the cell-loaded microgel in the desired pattern were achieved with microrobot motions [123]. A submillimeter robot named ChevBot was developed that is capable of moving on a dry surface [124]. The microbot motion achieved with laser irradiation involves photothermal to mechanical energy conversion. The experiment with the ChevBot microrobot on the dry substrate revealed its speed of motion to be about 46 μm/s with the 2 W laser power and its 60 kHz repetition frequency. However, its DOF is limited because there is only one actuator.

To address the DOF, a light-driven microbot named Serpenbot has been developed that has a pair of thermo-induced resonance legs and can also operate in dry environments [125]. Also, the microrobot can achieve straight or turned motion with laser irradiation of distinct frequencies. The microrobot’s surface, coated with photothermal materials as exposed by the laser, will create the temperature gradient that results in a motion to drive it. An asymmetrical microgear in which one side is uniformly coated with amorphous carbon to enhance light absorption is designed as illustrated in Figure 14(C){ii} [126]. Figure 14(C){i and iii} shows the microgear experimental setup where microgears rotate steadily about their centers, and they can be driven by the generation of a temperature gradient in the fluid around the gear placed in the LED light illumination. The optically heated gold nanostructures have been demonstrated to trap the individual particles by inducing strong temperature gradient fields in the liquid [127].

As Figure 14(D){i-ii} shows, it involved two distinct gold nanostructures: a gold film with circular holes and a hexagonal array with a single gold pad. Figure 14(D){iii-iv} shows the single particle capture mechanism, which involves selectively shining a laser on a single gold pad of a hexagonal array at a time. The discussed driving mechanism adds the features of self-actuation and fuel-free operation to microrobot designs and has the advantages of biocompatibility, a large capacity, and precise control in the motion direction for quick loading and unloading. The user may benefit from leisurely microrobot control of start, stop, and speed with the position and intensity control of the laser.

### 4.2 Opto-chemical actuation of µn-Bots

The µn-Bots that utilize photochemical reactions for the propulsion force is termed as opto-chemical µn-Bots. Such µn-Bots are made of photoactive materials, and they require an environment of photochemically active reagents with the utilization properties of an optical source, including intensity, wavelength, and its polarization, to trigger the photochemical reactions. The notion is inspired by the survival of living microorganisms present in nature, including cyanobacteria and algae that convert solar energy into biochemical energy via the photosynthetic mechanism [2], [4]. Similarly, an opto-chemical µn-Bots developed with photoactive materials converts light energy into kinetic energy in the generation of propulsion forces, allowing it to move in the desired direction. The two preliminary morphological designs of opto-chemical µn-Bots are named the spherical Janus µn-Bots and the tube/bar/line µn-Bots, respectively [4]. A spherical Janus µn-Bots made of a solid metal sphere, where physical vapor deposition techniques are used to coat a thin layer of material over its local surface to make Janus particles. However, the coated surface of such µn-Bots consists of two distinct materials that result in two distinguishable reactions occurring on each of the Janus particle’s two surfaces and form an unbalanced gradient field for their propulsion. Since it is simple and easy to manufacture, also it can propel in pure water with an observable speed even in dim light without any need of surfactants has the additional benefit of the spherical Janus µn-Bots.

Another type of µn-Bots is a bar, line, or tube shape made of various materials that propels itself, causing an uneven gradient field due to the asymmetric reaction that occurs in the inner cavity and outside surface [4]. Moreover, the tabular µn-Bots are developed by self-crimping nanofilm or template-assisted technology that involves the deposition of one layer of photoresist on the silicon substrate, followed by the deposition of two layers of nanofilm under the vacuum. An internal stress gradient is induced in the double-layer film during its deposition process. The nanofilm is then released following the selective dissolution of the photoresist, and the stress gradient leads the film to coil into a tubular form. However, the majority of rod µn-Bots are developed with porous templates in which the holes of the template are loaded with metal materials either through physical filling or chemical reduction. At present, the aluminum oxide template is mostly used. The anticipated potential of tabular micro/nanostructures is utilized in the formation of propulsion or bubble recoil–based opto-chemical µn-Bots because the internal and external surfaces have distinct photoactivity, resulting in distinct diffusion behaviors in the confined inner void and infinite external space, respectively.

The optochemical µn-Bots propel itself mainly due to asymmetrical chemical reactions on its surface caused by light irradiation. It leads to an asymmetrical distribution in the ion concentrations, electron concentrations, bubbles, and heat, resulting in four propulsion mechanisms: self-diffusion, self-electrophoresis, bubble jet, and self-thermophoresis. The µn-Bots merely catalyze such a reaction above that consumes the surrounding solution for their propulsion but is not itself devoured.

#### 4.2.1 Self-diffusion propulsion

**Figure 15:**
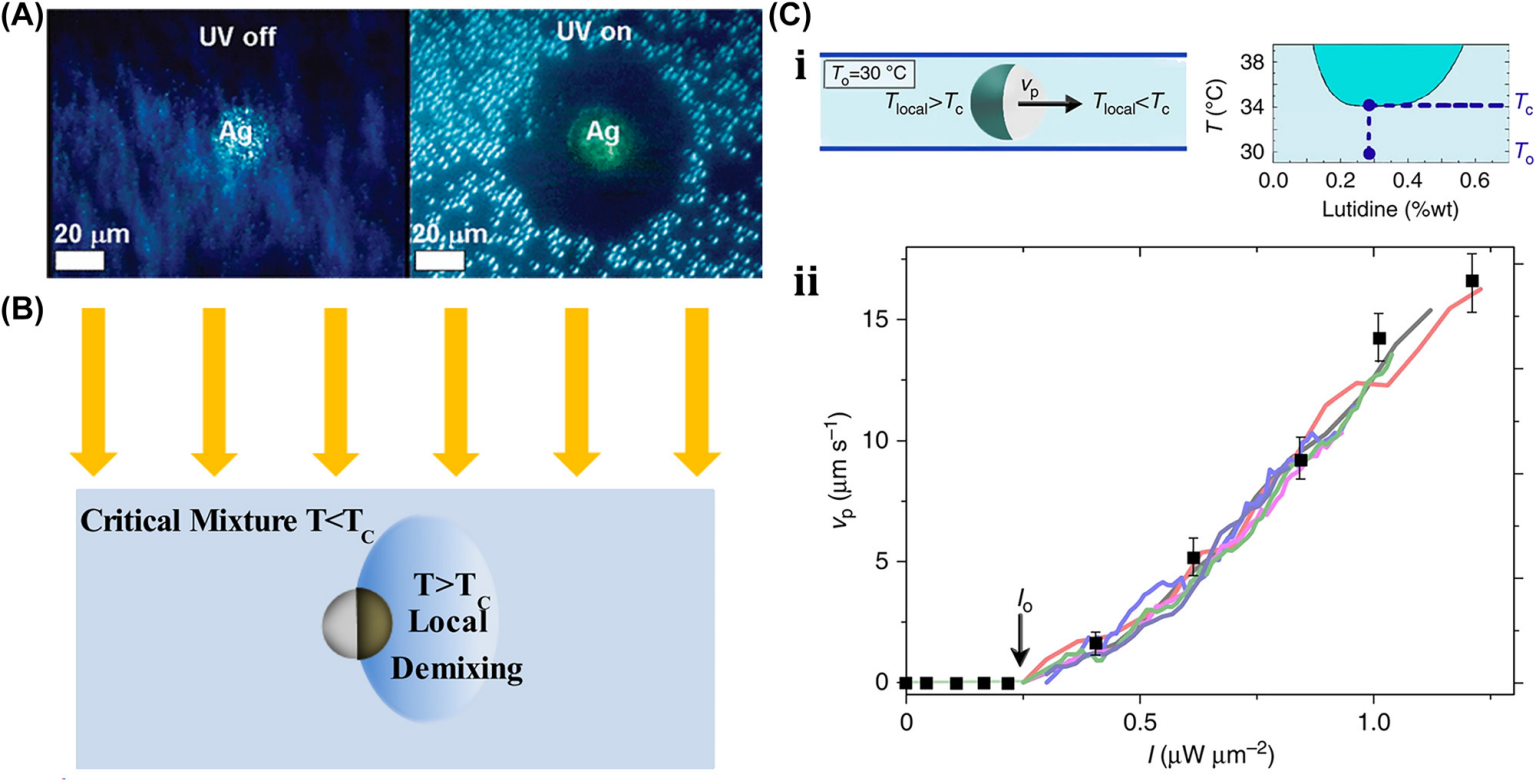
Self-diffusion–driven µn-Bots. (A) Silver catalyst–based microdisk motion in H_2_O_2_ solution under the influence of UV light. Reproduced with copyright permission from [88]. (B) Propulsion of Janus particle under the light illumination heated above *T*_C_. Reproduced with copyright permission from [90]. (C) (i) SiO_2_ spheres micromotor working mechanism. (ii) Micromotor propulsion speed versus intensity of light. Reproduced with copyright permission from [128].

Theoretically, Derjaguin et al. anticipated the diffusion motion in 1947 and demonstrated it experimentally [167]. The optochemical µn-Bots are mainly based on photochemical reactions, like conventional chemical reactions that involve consuming reactants to formulate the product. The self-diffusion propulsion mechanism in µn-Bots is driven mainly by the asymmetrical photoreaction ensuing on their surface under light exposure, which results in a gradient of product concentration, implying the diffusion of chemical compounds from a high concentration to a low concentration. For instance, in the case of an Au/WO_3_ microrobot, the product concentration on the surface of WO_3_ is higher than that of Au, causing diffusion phenomena to occur from the surface of WO_3_ to Au, which results in propulsion of the µn-Bots in the direction of Au. The first optochemical µn-Bots were demonstrated in 2006 [88], where UV light was utilized to generate the Ag^+^ and OOH^−^ ions that diffuse into the microrobot surface with distinct diffusion coefficients and cause microrobot diffusion. Figure 15(A) shows widespread interest in discovering numerous optochemical microrobot with the self-diffusion process. On the other hand, the light-induced catalytic reaction process by employing the semiconductors in the microrobot induces phase separation in the binary mixtures near their critical points, so they can also be able to swim as shown in Figure 15(B) [89], [90].

A micromotor was developed using SiO_2_ spheres coated with a carbon layer that absorbs light [128]. Figure 15(C){i} shows micromotors only remain stable at a critical temperature of 34.1 °C when submerged in the binary critical mixture of water-2, 6-lutidine. As the micromotor comes into light exposure, the solution partially decomposes due to increased carbon cap temperature, inducing a concentration gradient that leads to self-diffusion in the micromotor propulsion. It is observed that the micromotor has propulsion from self-diffusion due to the liquid resistance. Figure 15(C){ii} shows that only the Brownian motion is observed in the micromotor when the light intensity is below the threshold value *I*_0_; however, the micromotor motion will occur due to the self-diffusion phenomenon if the light intensity exceeds *I*_0_ and the carbon cap is heated above critical temperature *T*_*c*_.

#### 4.2.2 Self-electrophoresis–driven µn-Bots

**Figure 16:**
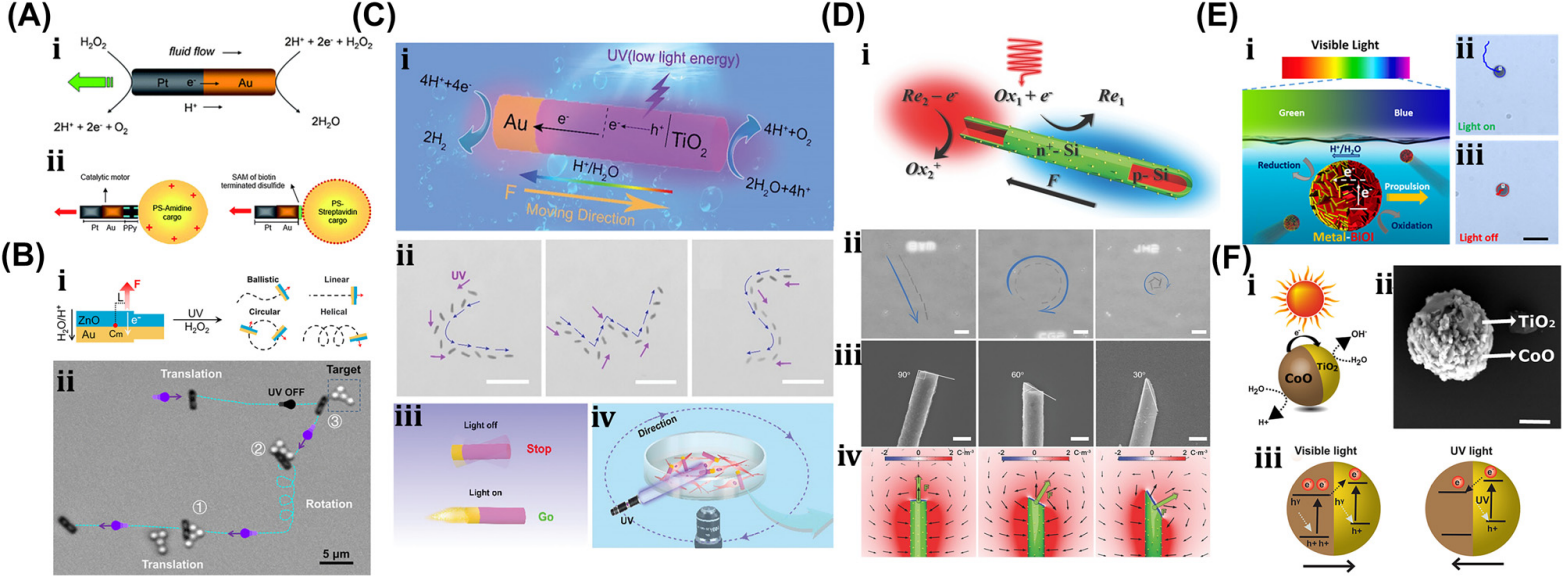
Self-electrophoresis–driven µn-Bots. (A) (i) Schematic and reaction mechanism of Pt/Au rod–based microrobot. (ii) Cargo loaded Pt/Au rod–based microrobot. Reproduced with copyright permission from [93]. (B) (i) Au/ZnO microrobot schematic and their modes of motions. (ii) Demonstration of motions mode for the cargo transportation. Reproduced with copyright permission from [129]. (C) (i) Au/TiO_2_–based microwires actuation mechanism. (iii) Microwires motion trajectory follows the different letter shape includes. (iii) UV light assisted actuation of microwires. (iv) Neurons manipulation through microwires. Reproduced with copyright permission from [130]. (D) (i) *n* ^+^-Si and *p*-Si surfaces based nanowires. (ii) Trajectory of nanowires. (iii–iv) Fabrication of nanowires with different end faces and their resultant electric field profiles. Reproduced with copyright permission from [131]. (E) (i) Conceptual mechanism of metal-BiOI microrobot. (ii–iii) Motion trajectory of metal-BiOI microrobot under visible lights. Reproduced with copyright permission from [132]. (F) (i) Reaction mechanism of CoO/TiO_2_ microrobot under visible light. (ii) SEM image of CoO/TiO_2_ microrobot. (iii) Direction of propulsion force of CoO/TiO_2_ microrobot under visible and UV light due to change in energy gap. Reproduced with copyright permission from [94].

The self-electrophoresis–induced propulsion mechanism in the optochemical microrobot is caused by a concentration gradient in the ion or electron, where the distribution of particles or charge surrounding it is not uniform under the influence of light exposure. For illustration, consider an optochemical micro/nanorobot made of TiO_2_/Au in which the H^+^ rate for the generation of hydrogen on the Au side is comparatively lower than the TiO_2_ side in the generation of oxygen. This gradient of H^+^ concentration occurs due to the higher H^+^ concentration rate on the Au side than on the TiO_2_ side, resulting in the induction of the electric field for their propulsion along the direction of the TiO_2_ side. Self-electrophoresis is a process in which the self-generation electric field is established because of the gradient formation of asymmetric ion distribution around the particle, resulting in the transportation of micro/nano charged particles. For instance, generating H^+^ gradients in the photocatalytic reactions forms a static *E* that causes microrobot motions, and the surface charge on it derives its direction of motion. The propulsion of a Pt/Au rod-based microrobot was demonstrated with the self-electrophoresis technique [93], in which light exposure oxidizes H_2_O_2_ to generate electrons onto the Pt side and protons into the solution as shown in Figure 16(A){i}. Also Figure 16(A){ii} shows the feasibility of cargo loading on Pt/Au rod-based microrobot for the application like drug delivery system. Further, the generated electron and proton are transferred to the Au side and consumed with the reduction of H_2_O_2_. It ensues an ion flux that establishes the electric field to propel the microrobot along the Pt side. Further, Pt/Au structures are designed in different shapes that can be driven under light to simulate the rotation of microgears. Additionally, a micropump was designed by merging Au and Ag plates for the particle pumping that is accomplished with light to decompose the H_2_O_2_ solution. This decomposition creates a weak electric field that moves particles in response to the surface charge of the micropump. Like the Au/H_2_O_2_ microrobot, a rod-shaped optochemical microrobot was developed using Au/ZnO material, as shown in Figure 16(B){i} [129]. The asymmetric distribution of protons in the H_2_O_2_ photocatalytic reaction induces an electric field in the presence of UV light, causing self-electrophoresis mobility. Since the resultant electric field is perpendicular to the longitudinal axis of the rod, it generates the torque needed to rotate the rod. Also, it can change their ballistic motion to circular motion by increasing the H_2_O_2_ concentration. Moreover, Figure 16(B){ii} shows that the microrobot can switch from dominating rotation motion to linear motion with an alteration of the incidence angle of light. With the advantages of translation and rotation motion, microrobots can be used to pick up, transport, and release objects. Subsequently, the Au/TiO_2_–based microwires shown in Figure 16(C){i–iii} generate a localized electric field when exposed to low-intensity UV light [130]. It allows the microwire to propel itself through the mutual interactions of photoelectricity and the optochemical decomposition of water. These microwires can be precisely controlled to reach the target neuronal cells. Then, as depicted in Figure 16(C){iv}, they are electrically triggered by locally generated electric fields to open their calcium channels. Under low-intensity UV light, the figure shows that more than half of the neuron cells can survive, indicating tolerable biocompatibility. This research shows a novel technique to control neurological activities and bioelectrical signal transmission.

The major drawback of the UV light–driven optochemical microrobots in the application is the necessity of a solution like binary solution or H_2_O_2_. So, the traditional surface, i.e., inert oxide, of Janus microrobots was replaced with a noble metal, such as Au/TiO_2_, that can be propelled in pure water by UV light. As electrons flow from the TiO_2_ conductive band to the surface of Au, Au encourages the separation of the resulting charge pairs. This lowers the electron and hole recombination rates participating in the chemical reaction, boosting the overall reaction rate. Since, UV light can harm living organisms, the above-mentioned optochemical microrobots developed with UV-sensitive materials are unsuitable for many biological applications. To address this flaw, visible or NIR lights are utilized to power the optochemical microrobots. However, in order to drive, the optochemical microrobot needs sufficient temperature gradients that would make it possible with high intensities of visible or NIR light, but they cause thermal damage in the living tissue. Furthermore, researchers also mitigate above-mentioned issue by employing the use of narrow-band gap semiconductors or sensitized wide-band semiconductors in the microrobot design, which can be driven by visible and IR light. Recently, semiconductor material with a narrow band gap $\left(E_{g}<3.0\;\text{eV}\right)$ has been widely used as a photocatalytic material in developing microrobots because it can be easily powered by visible or NIR light. For example, silicon material $\left(E_{g}=1.12\;\text{eV}\right)$ absorbs infrared light, while $\text{CoO}\left(E_{g}=2.8\;\text{eV}\right)$ and $\text{BiOI}\left(E_{g}=1.77\;eV\right)$ absorb visible light from solar energy.

Microrobots made of single Si nanowires, as shown in Figure 16(D){i}, powered by visible or NIR light can harness light energy for propulsion through the self-electrophoresis mechanism [131]. Here, *n* ^+^-Si and *p*-Si surfaces undergo redox reactions that produce the imbalanced ions OH and H^+^, respectively, under the influence of light, which results in electric fields causing the movement of nanowires. The results of nanowire trajectories are shown in Figure 16(D){ii}, different because of their different end faces resulting change in electric field profiles as shown in Figure 16(D){iii–iv}. This research infers a novel method for the development of nanowire motors that could be propelled with low-intensity visible or NIR light. The visible light–actuated microrobot is made of BiOI material due to its unique photocatalytic properties shown in Figure 16(E), where its propulsion in pure water is due to a photocatalytic reaction without the need for chemical fuel [132]. Furthermore, it also demonstrated the impact of various coatings on the microrobot speed in order to validate the self-electrophoretic mechanism of this device. Similarly, Figure 16(F) presents a visible light–driven CoO/TiO_2_ microrobot that can propel itself into pure water by decomposing it into oxygen and its free radicals [94]. This microrobot is also magnetic in nature and offers the capability to control its steering by employing an external magnetic field. This microrobot has good biocompatibility, and due to the hybrid actuation, it has potential applications in disease treatment and drug delivery.

Therefore, it inferred that the optochemical microrobot can do many different things, such as turnover, rotate, and group together under the influence of light. It is also very easy to control. Hence, it offers great benefits in micro–nano processing, environmental management, and biological healthcare.

#### 4.2.3 Bubble jet techniques

**Figure 17:**
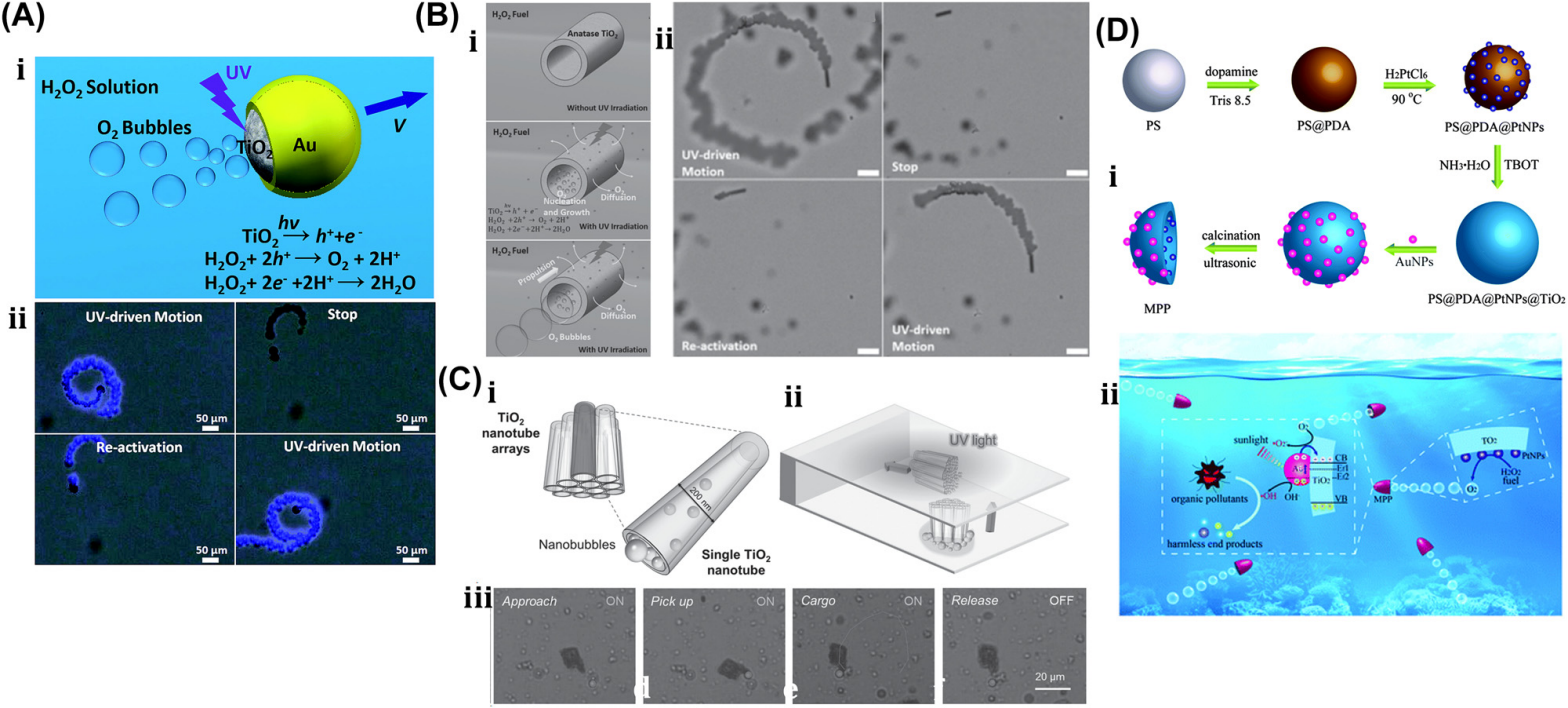
Bubble jet techniques. Bubble jet techniques. (A) (i) Schematic of amorphous TiO_2_/Au Janus micromotor in H_2_O_2_ solution under UV light. (ii) TiO_2_/Au Janus micromotor motion trajectories under the presence or absence of UV light. Reproduced with copyright permission from [134]. (B) (i) Mechanism of TiO_2_ tubular micromotor under UV light. (ii) UV light impact on micromotor motion. Reproduced with copyright permission from [135]. (C) (i) Microarray of TiO_2_ nanotubes. (ii) Lateral and transverse motion of TiO_2_ nanotubes microarray. (iii) UV light assisted TiO_2_ nanotube microarray for the transportation and cargo loading. Reproduced with copyright permission from [136]. (D) Gold nanoparticles and TiO_2_ half shell microrobot for wastewater treatment in anaerobic conditions. Reproduced with copyright permission from [137].

The optochemical µn-Bots based on a bubble jet is primarily propelled by bubble recoil, which is coined with a gradient mechanism induced by an asymmetric distribution of bubble concentration due to a photochemical reaction. Many optochemical µn-Bots motions are achieved with the bubbled recoil, but it has the issue that the gas generated through the photochemical reaction typically diffuses around it rather than producing a significant number of bubbles. To address this issue, the worldwide scientific community has been doing ongoing studies. The self-propulsion of opto-chemical microrobots made of silica-Pt material has been demonstrated with the bubble propulsion model established through the catalytic decomposition of H_2_O_2_, resulting in a change in momentum as O_2_ bubbles detach from the catalytic surface as shown in Figure 17(A) [133]. It reveals that the propulsive force is influenced by factors such as the solution’s surface tension, H_2_O_2_ concentration, and the rate at which bubbles detach. Also, it emphasizes the advantage of microrobots that restrict gas diffusion to accumulate gas effectively for bubble formation before dispersion.

The first photoexcited-induced bubble-driven microrobot was demonstrated through UV light exposed to the TiO_2_/Au microrobot, which catalyzed H_2_O_2_ to decompose and generate O_2_ bubbles to propel the microrobots as shown in Figure 17(A){i}. The microrobot speed and distance can be controlled or influenced by the strength of the UV intensity and also by their presence or absence as demonstrated in Figure 17(A){ii} [134]. However, the evaluation of the free energy formation (Δ*G*) of the bubble embryo with radius *R* on the tubular convex and concave surfaces revealed that the critical free energy of the concave surface is lower than that of the convex surface in TiO_2_ tubular microrobots [135]. The above studies reveal the research effort in recent years regarding light stimulation in the driving mechanism of the optochemical microrobot oriented by bubble recoil. The bubble-driven microrobots reported earlier mainly relied on the photocatalytic decomposition of H_2_O_2_ by precious metals as photocatalysts. However, TiO_2_ has gained much popularity in the development of bubble-driven microrobots because of its low cost, high stability, and excellent photocatalytic activity. A photoactivated bubble-driven tube microrobot was demonstrated, which is made of metal oxide in which TiO_2_ catalyzes H_2_O_2_ in UV light exposure and breaks it down to form oxygen bubbles, as shown in Figure 17(B){i}, which causes microrobot propulsion [135]. Figure 17(B){ii} shows that the speed and mobility state of the microrobot can be remotely controlled with the intensity and presence of UV light and also have a faster response. Moreover, the light-driven microrobot made from TiO_2_ nanotube microarrays presented as shown in Figure 17(C){i}, where the TiO_2_ nanotube microarray in the H_2_O_2_ is exposed to UV light, exhibits two distinct motion modes. Also, both motion modes are driven through oxygen bubbles catalyzed by H_2_O_2_ in the presence of UV light. The horizontally aligned nanotube microarray with the plate has horizontal motion, and the perpendicularly aligned nanotube microarray with the plate has vertically downward or upward motion, as shown in Figure 17(C){ii} [136]. Figure 17(C){iii} demonstrates the trials of microrobots used for cargo transport in fluid environments. The light-driven microrobot made from the composition of gold nanoparticles and TiO_2_ half shell demonstrated for wastewater treatment in anaerobic conditions as shown in Figure 17(D). Here, the H_2_O_2_ is catalyzed by light exposure to decompose gradually and produce the bubbled oxygen that can not only drive the microrobot but also enable the photochemical reactions for the effective degradation of a wide range of organic contaminants [137]. The optochemical actuation in WO_3_-coated Galinstan LMD has also been demonstrated [30]. The LMDs propel in the H_2_O_2_ solution with the moving UV light source. However, the H_2_O_2_ concentration, UV light intensity, and LMDs size significantly influenced the motion of liquid marble. Investigating the photochemically induced movement of marbles along arbitrary trajectories can expand this research. Additionally, the system can be improved by introducing a detector and feedback controller to regulate the speed of the moving beam in relation to the LMDs motion, ensuring that the light beam stays within the LMDs reach. Of course, the bubble jet microrobot gained popularity due to its powerful thrust and excellent propulsion speed in a liquid environment, which is independent of its ionic strength. It has a longer life cycle, which is a significant advantage. However, unpredictable trajectories, unwanted bubbles, and the need for toxic fuel are significant disadvantages that must be considered in the design and applications of microrobots. Also, tubular microrobots are faster and more efficient than spherical microrobots. On the other hand, its structure is too complex due to the multiple layers that necessitate the finicky and time-consuming methods in the manufacturing process, including layer-by-layer template electrodeposition and gas deposition multilayer coiling.

#### 4.2.4 Self-thermophoresis–induced optochemical µn-Bots

As mentioned in the previous section, optochemical microrobots are classified as self-electrophoretic, self-diffusion, and bubble jet. This section introduces the optochemical microrobot driven through the photothermal effect known as self-thermophoresis. In early 2007, Govorov et al. demonstrated the microrobot motions through the temperature gradient induced by light, where the swimming thermal force experienced by the microrobot can be calculated qualitatively with equation (1).

(1) $$F=-C\nabla T\left(r,t\right)$$

where *C* represents the calculated thermal conductivity and $T\left(r,t\right)$ represents the local temperature as a function of space, *r*, and time *t*.

$$C=\left(9\pi dp\eta 2k_{a}\right)/\left(2\rho Tk_{p}\right)$$

Here, *dp* is particle diameter, *η* is fluid viscosity, *k*_*a*_ is the thermal conductivity of the fluid, *ρ* is the fluid density, and *k*_*p*_ is the thermal conductivity of the particle. The equation makes it evident that the thermal conductivity is relatively dependent on the temperature gradient and particle diameter.

**Figure 18:**
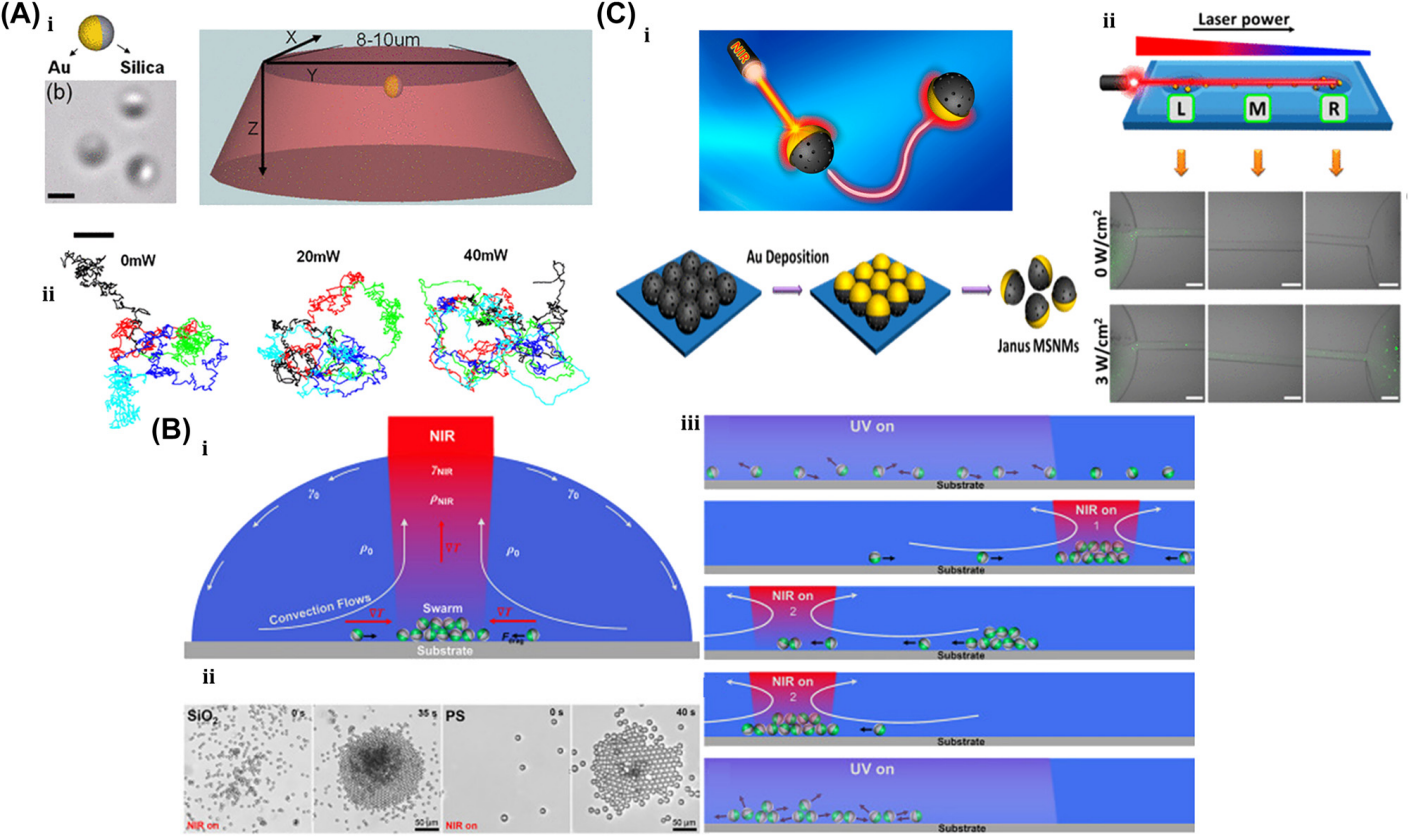
Self-thermophoresis–induced µn-Bots. (A) (i–ii) Schematic of Au/silica microrobot. (iii) Motion trajectory of Au/silica microrobot at different powers. Reproduced with copyright permission from [138]. (B) (i) NIR light–driven assembly of TiO_2_/Pt microrobots. (ii) Convective flow of TiO_2_/Pt microrobots at different mediums. (iii) Migration TiO_2_/Pt microrobots to the NIR point. Reproduced with copyright permission from [139]. (C) (i) Schematic and fabrication TiO_2_/Au micromotor. (ii) Cargo transportation of TiO_2_/Au micromotor under different power of NIR laser. Reproduced with copyright permission from [140].

The light-driven Au/Silica microrobots investigated can propel themselves with a temperature gradient induced by asymmetric irradiation or shape as shown in Figure 18(A){i–ii} [138]. After a lot of effort has been made in recent years to mimic biotic ecosystems, it will eventually be possible with light-driven microrobots that have been designed with the potential capabilities of material diversity, good controllability, and autonomous operation. However, in order to mimic the motion of biological communities, autothermal swimming techniques are the most often used in light-powered microrobots, where the surrounding fluid circulates with an asymmetrical distribution of temperature and causes movement in particles. Typically, an asymmetrical structure of two faces in particles is acquired by a thin, half-coated gold layer. The techniques to assemble TiO_2_/Pt microrobots in the colony shown in Figure 18(B){i} can be driven through the NIR light [139]. When NIR light is irradiated in a specific region, it induces convective flow due to the temperature gradient between the light-irradiated and unirradiated regions, resulting in the gathering of dispersed microrobots as Figure 18(B){ii} in response to convection resistance. Also, Figure 18(B){iii} shows that the microrobots can migrate to the specific location of the colony with the movement of the NIR point. Such studies show significant potential for cooperative cargo transportation with the microrobot group that can subsequently be utilized in the realm of intelligent transportation. The cargo transport with a TiO_2_/Au micromotor is demonstrated in Figure 18(C){i}, in which microrobot mobility is driven by a temperature gradient formed due to the opto-thermal effect in the Au half shell irradiated with an NIR laser [140]. Figure 18(C){ii} implies that smaller microrobots swam faster than larger microrobots in the same ambiance condition due to the effect of finite size on larger microrobots developing a smaller temperature gradient and causing a large resistance in the fluid. The figure shows microrobots offer a significant improvement in operability and can transport goods in a bio-friendly manner. Microrobots have the potential to be used in biomedical applications like drug delivery but also to ensure drug loading efficiency, which further necessitates their penetration enhancement. A microrobot driven by a self-heating mechanism was developed for anticancer drug transportation [141]. The microrobot is driven by the force of self-thermal conductivity generated by a laser-irradiated half-shell structure, which permeates the cell membrane with its thermo-mechanical forces. The light-irradiated microrobot aggressively pursues and effectively penetrates tumor cells and is able to control drug release by recognizing tumor cells, which can infect nearby tumor cells again after emerging from surface cells. So, it has good penetration, a high utilization rate, and can help lessen drug side effects. The laser irradiation yields the convective flow in water from a temperature gradient between the laser-exposed and unexposed regions, and the result of the convection force migrates micromotors in the group to its center. Micromotor migration and clustering can be immediately and reversibly controlled with the modulation of the light intensity, which further controls the size and speed of their clusters.

Furthermore, this technique could be easily operable, exhibit admirably precise spatiotemporal control, and be capable of operating several micromotors at the same time, which broadens its prospect of application in chemical sensing, intelligent transportation, and many other domains.

### 4.3 Photothermal-induced soft µn-Bots

**Figure 19:**
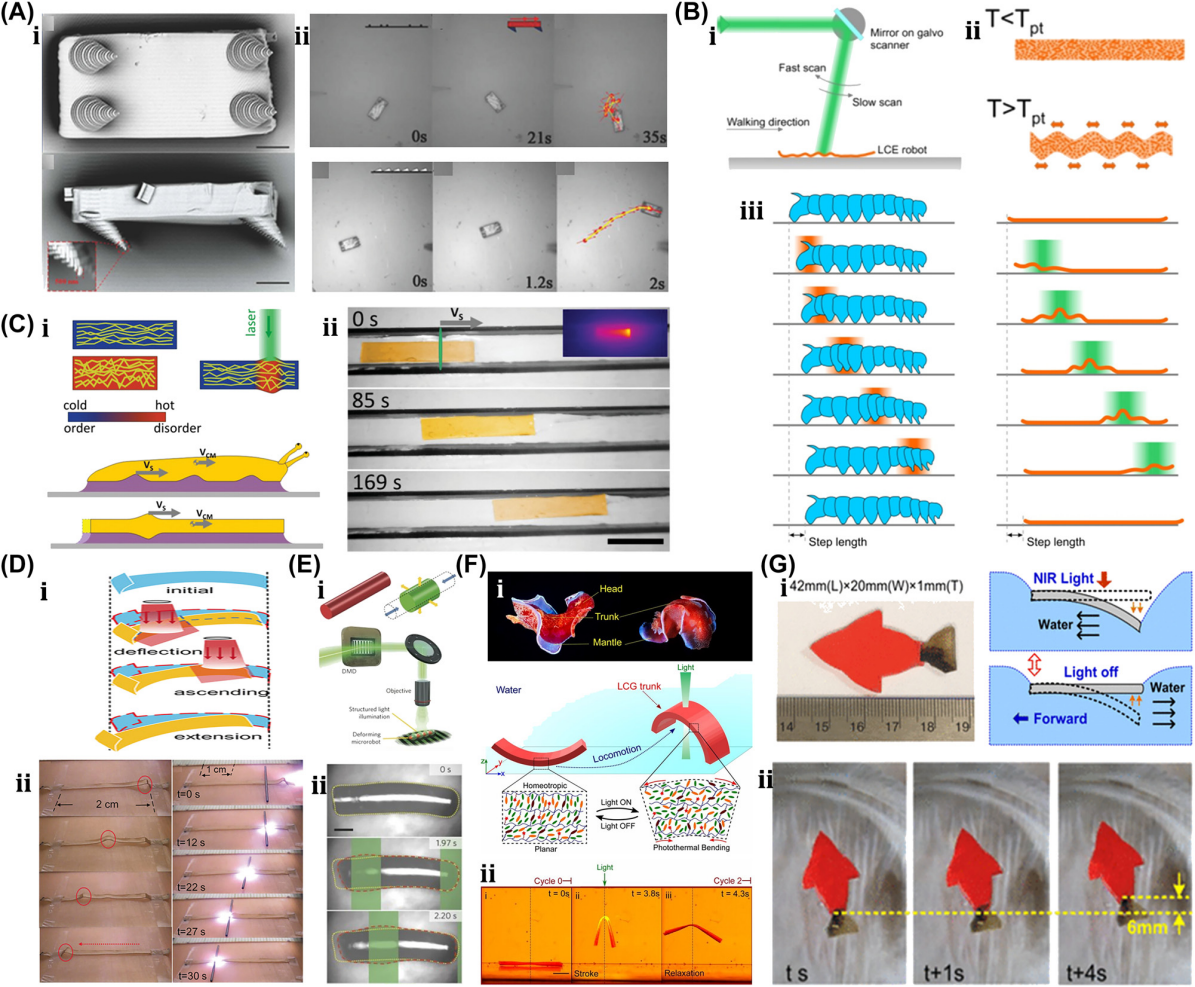
Photothermal-induced LCP soft microrobot. (A) (i) Top and side view of microtractor. (ii) Crawling locomotion on PI-coated glass and clean glass. Reproduced with copyright permission from [95]. (B) (i) Experimental setup of LCE-based caterpillar. (ii) Principle mechanisms. (iii) Crawling motion of caterpillar by moving laser shaded by green color. Reproduced with copyright permission from [7]. (C) (i) Principle mechanism of snail crawling. (ii) Crawling demonstration of artificial soft microrobots. Reproduced with copyright permission from [96]. (D) (i) Principle mechanism of soft microrobots. (ii) Transportation of carbon rods. Reproduced with copyright permission from [97]. (E) (i) Principle mechanism of LCE bases cylindrical microrobot. (ii) Radial expansion and axial contraction under light illuminations. Reproduced with copyright permission from [8]. (F) (i) Principle mechanism of biomimicking of *H. sanguineus* underwater motion. (ii) Demonstration of periodic shape deformation. Reproduced with copyright permission from [98]. (G) (i) Artificial microswimmer and their working principle. (ii) Locomotion of soft microswimmer in response to NIR induced tail movement. Reproduced with copyright permission from [99].

**Figure 20:**
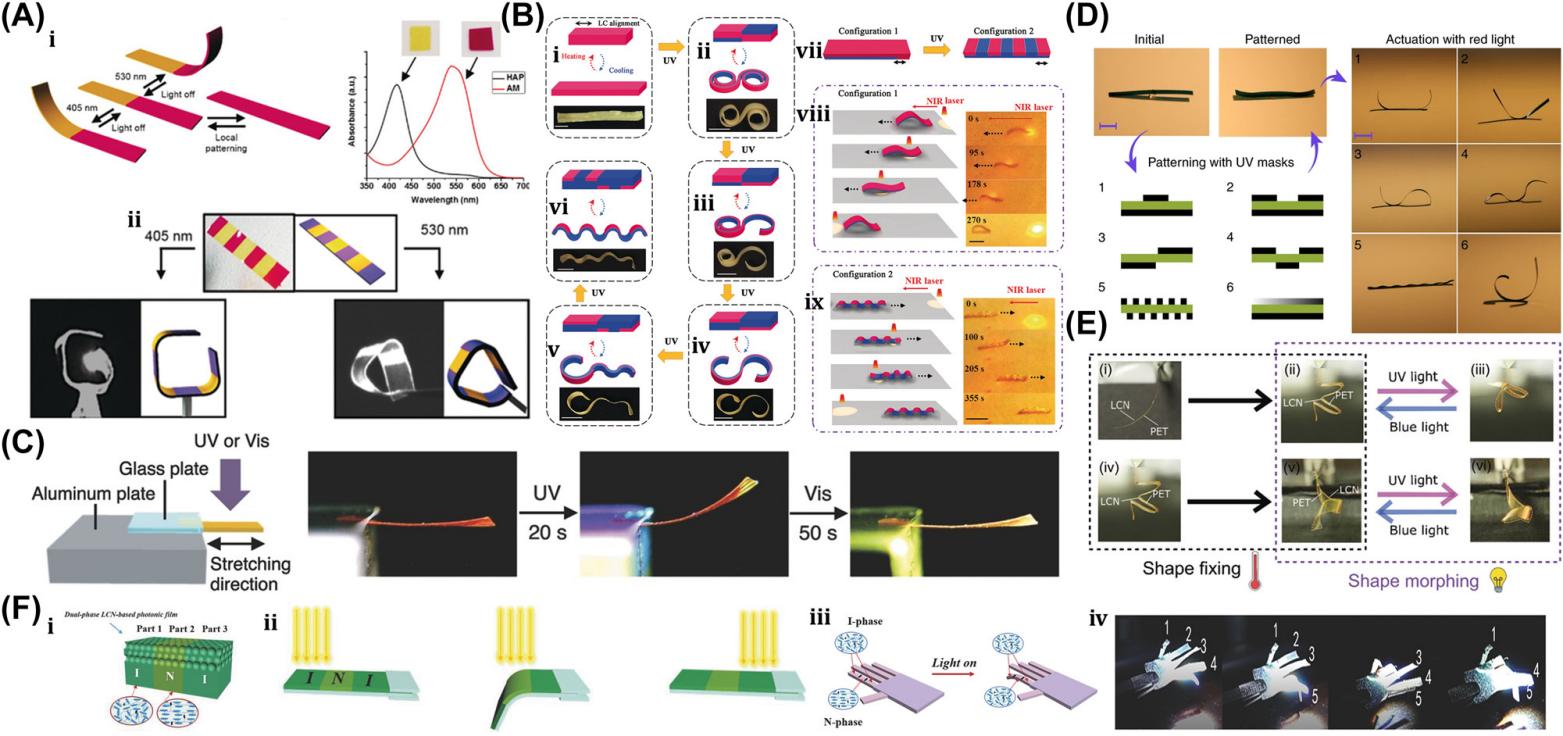
Photothermal-induced programmability in LCP. (A) (i) Light responsive programmable and rewritable LCP actuator. (ii) Spectrum of LCP film wavelength absorption pre- and postacid treatment. (iii) LCP actuator in response several wavelength light. Reproduced with copyright permission from [16]. (B) (i–vi) Microrobot delinking with light-controlled deformation includes elongation, roll up-roll down, roll up-bend down, S shape, bend up-wrinkle and all-wrinkle. (vii) Light walker-crawler transformation. (viii) Locomotion of arch shape actuator. (ix) Locomotion of wrinkle shape actuator. Reproduced with copyright permission from [17]. (C) LCE film deformation under UV and visible lights. Reproduced with copyright permission from [9]. (D) Demonstration of six distinct reversible morphologies exhibited by a single soft microrobot under similar exposure of red light. Reproduced with copyright permission from [18]. (E) Distinct optical drive with the two individual LCN bands geometries. Reproduced with copyright permission from [19]. (F) (i) Three-segment LCN-based in the N–I–N phase configuration. (ii) N-phase area exhibited fast bending under the exposure of visible light. (iii) Light-driven bionic hand with the dual-phase LC photonic film actuator. (iv) Demonstrations of complex motion of bionic hand. Reproduced with copyright permission from [20].

**Figure 21:**
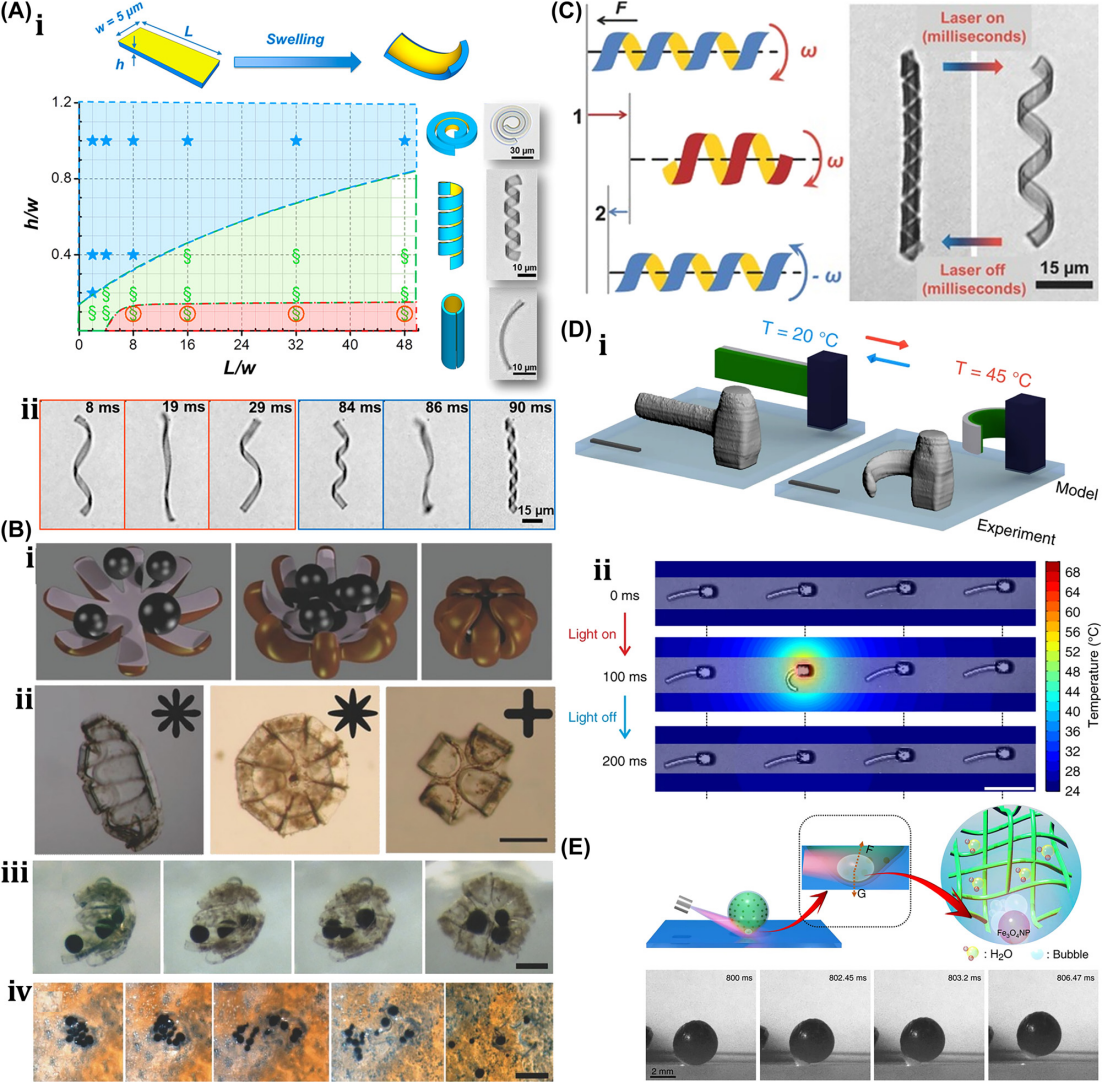
Photothermal-induced hydrogel microrobots. (A) (i) Double layer hydrogel band in various shape. (ii) Shape unwinding and reversal. Reproduced with copyright permission from [21]. (B) (i) Schematic of hydrogel based self-folding microrobot for drug delivery. (ii) Various shape of hydrogel-based self-folding microrobot. (iii) Grabbing and releasing of magnetic alginate particles. Reproduced with copyright permission from [22]. (C) Hydrogel-based microswimmer motion in the laser light exposure. Reproduced with copyright permission from [24]. (D) (i) Thermo-responsive behavior of hydrogel made 3D heterostructure microbeam. (ii) Actuation of hydrogel heterostructure microbeam under laser light exposure. Reproduced with copyright permission from [25]. (E) Excitation of hydrogel microvalve in response to laser light exposure. Reproduced with copyright permission from [27].

The soft material is most prominent in the design of microrobots because it could imitate the functional features of living tissues. Light-triggered soft microrobots utilize light as fuel energy and result in their body movement to carry out mechanical tasks. Polymer-like LCP and hydrogel are the two most often used in the fabrication of light-triggered soft microrobots, and their examples are shown in Figures 19–21. Fabrication techniques such as lithography and laser direct writing (LDW) are the most popular and well-established in such soft microrobot design. The fabrication of a soft microrobot begins with a substrate made of glass or metal coated with homogenous material that is placed under near-collimated UV light exposure through the mask plate. Eventually, the UV-exposed materials become hardened while the mask-obscured material is softened, which results in the development of the microrobot with the desired shape. Furthermore, the photoresist SU-8 is most widely used to create SU-8 molds in the making of PDMS models using the lithography replication process. The steps for the PDMS models using lithography replication are as follows: First, the appropriate ratio of curing agent and PDMS is mixed together, and then the mixture is put into a SU-8 mold and heated for curing. After cooling down the mold and PDMS, the PDMS is peeled off to obtain the necessary mold. Again, the PDMS mold is filled with the ingredients or materials of the microrobot and cured with UV light. Finally, the mold needs to be removed to get a microrobot with the desired shape.

#### 4.3.1 Photothermal-induced LCPs soft microrobot

LCP has gained incredible popularity as a soft material because of its superior thermal deformation performance. The nematic liquid crystal morphology is induced by the synthesis of cross-linking polymerization of photoinitiators, cross-linking agents, and anisotropic nematic liquid crystal molecules (LCM), whose shape changes spontaneously and reversibly with heating. The photothermal materials included in LCP act like an optical switch that can absorb visible, NIR, and IR light and transform their photon energy into heat energy. Typically, when the photothermal material’s temperature increases in response to light, the LCP phase translates into the isotropic phase, resulting in reversible deformation. The energy transformation caused by photothermal materials served as the foundation for the photothermally actuated soft microrobot. Also, visible, near-infrared, and infrared (NIR and IR) light can penetrate biological tissue very well. It has special benefits and can be used in artificial muscles and intelligent bionics. Researchers were inspired by numerous microorganisms, like terrestrial, aquatic, and amphibian ones, in order to develop soft microrobots, land microcrawlers, and microswimmers to understand their functionality and mimic the motions that microorganisms do. The majority of such microrobots have photothermal effect–based actuation mechanisms. These microrobots convert the absorbed light into heat, causing the polymer network to transition from order to disorder, activating local contraction and subsequently driving the microrobot’s motion. LCP material was utilized as an artificial muscle to develop the light-driven microwalker shown in Figure 19(A){i}, which has a torso with four cone-shaped legs [95]. Figure 19(A){ii} shows the locomotion of the microwalker on the glass surface coated with PI, in which it can randomly walk and rotate around one fixed leg in response to laser irradiation. Also, on a clean glass surface, it can be able to self-orient. Further, the performance of microwalker locomotion can be significantly enhanced with the optimization of their body shape and leg inclination angle.

Several microorganisms, like earthworms, caterpillars, snails, and other mollusks, can achieve locomotion through the deformation of their body shape. For example, a caterpillar has eight pairs of delicate legs on its very soft, cylindrical-shaped body, and its locomotion is like crawling. Figure 19(B){iii} shows each cycle involved in the caterpillar’s crawling motion, where it lifts its end legs from the ground and moves forward to make new contact with the ground. Then, its abdomen’s legs lift in order from back to front and move a step forward to create a wavy movement from tail to head. The LCP-based soft microrobot was developed to mimic the caterpillar’s gait via local bending and contraction through laser irradiation as shown in Figure 19(B){i} [7]. Figure 19(B){ii} shows that the temperature of the local region of the microrobot liquid crystal elastic film increases with exposure to laser light. If the laser light–induced temperature of the liquid crystal elastic film is greater than its phase transition temperature, the molecular arrangement becomes an order to disorder and results in the expansion and contraction of a thin film that further establishes the microrobot locomotion. After 3 years of research, a crawler was demonstrated, which mimics the locomotion of terrestrial gastropods like snails. Such crawlers are designed by integrating the adhesive qualities of synthetic mucus with the laser-induced deformation of a soft elastic body [96]. When the crawler keeps on an artificial mucus-thin layer and is exposed to laser light, its local body heats up with light absorption. As a result, the crawler’s body contracts with the reduction in molecular order, and it crawls along the laser direction at the stipulated speed. Figure 19(C){i} shows how the artificial mucus layer interacts with the crawler’s volume shrinkage and propels the microrobot forward. An LCP-based crawler similar to a worm that fixed its ends on the horizontal substrate developed. When the crawler is exposed to laser light, the area it scans warms up and generates a moveable bulge, as shown in Figure 19(D){i} [97]. Also, the crawler has the ability to perform straight-line motion, direction turns, and slope climbing, which makes it useful in object transportation through laser. Figure 19(D){ii} shows the carbon rod’s transportation through the crawler using a laser.

Researchers and the scientific community have also mimicked aquatic invertebrates and achieved several kinds of underwater motion with the modification of molecular anisotropy and the photoresponsiveness of LCP materials. A cylindrical-shaped microrobot with the photoactive LCE demonstrated as shown in Figure 19(E){i–ii}, which is referred to as a microswimmer. It could expand radially and contract axially under uniform illumination [8]. Such microrobots propel themselves through periodic body deformations similar to those of biological microswimmers and do not need biological cells, force, or torque. As shown in Figure 19(F){i}, the mimicking of *H. sanguineus* underwater motion is presented using a photoresponsive liquid crystal [98]. Figure 19(F){ii} shows the simulation of the *H. sanguineus* central trunk made of liquid crystals that can be susceptible to deformation due to the photothermal effect, which only occurs when molecular arrangement in the liquid crystals undergoes ordered transitions to disordered transitions in light exposure. LCP film with a PDA coating is also used in the fabrication of a microswimmer because LCP has a thermal response, and the PDA coating is affected by photothermal. Since only the underside of the LCP film is coated with PDA, the artificial tail can only bend downward. LCP film is shown in Figure 19(G){i} shining by the NIR laser light, where the surface of the PDA layer instantly transforms light energy into heat energy that causes the tail to bend downward [99]. Also, Figure 19(G){ii} shows NIR light under the off condition, where the tail curve resumes and moves up by squeezing the fluid backward to generate thrust and forward the entire body to propel swimming.

#### 4.3.2 Photothermal-induced programmability in LCP-based soft microrobots

The liquid crystal in soft microrobots has gained potential interest in replicating microcrawlers, aquatic organisms, and muscle actuation, although it has some limitations. Typically, the traditional natural system involves variable driving modes and complicated geometries, whereas LCP material makes soft optomechanical robots constrained by initial geometries. The LCP films designed by most researchers persist in single-driving modes like bending that prevent the polymer from being recycled to achieve a different starting shape. So, researchers have initiated a significant effort to achieve the programmability of microrobot shapes and motions. An approach for making a soft microrobot out of a programmable and rewritable light-responsive liquid crystal made of azo-merocyanine (1-AM), which turns into hydroxy-azo-pyridine (1-HAP) when treated with acid, is presented, and their absorption spectra before and after acid treatment is shown in Figure 20(A){i} [16]. Figure 20(A){ii} shows different color lights utilized to trigger the folding microrobot, which is made of several dyes, and each dye has a distinct wavelength to absorb light. Since these acid patterns are reversible, film patterns can be wiped and re-edited to reuse soft microrobots.

Light can delink LCP photo actuators, reallocating cross-linked and non–cross-linked areas because anthracene dimers’ photocleavage can selectively delink cross-linked regions. The photocleavage of anthracene dimers, which could selectively delink cross-linked regions, makes it possible for the reallocation of the cross- and non–cross-linked regions in the LCP photo actuators. Here, a magenta, cross-linked region (called a driving region) could undergo reversible deformation in response to a light stimulus; however, a blue, non–cross-linked region (called a nondriving region) has no inverse change in its shape. The LCP photo actuators of several shapes shown in Figure 20(B){i–vi} could be rebuilt with the control of nondriving domain shape and depth [17]. Figure 20(B){viii–ix} demonstrates the transformation of LCP photo actuators walker to crawler, locomotion arch shape photoactuator, and wrinkle shaped photoactuator under the exposure of light.

An LCP made with azobenzene groups and dynamic covalent bonds ensues to rebuild the soft microrobots. Here, the LCP shape in a hot state may be modified by rearrangement of its network topology through an exchange reaction. Also, it shows the reconstruction behavior of liquid crystal films with photo-induced deformation. Figure 20(C) here infers that UV light-exposed films experience bending toward the light, and deformed films recover back to normal when exposed to visible light, which is similar to material bending behavior without the exchange of dynamic covalent bonds [9]. Moreover, depending on its initial shape, the reconstructed structure exhibits a different motion. Also, shape reconstruction in a photoresponsive soft actuator is possible by incorporating the dynamic covalent bond.

The light-responsive liquid crystals were presented in the development of the reconstruction of a soft microrobot, in which the photothermal effect is used for photo-induced deformation and photochemical effects accomplish shape programming. Figure 20(D) demonstrated the six types of reversible morphology constructed with the same microrobots in the same light conditions [18]. This key idea was used to realize an intelligent, gripping device that can hold and release an object with the control of light. This study presents a simple and effective method for the shape reconstruction of microrobots. However, using two different geometrically shaped LCP bands revealed a distinguishable optical driver, as shown in Figure 20(E) where an accordion-like band folds into an origami pattern while a spiral band unfolds [19]. Such techniques produce a multipurpose and simple photo responsive thermoplastic actuator that is mechanically robust and recyclable, ensuring soft microrobots’ recovery. Moreover, the thermo-mechanical element LCP, the structural color template Si opal, and the photothermal conversion dopant graphene oxide were utilized to develop a photoactivated dual-phase photo actuator [20] as shown in 20(F){i}. The dual-phase photo actuator has the features of complex shape changes, numerous driving modes, and selective drive, which can be obtained from the fabrication of nematic (N) and isotropic (I) states in the UV exposures. Figure 20(F){ii} shows that the N-phase region bent rapidly under visible light irradiation while the I-phase region became unaffected, resulting in different bending behaviors in films under visible light irradiation. Figure 20(F){iii–iv} shows a light-triggered bionic hand based on the principle of a dual-phase photo actuator that can mimic the motion of a biological hand finger in visible light.

#### 4.3.3 Photothermal-induced hydrogel microrobots

Hydrogels are very sensitive to environmental parameters that could detect a minimal change in the parameters, including pressure, temperature, humidity, pH, and light, leading to commensurate modifications in their chemical composition and physical structure. Such hydrogels can be commonly used in sensors, controllable release switches, and microrobots because their swelling behavior substantially depends on the surrounding environment. A gold thin-layer–coated hydrogel band made of N-isopropyl acrylamide polymer [21] was first presented, which becomes curled due to a temperature rise in the thin gold layer in the presence of infrared light irradiation as shown in Figure 21(A){i}. The experimental result shown in the figure demonstrates that the crimp in a double-layer band shape was dependent on its length and cross section. The experimental result shown in the figure demonstrates that the shape of the double-layer band with different lengths and cross sections was crimped differently. Moreover, Figure 21(A){ii} shows that the spiral could be unwound and reversed in about 90 ms. These experimental results demonstrate the dynamic deformation of hydrogel that can have potential applications in microfluidics, artificial microswimmers, and soft microrobots.

To enable the precise control of microrobots, numerous techniques have been investigated to control and direct the deformation of shape-changing materials. Figure 21(B){i–ii} shows the typical design of the microrobot, a “self-folding soft microgripper” using bilayer hydrogel that could be activated by the NIR laser light [22]. Figure 21(B){iii} shows the two-step lithography used to incorporate the alginate magnetic particles into the bilayer-folded hydrogel that could be liberated by activating the bilayer hydrogel with the NIR light. Scallop’s theorem states that a microrobot swims in a sequence of nonreciprocal motions that need low Reynolds numbers, and the body deformation must follow a path with a distinct space-time dependence on the forward and shape-recovery operations [23]. Moreover, the swimming behavior at the microscopic level is significantly inspired by the cilia beating and flagella rotation. In order to mimic cilia and flagella, a photosensitive hydrogel was utilized. They discovered that gold nanoparticle–doped hydrogel could jump (in ms) due to more than 20 °C of thermal heat in the exposure of NIR laser light [21].

The photocontrol microswimmer device was developed with the prior established theory that it can achieve rotation and progressive motion with body deformation [24]. It is made of pNIPAM hydrogel, in which gold nanorods are implanted. Figure 21(C) shows the twisting and bending control in the microswimmer caused by the rapid volume expansion of thin pNIPAM hydrogel when its implated gold nanorod is heated with laser light. However, the microswimmer returns to its normal shape once the laser light exposure is turned off. Subsequently, a photostimulation-based three-dimensional heterogeneous microvalve and microbeam structure was made with pNIPAM and N-methylene bisacrylamide hydrogel [25]. Figure 21(D) shows that when the heterogeneous microbeam structure is placed under laser light exposure, its local temperature increases, which activates it and then bends. Micron-sized crawlers are also designed that are made of gold nanoparticle–doped *p*-NIPAM hydrogel that shrinks locally due to its thermally responsive properties in the presence of laser irradiation [26]. As a result of this local shrinkage in the hydrogel, an asymmetrical friction change occurs between the substrate and the tractor. Such a crawler uses eccentric irradiation to create directional motion, enabling it to drive tiny cargo across a flat surface like a micromanipulator. The actuator of such a responsive hydrogel has potential applications, but its actuation must often be accomplished in water. The hydrogel microrobot powered by light in the air was presented, which utilizes hydrogel’s elasticity and bubbles induced by the photothermal effect to avail of the features of quick response, ultrafast mobility, and a high height jump as shown in Figure 21(E) [27]. It offers numerous applications in regulated drug delivery, sensors, and many more fields.

Moreover, an intelligent hydrogel is designed to take advantage of its features to change shape in response to a temperature corresponding to biological culture. For example, by inducing the equilibrium between hydrophobic and hydrophilic in the side chain group, a low critical solution temperature (LCST) hydrogel can be achieved. The deformation rate and direction that can be precisely controlled are significant advantages of hydrogel microrobots. As a disadvantage of hydrogel in microrobots, motion is greatly limited due to isotropic volume change, lack of mechanical sturdiness, and, most notably, water-dependent function. To mitigate the aforementioned problem of hydrogel, it can be integrated with other materials like PDMS to achieve more diverse deformations. However, additional research revealed that it could not circumvent the stratification issue in these double- or multilayer systems. Techniques like soft lithography are used to develop a thick form of pNIPAM hydrogel, which has a freely suspended skeleton of glassy resin fabricated through direct laser writing. Such a skeleton causes a richer 3D deformation as the hydrogel swells.

### 4.4 Photomechanical effect

**Figure 22:**
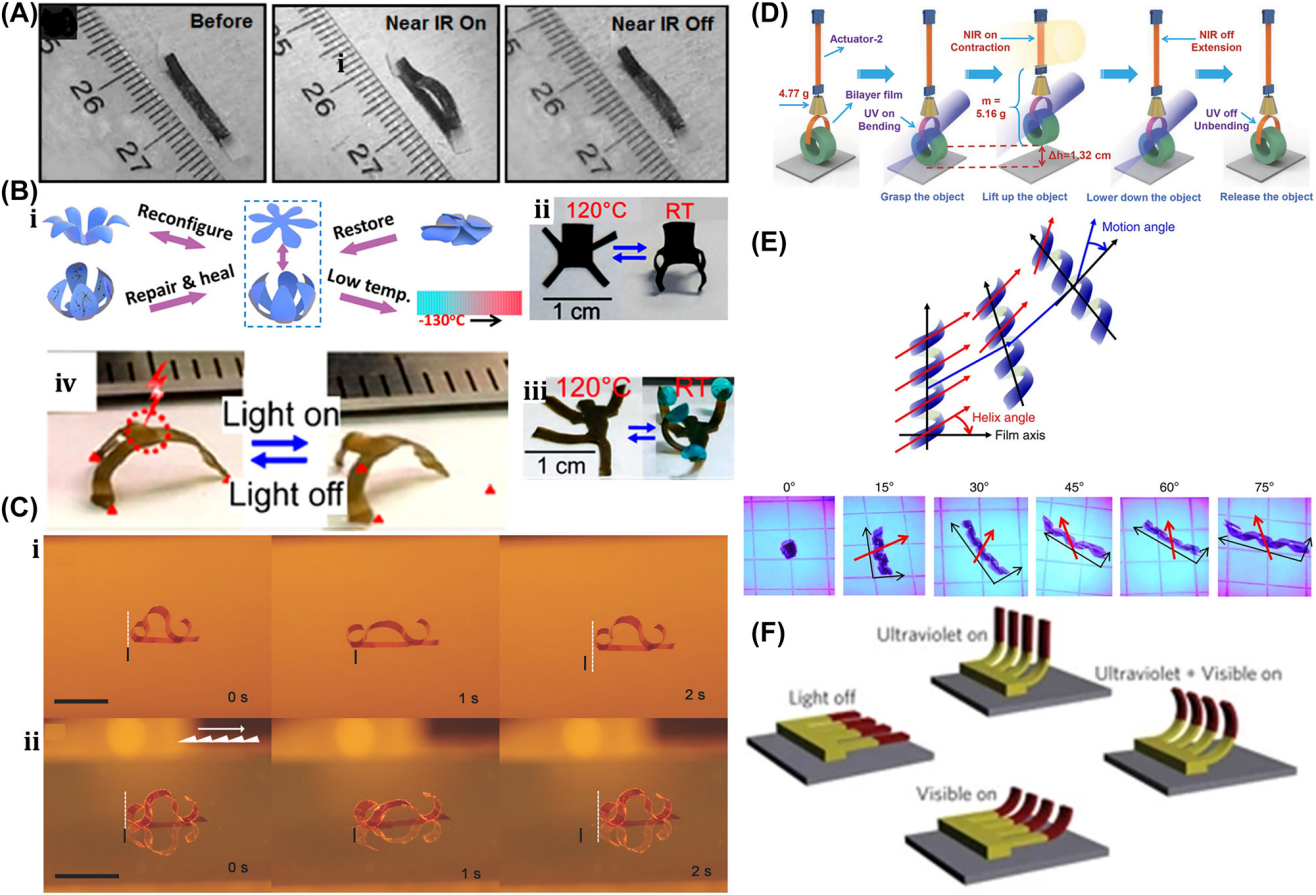
Photomechanical effect–induced soft µn-Bots. (A) Crawling of IR light–driven CNT/LCP thin film. Reproduced with copyright permission from [142]. (B) (i) Principle mechanism. (ii) LCP film with four legs shaped into chair. (iii) LCP shape as strong man lift up four ball in response to light. (iv) Tripod rotational locomotion with the IR light ON/OFF. Reproduced with copyright permission from [143]. (C) Light-driven motion of a liquid crystal film on the surface of (i) paper (ii) blaze grating. Reproduced with copyright permission from [144]. (D) Light-driven LCP-based crane motion for the tasks, such as grabbing, lifting, lowering, and releasing of a tubular object. Reproduced with copyright permission from [145]. (E) (i) Twisting motion of LCP film in visible light exposure (B) simulated trajectory of LCP film with the exposure of visible light. Reproduced with copyright permission from [147]. (F) Dual wavelength photo actuator bending motions. Reproduced with copyright permission from [148].

The photomechanical driving mechanism for soft µn-Bots is resulted from the photomechanical effect. To characterize the photomechanical effect, the photomechanical switch is like azobenzene doped into the LCP material, which causes its shape to change with the light absorption. Such photomechanical switches generate stress on the molecular network of LCP in exposure to light that breaks its molecular arrangement and results in a change in its macroscopic volume. The local structure volume change in CNT/LCP thin film was achieved using IR light irradiation. It results in a shape change that further acts as a driving force for the structure to crawl on the plane as shown in Figure 22(A) [142]. Also, IR light has been used to irradiate the thin films of CNT/LCP principle mechanism as shown in Figure 22(B){i}. It takes the shape of a chair when exposed to light, where the four legs of the chair films bend down while the back of the chair films is turned upward as depicted in Figure 22(B){ii} [143]. Then, it uses that to make the shape of a strong man shown in Figure 22(B){iii} capable of lifting an object. Further, Figure 22(B){iv} shows the locomotion of tripod with the periodic switch ON/OFF IR light. The simulated tracked locomotion of LCP microrobots was demonstrated by using the illumination of spatially uniform visible lights [144]. Figure 22(C){i-ii} shows that the microrobots can transform their shape and drive even in moderate visible light intensity and are capable of sensing the ground condition and motion on a range of substrates like paper surfaces, luminous gratings, and human skin. The light-driving capability of materials is enhanced by synthesizing the dynamic ALCN with doping of polymer-grafted gold nanorods, which could control actuation in response to NIR and UV light [145]. Also, Figure 22(D) shows the combined effect of photothermal-driven phase change and cis-photoisomerization of azobenzene intermediates in order to develop an LCP crane with a gripper and an artificial muscular arm that can be used to lift, grab, release, and descend the cargo. In recent years, the most prominent issue with soft mechanical microrobots has been continuous driving. It is worth noting that the LCP-made soft microrobot is easy to control, and its nature of oscillating motion can significantly improve the speed of motion. However, the continuous motion in such polymer actuators is often a consequence of the feedback loops that synchronize material properties and external input. Light-induced oscillation was first demonstrated in the liquid crystal–made cantilever beam in exposure to high-intensity light [97].

Additionally, it has been discovered that the offset induced in the nematic director into the principal axis of liquid crystal films results in shearing force [146]. Furthermore, the chirality across the sample thickness determines the material’s twist (left or right) direction. As shown in Figure 22(E), LCP films developed in a spiral shape made through azobenzene-functionalized LCP networks become a flat sheet into a spiral strip in visible light; conversely, they can travel longer distances with continuous light irradiation [147]. Figure 22(E) also shows that exposing the particular spiral portion to the light source during the motion can also activate it to continue the rolling motion. Photo-responsive actuators made of azo dyes distinct wavelength cross-linked LCP monomer ink printed in the shape of artificial cilia has been demonstrated [148], which is sensitive to different wavelength of light source includes UV and visible light. The differentiated bending motions of photo-responsive actuators under visible light, UV light, and both UV-visible light are demonstrated in Figure 22(F). Such photo actuator has potential applications in mixing and selective flow in microfluidic system. It is observed that the spatial temperature gradient of a liquid crystal microrobot operated by photothermal effects grows in the water because of substantial thermal dissipation. As a result, high-intensity light is required to power the microrobot through the photothermal effect. So, to address the aforementioned drawbacks, photomechanical effects are introduced to power up the soft microrobot. The natural ciliated microswimmer motion is mimicked and precisely approximated by the deformation-driven soft microrobot. Since the driving force results from light–matter interaction and there is negligible dependence on other properties, such as the ionic strength of the surrounding medium, no waste is generated during the drives. Not only do these features revolutionize this technique for potential candidates in environmental applications, but scalability depends on polymer processing technology, which should be conceivable in the downscaling of devices.

## 5 Optically driven soft µn-Bots: prototypes and applications

Light-driven microrobots offer the significant advantage of being able to operate autonomously, be remotely controlled via an ON/OFF switch, and have intensity control of light irradiation. Also, their photosensitivity and rapid response capability facilitate their ability to design paths and actively seek targets, making them widely employed in terrestrial and aerial robotics, cargo loading, drug delivery, environmental remediation, and bionics.

### 5.1 Terrestrial and aerial microrobot

**Figure 23:**
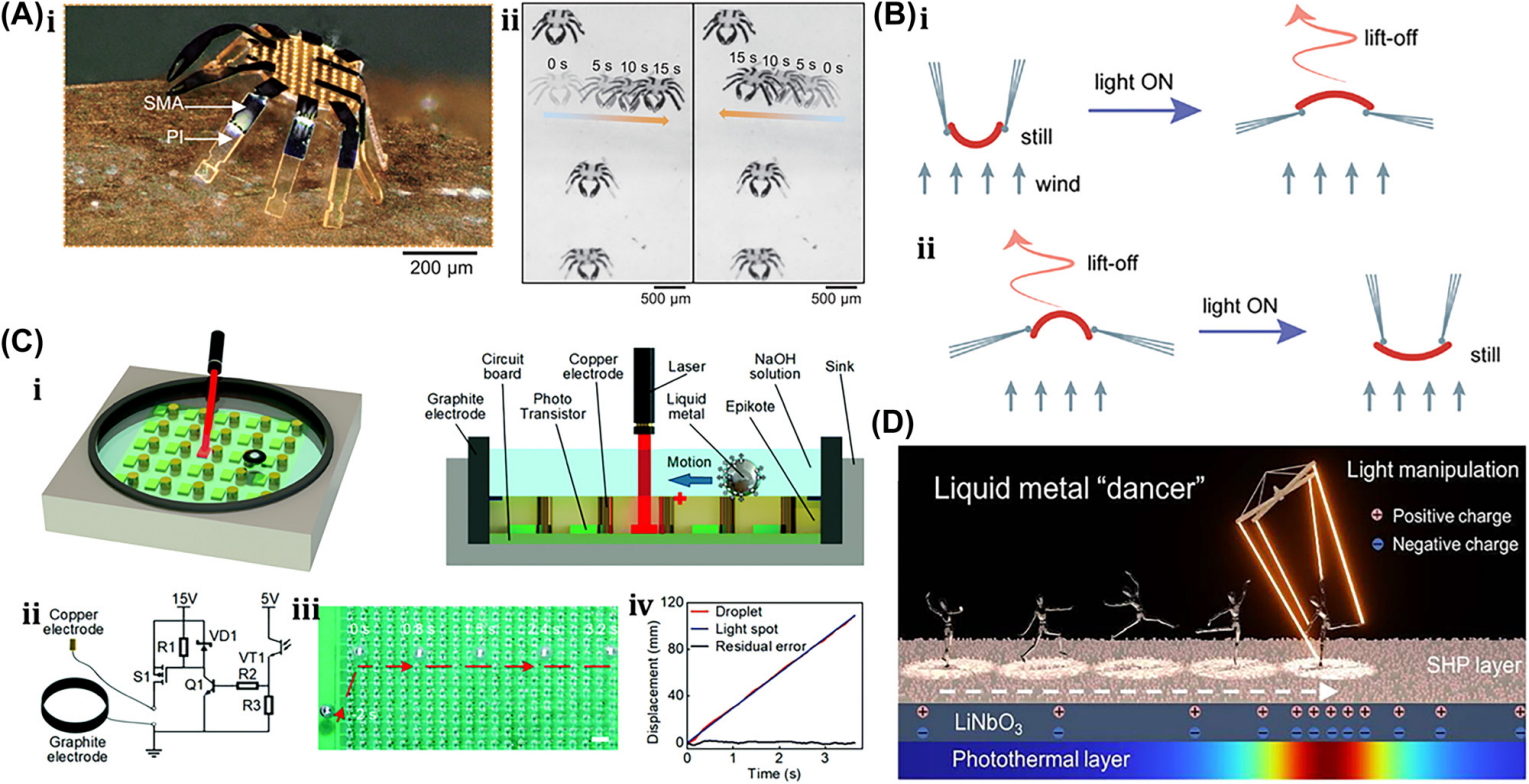
Terrestrial and aerial microrobot. (A) (i) Schematic SMA-PI carb microrobot. (ii) Locomotion of carb microrobot. Reproduced with copyright permission from [10]. (B) Dandelion seed inspired flying microrobot principle mechanism and locomotion. Reproduced with copyright permission from [11]. (C) (i) Schematic of 3D view and side view of fabricated system for the light-driven LM droplets. (ii) Principle mechanism. (iii) Light-driven LM droplet navigation. (iv) Displacement versus time plot for light-driven LM droplets. Reproduced with copyright permission from [100]. (D) Optical light-driven liquid metal droplet (LMD) “Dancer” in air environment. Reproduced with copyright permission from [28].

Recently, the robotic crab, the world’s smallest submillimeter-scale terrestrial robot locomotion, has been demonstrated through laser light. It has complex and heterogeneous 3D structures made with a multilayer material of PI and nitinol SMA. The fabrication of such a crab robot was developed by the processing of organic (PI and LCE) and inorganic material (Nitinol SMA) by a lithography process [10], as shown in Figure 23(A){i}. The deformation of the crab robot is enabled by laser-induced heating of SMA material that causes walking, crawling, walking, jumping, and turning with a speed of ∼0.44 body length/s demonstrated in Figure 23(A){ii}. Also, the pH sensor and photonics devices, such as retroreflectors, are equipped on the robot’s body for localization, control, tracking, and wireless communication.

The emergence of responsive polymers has expanded the possibilities for small, wirelessly operated soft robots. While existing synthetic robots can walk, swim, jump, and even disperse in the air, flying remains uncharted territory for these materials. Achieving the necessary performance, lightness, and aerodynamic efficiency in their actuators poses significant challenges. Recently, inspired by dandelion seed from nature, the laser light–controlled landing, liftoff, and wind-assisted dispersal of porous structures made of soft material have been demonstrated in Figure 23(B) [11]. The design exhibits biomimetics traits of dandelion seed, including light weight (1.2 mg) and excellent porosity (0.95), which make it detachable vortex ring production under constant wind flow. Such an artificial seed actuator was developed with LCE, which bristles open and close in response to visible light. The Marangoni effect–induced propulsion and manipulation of liquid metal (LM) droplets with controlled light have been demonstrated [100]. The 3D view and side view of the fabricated device are shown in Figure 23(C){i}. The LM droplet propulsion can be controlled by the resulting Marangoni forces due to the electrical current induced in the electrolyte with the selective activation of the phototransistor through the IR lights shown in Figure 23(C){ii}. It also demonstrates the various capabilities, including designated path motion, concurrent motion, and arbitrary motion for the single or multiple LM. Also, the splitting and merging of multiple LM droplets demonstrated by same setup. The light-driven navigation of LM droplet and displacement versus time plot shown in Figure 23(C){iii–iv}, respectively. Such light-controlled LM droplet propulsion can be utilized in field-programmable microrobots and micromachining. Recently, researchers have conducted pioneering research on an optical light–controlled LMD-based “dancer” in an air environment, drawing inspiration from the popular science fiction film “Terminator” as shown in Figure 23(D) [28]. This research could potentially contribute to the development of soft robots. The LMD propulsion in the air environment can be achieved with NIR light on the photo-responsive pyroelectric superhydrophobic (PPS) surface made of lithium niobate and a photothermal layer. Here, NIR light irradiation on the PPS surface generates free charges that decrease marble adhesion and contact angle, leading to dielectrophoretic and electrostatic forces on the LMD. The dynamic manipulation of LMD on the PPS surface, such as directional motion, climbing motion, obstacle avoidance, maze crossing, etc., was demonstrated through the guidance of NIR laser light irradiation. Additionally, it can serve as a demonstration tool for remotely operating traffic light circuits and intelligent welding circuits.

### 5.2 Object grabbing and transportation

**Figure 24:**
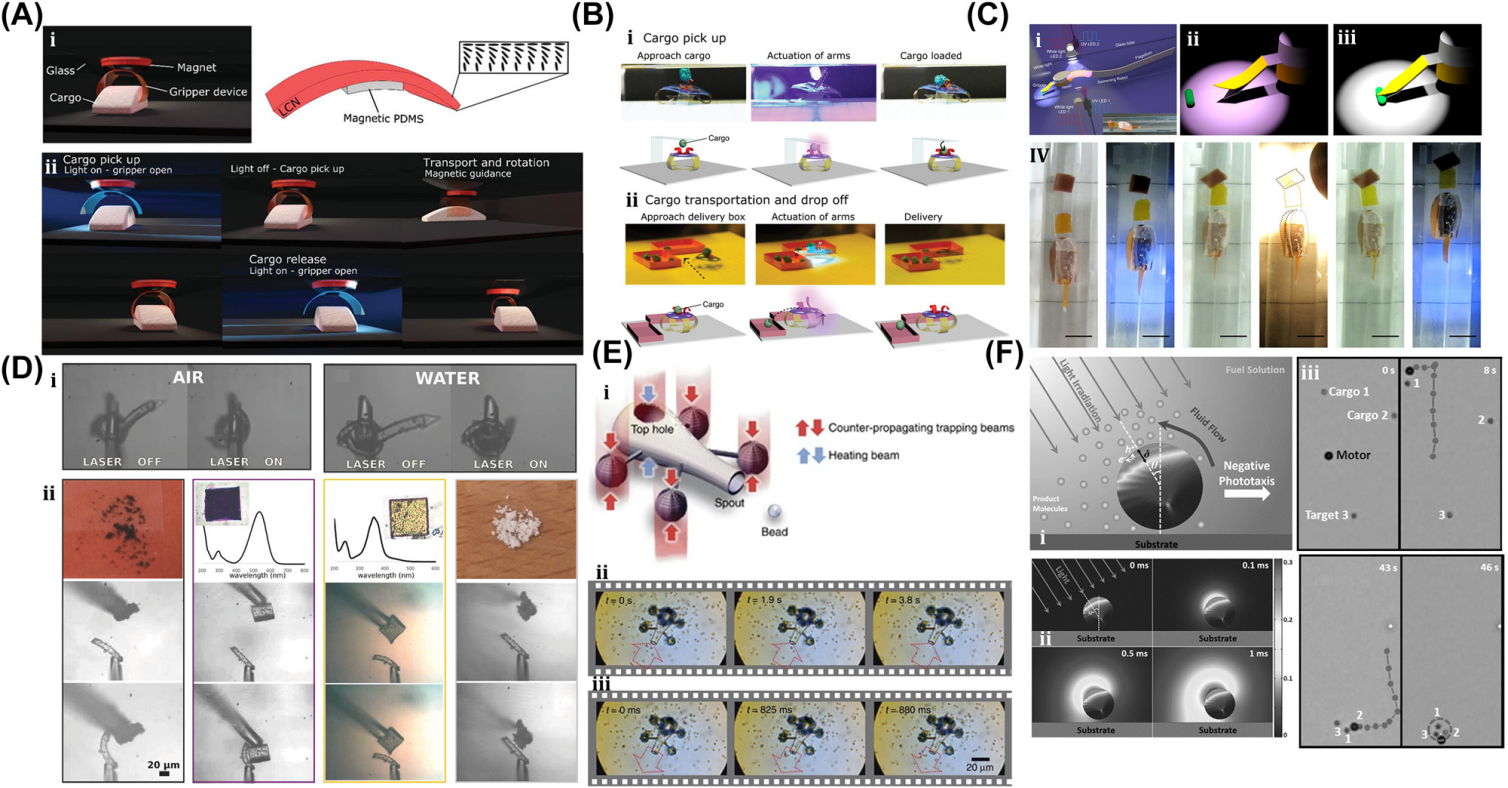
Object grabbing and transportation. (A) (i) Soft microgripper and its fabricated schematic. (ii) Magnetic field–driven transportation and light-driven cargo pickup and release. Reproduced with copyright permission from [168]. (B) (i) Light-driven cargo pickup demonstration. (iii) Light-driven cargo transportation and drop off demonstration. Reproduced with copyright permission from [169]. (C) (i) Light-driven swimming mechanism of microrobots. (ii–iii) Cargo capturing operation. (iv) Loading and unloading operation. Reproduced with copyright permission from [170]. (D) (i) Microcosmic hand in air and water. (ii) Microcosmic hand actuation for different objects. Reproduced with copyright permission from [171]. (E) (i) Light beam–driven micropump. (ii) Polystyrene bead capturing with micropumps. (iii) Polystyrene bead transportation and drop off through micropumps. Reproduced with copyright permission from [172]. (F) (i) TiO_2_ micromotor propulsion mechanism. (iii) TiO_2_ micromotor in cargo pickup, transportation, and drop off. Reproduced with copyright permission from [173].

Manipulating objects on a microscopic scale, like transporting and capturing them, is still complex yet necessary. The light-driven microrobot outperforms conventional electric field–controlled microrobots in the aspects of straightforward assembly, machining performance, and ease of control. Additionally, the micron-sized hand can be further reduced down to a nanometer scale and used in object transportation even in confined surroundings, such as microfluidic devices. A remotely controlled soft microgripper has been developed with integrated optical and magnetic responses shown in Figure 24(A) that can load, transport, rotate, and release cargo [168]. It is made by integrating the LCP thin layer with a photoresponsive azobenzene dye and a PDMS layer with magnetic iron powder shown in Figure 24(A){i}. Figure 24(A){ii} shows the cargo being captured using a microgripper with the control of light and further transported to the target location with the guidance of a magnetic field, finally then cargo is released via lighting. However, the above-mentioned remotely controlled microrobot driven by magnetic actuation could be used in the walking, handling, grabbing, and releasing of objects, but it cannot be accomplished solely through the actuator. So, a microrobot has been designed that is exclusively driven by blue light and has four LCP legs with yellow light switches, two LCP arms, and one claw with red light switches. It was observed that the LCP arm with red light switches has more absorption of blue light than the LCP leg with yellow light switches, resulting in more deformation. Furthermore, microrobot motions like bending and walking could be achieved in the LCP leg with an alteration in the light irradiation position on it. As the LCP arm meets the dangling cargo, it drops into the LCP claw, and then the LCP claw is closed with the removal of light in order to hold the cargo, as shown in Figure 24(B){i} [169]. Also, Figure Figure 24(B){ii} shows that the LCP microrobot leg forces it to bend its leg under the exposure of low light and form a slope to release the cargo. A light-actuated microrobot made of UV-light–sensitive LCP films has been developed and is capable of loading and unloading goods in a surrounding liquid environment. The microrobot is propelled to swim toward the target goods with a periodic flash of UV light, and its grippers are opened up by the continuous UV radiation. Figure 24(C){i-iii} demonstrates the microrobot loading functionality, in which the microrobot first reaches the point of loading where its gripper is closed to perform goods loading via the shining white light [170]. Also, Figure 24(C){iv} demonstrate the goods being transported by microrobot to the specified location and finally unloading them using UV radiation. Based on the above research, a soft microrobot has been developed with the capability of automatically recognizing objects of grayscale and different colors. Also, it can be operable in both air and water, as shown in Figure 24(D){i} [171]. Here, the object is captured via the microcosmic hand closure, which is triggered by a locally produced heat effect only when the absorption spectrum of the target object matches the light wavelength. Figure 24(D){ii} shows that the actuation in the microcosmic hand in the presence of green laser irradiation can only be observed when it is close to the carbon nanoparticles and purple polymer blocks, but when it is close to the yellow polymer block and TiO_2_ nanoparticles, there is no actuation.

Aside from the microcosmic hand, numerous varieties of light-driven microrobots can be utilized for cargo transportation. A micropump has been devised to trap polystyrene beads in the given medium shown in Figure 24(E){i}, in which four light beams are focused toward the respective four spherical handles to drive the micropump [172]. Also, another light beam is focused toward the micropump top hole to illuminate the thin metal layer that generates heat inside it for producing microbubbles, which further generates strong convection currents into the micropump to pull the polystyrene beads into it, as shown in Figure 24(E){ii}. Figure 24{E}{iii} shows that the moment the particles arrive in the vicinity of these bubbles, they are trapped by surface tension. However, the particles are ejected by forming the bubble disturbance that can be caused by the adjustment of heating beam positions. The TiO_2_ micromotor shown in Figure (24){F}{i–ii} can capture cargo by migration and diffusion. It used H_2_O_2_ aqueous solution as fuel to propel the microrobot under UV irradiation [173]. Figure 24(F){iii} shows micromotors under UV light exposure first approaching cargo 1 for capture, then relocating them to the destination of objective 3, and finally releasing them by turning off the UV light. Also, the micromotor approaches cargo 2, transports it to the location near target 3, and releases it with guided UV light.

### 5.3 Environmental restoration and surface cleaning

**Figure 25:**
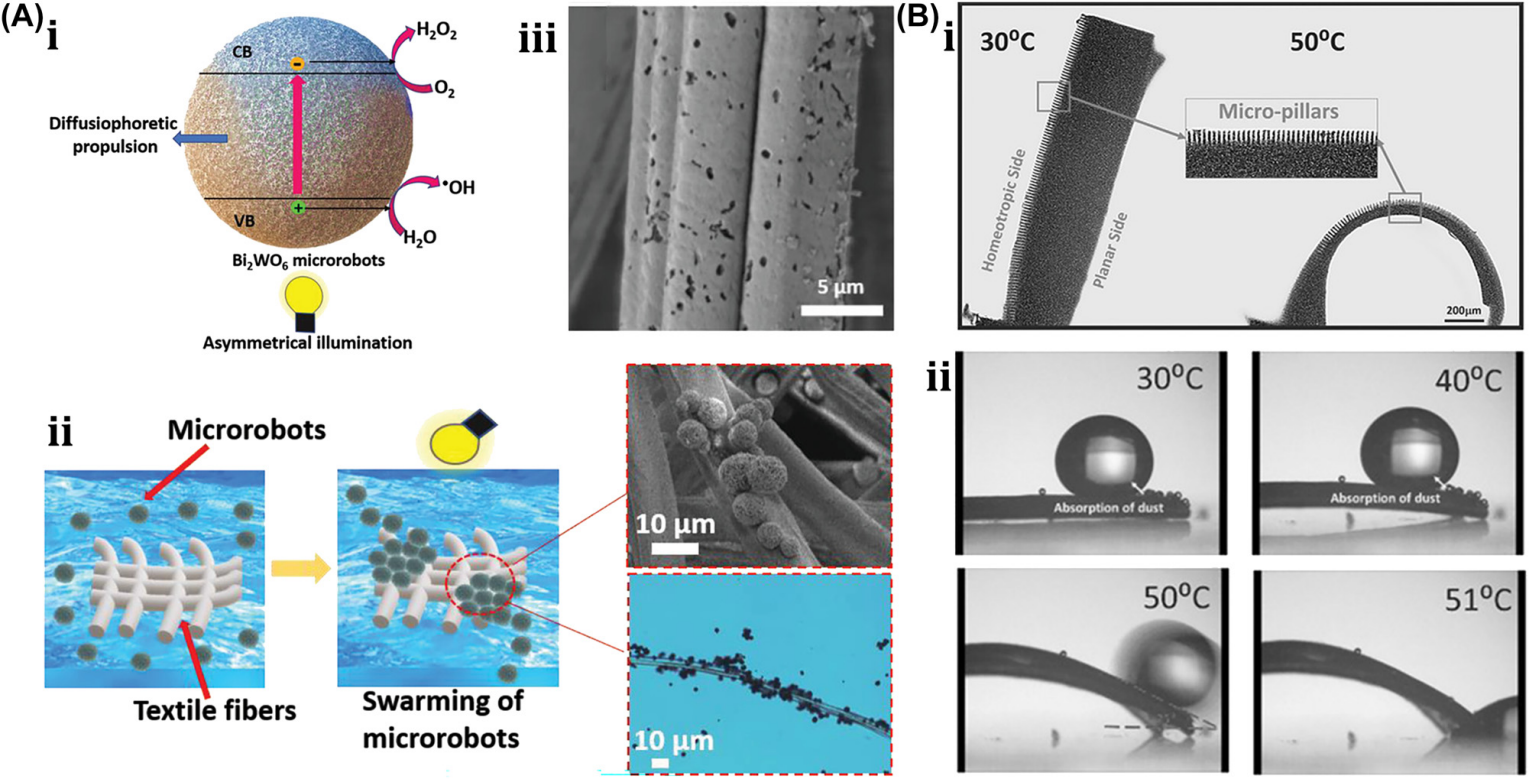
Environmental restoration and surface cleaning. (A) (i) Bi_2_WO_6_ perovskite material microrobot as a textile degradation. (ii) Operation mechanism of textile degradation with microrobots. (iii) SEM image of damaged surface of textile. Reproduced with copyright permission from [174]. (B) (i) LCE film deformation under thermal stimulation. (ii) Self-cleaning with the demonstration of glycerol droplet motion on the micropatterned LCE film due to thermal induced curvature. Reproduced with copyright permission from [12].

In the last few decades, the environmental issue has grown exponentially with the substantial increase in population and industrial development, which causes continuous sewage discharge in ecosystems. This sewage comprises pollutants such as inorganic salts, acids, and alkalis, organic substances, and toxic substances, including colored pollutants, suspended substances, heavy metals, etc. So, the research and scientific community motivates and makes an effort to solve ecological restoration using optically triggered microrobots efficiently. The Bi_2_WO_6_ perovskite material–based microrobot shown in the figure for textile destruction, in which the electron–hole pairs induced on the microrobot surface with the light exposure, react with water and O_2_ to produce charge variation with highly oxidizing compounds, including H_2_O_2_, hydroxyl radicals $\left(\cdot \text{OH}\right)$ , protons $\left(H^{+}\right)$ , and hydroxyl radicals $\left(-OH\right)$ , is depicted in Figure 25(A){i} [174]. These highly oxidizing substances in microrobots are nonuniformly distributed due to their uneven surface, which establishes the concentration gradient around it to drive the microrobot with self-diffusion. Figure 25(A){ii–iii} shows the degradation of fiber, in which microrobots congregate and move themselves attached to the fiber in the presence of light.

A Geckos features–inspired microrobot was developed for surface cleaning. The LCE films have been utilized to simulate the gecko toe muscle motion. An integrated soft lithography technology is used for the micrographic processing of LCE layers, resulting in microcolumns on their surface. Figure 25(B){i} shows the deformation in specific patterned LCE films with applied thermal stimulation [12]. Further, the LCE sample properties are also demonstrated in self-cleaning by putting a small glycerol drop on the micropatterned and unlithographed LCE films. The LCE film bends with the rise in temperature, resulting in microcolumn on the films. The thermally induced curvature corresponds to sliding angles that cause glycerol drops on LCE films to slide down with the shortest path, and Figure 25(B){ii} shows the microparticles attraction to glycerol droplets on the films. So, optically triggered microrobots have distinct features in environmental remediation because they are sensitive to light exposure and capable of self-driving in the solution. Throughout the mixture solution, the reactive reactant distribution and reaction time can be significantly reduced by their motion-induced agitation and self-driven without needing an external mechanical stirrer. These microrobots have high dispersion and small particle sizes, and their inherent features make them widely utilized in microcosmic environment rehabilitation, including microporous plates and microchannels. However, the optically triggered microrobot in environmental remediation is still needed to enhance biocompatibility and efficiency. So, the aforementioned constraints could be addressed through structural design optimization, devising admirable photoactive materials, and eco-friendly fuels.

### 5.4 Bionic technology

**Figure 26:**
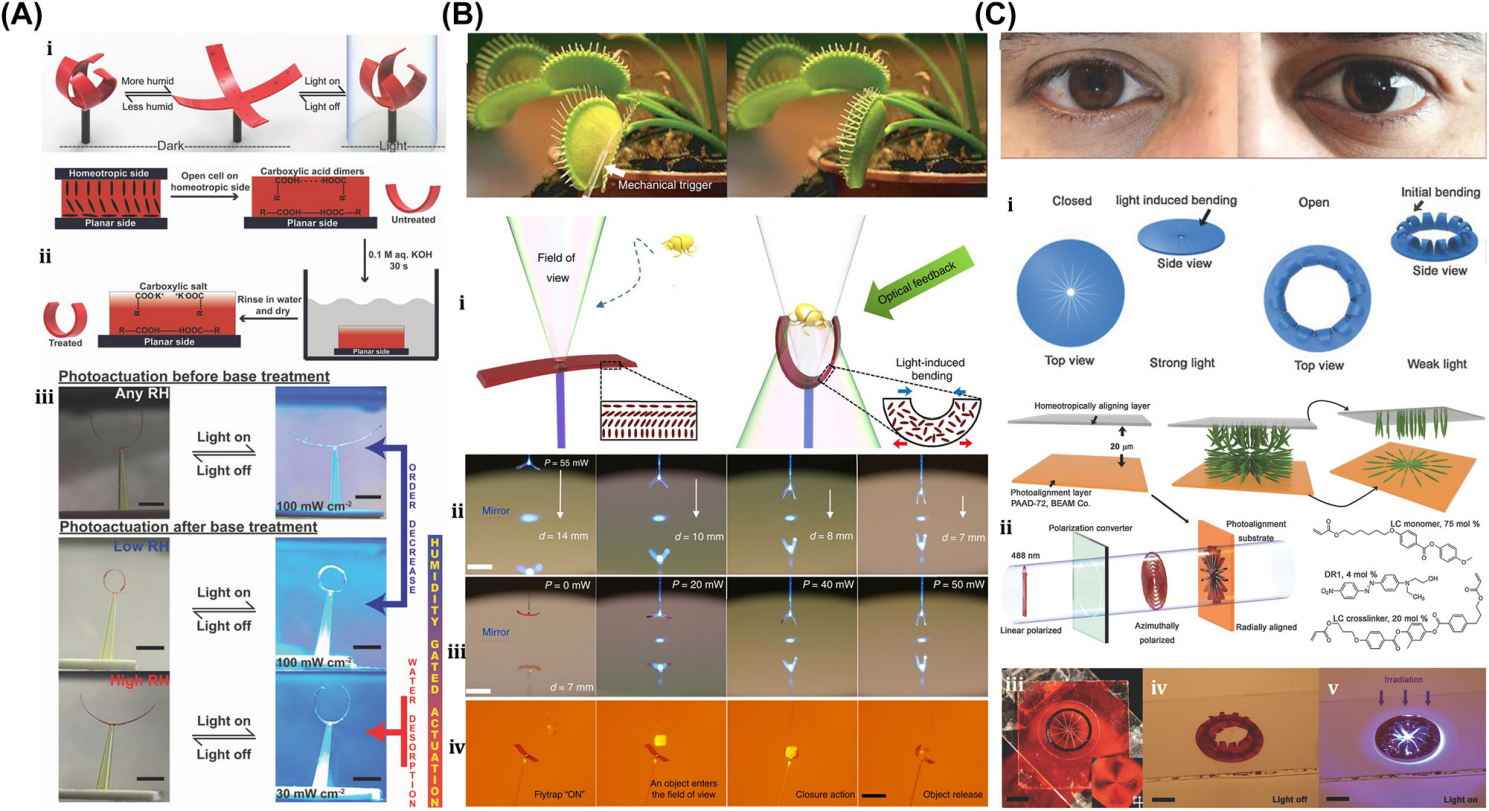
Bionic technology. (A) (i) LCN band–based artificial nocturnal flower. (ii) Alkaline solution treatment of LCN band for the humidity sensitivity. (iii) LCN band actuation before and after based treatment in the presence of light. Reproduced with copyright permission from [13]. (B) (i) Principle mechanism of bioinspired flytrap made by LCE. (ii–iii) Flytrap experimental demonstration. (iv) Object capture and release through flytrap with the feedback of light. Reproduced with copyright permission from [14]. (C) (i) Human eye and conceptual mechanism of artificial iris. (ii) Iris fabrication steps. (iii–v) Iris opening or closing demonstration in the dark or light illumination respectively. Reproduced with copyright permission from [15].

So far, the biology functionality itself is not comparable to any manmade machinery, which motivates the researcher to think about bionic technology that effectively reveals the biology functionality in the engineering field. In 1960, American scientist Steel coined the term “bionics” from the Latin words “bios” (Way of life) and the suffix “nic” (Has the nature of…). Bionic technology is a technology of engineering and science involving unique ideas, functionality, and phenomena to explore the structure and characteristics of biological systems and has the potential to spread a unique technological development route and dramatically extend people’s horizons. With the continuous growth in the development of optical-powered microrobots and bionic materials in recent years, researchers gained the design intuition to mimic biological functions and developed optical-powered bionic microrobots. An artificial nocturnal flower developed with LCP materials has a novel morphing feature called humidity-gated photoactuation that blooms in high humidity or darkness in the evening and withers with low humidity or high light during the day [13]. To ensure the sensitivity of LCP in response to both light and humidity, as shown in Figure 26(A){ii}, it is first immersed in an alkaline solution to render it humidity responsive. However, Figure 26(A){i, iii} shows the influence of humidity on LCP band curvature, where its curvature is large in high humidity but small in low humidity. The humidity-gated photo actuator, unlike conventional photothermal LCP actuators, has the potential to be powered by low light intensities and is capable of remotely functioning through minimal light.

After a year, the light-driven flytrap shown in Figure 26(B){i} was demonstrated, whose photomechanical motor was triggered by utilizing optical feedback [14]. The device is designed with a noncontact probe to sense the environment and is made of a photosensitive liquid crystal elastomer (LCE), which is connected to an optical fiber tip. Figure 26(B){ii–iii} shows a hole left at the center of LCE, through which light coupled by the optical fiber is emitted. Figure 26(B){iv} depicts that the LCP bends to capture an object due to feedback generated by adequate light when the object comes into view. Figure 26(C){i} shows the iris capability, in which the iris can alter the aperture size (Pupil) and accordingly adjust the amount of light entering the eye in response to various lighting circumstances [15]. Also, mechanical drives are needed in order to externally control the artificial iris, which can operate autonomously with the tuning of light intensity instead of any need for a control circuit. It provides a realization of the self-regulation capabilities of the artificial iris.

The bionic iris shown in Figure 26(C){i} is based on LCE and is able to change its shape automatically in accordance with the power density of incident light. The steps involved in the fabrication process of an artificial iris are shown in Figure 26(C){ii}. To begin, a pair of glass slides are arranged together in which their bottom and top surfaces are coated with layers of optical and isotropic alignment, respectively, for a base layer formation. This base layer is then passed through a mixture of liquid crystal monomers. The laser beam of linear polarization is converted into azimuthal polarization using a polarization converter, which is projected on the optical alignment layer-coated substrate so the azobenzene molecules in it adapt to the radial distribution. The behavior of the artificial iris shown in Figure 26(C){iii–v} is similar to that of a natural iris, which opens in the dark and closes with light exposure.

### 5.5 Biomedical application

**Figure 27:**
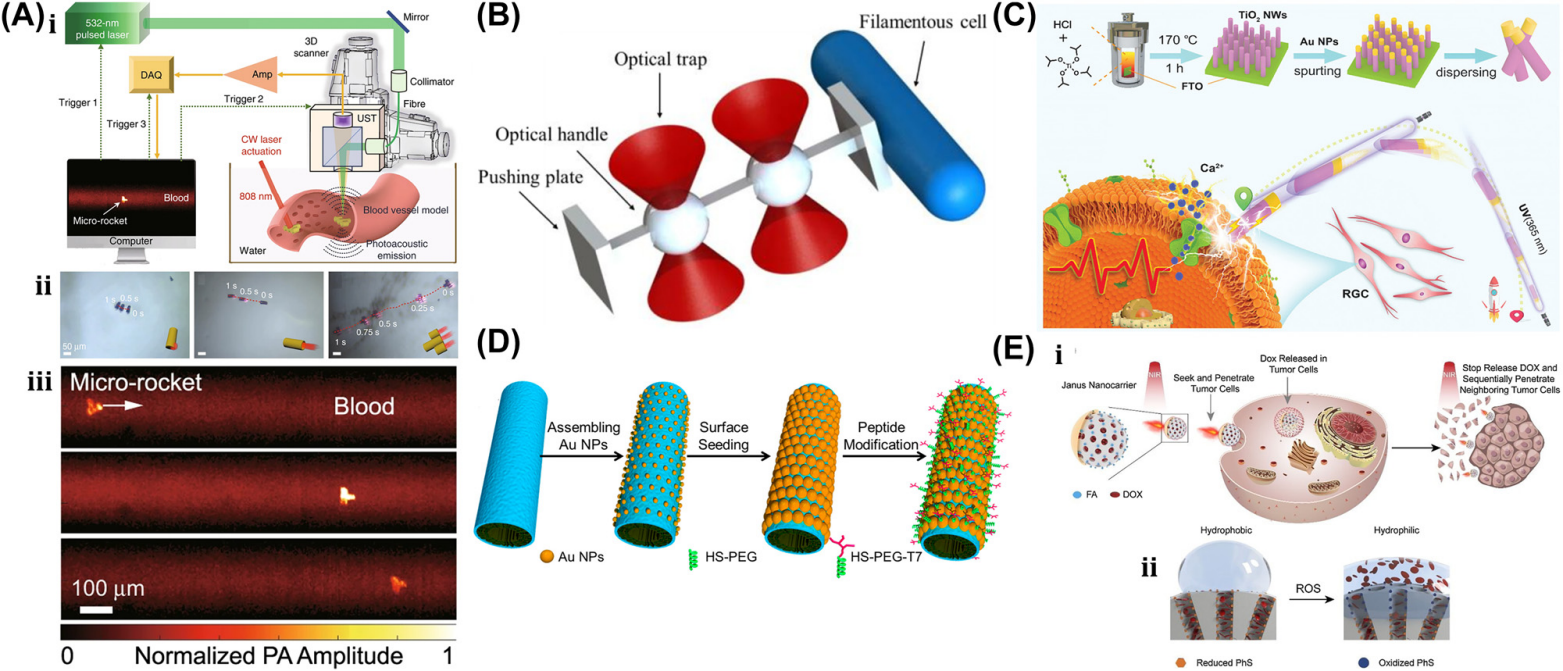
Biomedical applications. (A) (i) Setup of optical resolution photoacoustic microscopy system for tracking of light-driven micro-rocket. (ii) Locomotion of rod type, tube type, and rocket type microrobot in response to light. (iii) Micro-rocket propulsion in blood environment. Reproduced with copyright permission from [175]. (B) OT-driven microrobot for the translation and rotational motion of filamentous cell. Reproduced with copyright permission from [176]. (C) TiO_2_/Au nanowire propulsion for the manipulation of neurons. Reproduced with copyright permission from [130]. (D) Light-driven micromotor for the destruction of cancer cell. Reproduced with copyright permission from [177]. (E) Conceptual model of light-driven Janus micromotor for cancer treatment. Reproduced with copyright permission from [141].

The chemically driven microrobot is the first one that has the potential to propel in blood at high speed, while the toxicity of the chemical fuel of the microrobot is the most significant concern when operating in blood vessels. On the other hand, a magnetically driven microrobot is noninvasive and highly controllable in viscous liquids, but its application is limited in blood vessels due to its low speed and peak power as compared to even the slowest flow capillaries. Such microrobots are precisely injected into the blood vessel through the microinjection device, and then they can be moved in directional motion with the laser to complete the task of cell debris removal and target drug delivery. This process enables improved microrobot motion three times faster than its natural rate. Nevertheless, it encounters problems mainly in self-actuation and visibility. Because once the microrobot enters the bloodstream, it is challenging to track its position in the organs and observe its shape. Therefore, the design of an optical resolution photoacoustic microscopy (OR-PAM) system for tracking optical light–driven microrobots is shown in Figure 27(A){i} [175]. The microrobot is designed in a 3-tube shape like a rocket, as shown in Figure 27(A){ii}, where a short pulse laser light shines on the microrobot surface made of a light-absorbing material and compels the conversion of light energy into sound waves to propel the microrobots. However, the high-resolution tracking of the microrobot shown in Figure 27(A){iii} is enabled by the OR-PAM system, in which a concave acoustic lens collects the optical sound waves generated through laser light and detects them with a piezoelectric ultrasonic transducer. With this research, the microrobot ensures its feasibility in biomedical applications in which microrobot motion in a viscous medium like blood and even their high-resolution tracking using photoacoustic imaging are achievable.

Research on single cells is vital due to the heterogeneity of the cell, especially in biology and medicine. Typically, at the single-cell level, cell heterogeneity is characterized by transferring one or more cells to a desired place and rotating in a specific direction. The optical tweezer-driven microrobots for the translational and rotational motion of filamentous cells are shown in Figure 27(B) because of the growing influence of optical cell manipulation on cell detection [176]. Such techniques can effectively avoid cell damage from direct laser beam irradiation while achieving accurate cell operation. The TiO_2_/Au nanowire-based micromotor in pure water has self-actuation from exposure to extremely low UV light and also has appreciable control over its motion, even in a biological environment [130]. Figure 27(C) shows its capability to reach target neurons of retinal ganglion cells (RGCs) with quite high precision. Then, the calcium channel triggered the target cells in response to electrical stimulus through the generated local electric field due to the water division. Subsequently, calcium channels trigger the target cells, which use the local electric field generated by water division as an electrical stimulus. The study presents new techniques for the accurate and noninvasive delivery of bioelectrical signals and neural activity control. Furthermore, axons are vital to the nervous system’s regeneration and development, and their growth direction depends on a growth cone at the axon tip. The demonstration for the control of axons’ growth direction through the rotation of vaterite particles driven by laser light, where one or two vaterite particles are kept near the growing cone of the neuron axon from the goldfish retina [178]. The polarized light with angular momentum causes the particles to spin, resulting in a local flow of microfluidics that exerts a shear stress on the growing cone, causing it to rotate. The axon growth direction could be accurately controlled with a change in the vaterite particle’s position and rotation direction.

Also, the propulsion of a bubble-driven microrobot made of light-absorbing hydrogel was demonstrated in a phosphate-buffered saline (PBS) solution [179]. The microrobot induced thermal capillaries around its surroundings under exposure to IR laser light, which caused the propulsion. Also, assembling polystyrene microbeads and yeast cells on the slides demonstrated different patterns with the actuation of such microrobots. So, the study demonstrates that the light-driven microrobot has excellent assembly capability, which broadens its application in biomedicine. Smart nanocarriers for drug transportation could possibly reduce the drug’s side effects on the human body. Still, its utilization is limited in many future applications because of its nonuniform distribution and reduced concentration caused by low penetration of tissues and carrier cells. Furthermore, the release of drugs can have an adverse effect on other normal cells. Researchers have manufactured nanocarriers with smaller sizes, modified shapes, or surface groups to increase their penetration into cells and tissues. Although these techniques could enhance only the nanocarrier cell’s penetration, even to a particular extent, their loading efficiency is still limited, and hence, enhancement in nanocarrier cell and tissue penetration remains a significant challenge.

A light-driven microrobot was developed to kill cancer cells made of photothermal materials and decorated with tumor-targeting peptides, as shown in Figure 27(D) [177]. NIR light irradiation induces a photothermal effect, which results in microrobot motion. Also, the microrobot surface has a targeted peptide, where the photothermal effect enables the micromotor to recognize the cancer cells and then kill them without damaging the normal cells. The TiO_2_/Au micromotor was devised by modifying the surface with doxorubicin and fatty acids for a clinical medication that is the most widely used oncology drug. The auto-thermal conductivity forces induced by NIR radiation push the micromotor close to the cancer cell, enabling it to penetrate the cell membrane with thermo-mechanical forces. The control of drug release is shown in Figure 27(E), which is controlled by the surrounding environment-assisted switch opening and closing of the water channel [141]. This technique’s incorporation of switching control, recognition, and penetration makes it excellent for easing drug side effects and enhancing micromotor utilization. To target drug delivery in the intestine, a two-layered micromotor was developed with a magnesium inner layer and a gold outer layer that is covered with the drug and polyether layer [180]. When the micromotor gold layer is exposed to light, it induces the photothermal effect, resulting in the breakage of the polyether layer. This releases the micromotors and stimulates their motion due to the hydrogen bubble produced by the reaction of the magnesium layer with water. The technique demonstrates a significant contribution to target drug delivery and has a possibility in the future prospect of precision medicine.

Furthermore, nontoxic compounds like NO_3_ may be spontaneously metabolized by many bacteria into antitumor products like NO, although such biological reactions are frequently very weak to assure high efficiency and stability. So, to address the aforementioned problem, introduce the technique of nanophotocatalysts in order to charge the bacteria. The process involves a significant number of exogenous electrons for the bacteria to charge, which aids in controlling their metabolic activities in order to enhance their anticancer potential [181]. However, in photocontrolled bacterial metabolite treatment (PMT), *Escherichia coli* (*E. coli*) harbors enzymes for the generation of nitric oxide (NO) coupled through carbon nitride (C_3_N_4_). The C_3_N_4_ generates photoelectrons with light stimulation and transfers them into *E. coli* in order to reduce the endogenous enzymes to NO. The studies of PMT treatment in a mouse model report that tumor growth was inhibited by 80 %. Unlike the needs of other applications, the implementation of light-powered microrobots with biocompatibility is an immense challenge in the biomedical field. Since microrobots need *in vivo* interaction with the human body, the utilization of chemical fuel for the propulsion of microrobots is strictly prohibited. Even UV light is harmful for the human body, so IR light is mostly preferred instead of UV light for microrobot actuation. Research and the scientific community have focused more attention on microrobots, which are developed with consideration of both infrared photosensitive materials and biocompatibility. Recently, light-driven microrobots with thermal effects caused by gold have been disclosed to have vast potential in the application of therapy and *in vivo* diagnosis. Aside from biocompatibility, issues with microrobots like weak driving force, bubble accumulation, and metal ion concentration tolerance still need to be solved.

### 5.6 Biocompatibility insight in light-driven soft bio µn-Bots

Integrating µn-Bots into biomedical applications necessitates an in-depth consideration of their biocompatibility, especially in highly sensitive environments, such as those involving cell cultures or internal tissues. Traditional light-driven microrobots have predominantly relied on synthetic materials or composites, often raising immune response issues, cytotoxicity, or biofouling when interfacing with biological environments. So, biocompatibility remains a central concern in developing and deploying µn-Bots for biomedical applications [182]. Traditional microrobots, often composed of metallic or synthetic components, face considerable limitations *in vivo* due to potential cytotoxicity, immune responses, or mechanical mismatch with soft biological tissues. Recent reviews highlight that biocompatibility is one of the four fundamental pillars alongside propulsion, cooperation, and intelligence that define the future trajectory of µn-Bots innovation. However, smart and biologically responsive materials are crucial to achieving systems that can function harmoniously within physiological environments without inducing damage or dysfunction [183].

Utilizing biological materials or whole biological organisms as µn-Bots presents a compelling approach to address the biocompatibility challenge. Several studies have reported the development of light-driven bio µn-Bots, leveraging the intrinsic properties of living cells such as motility, adaptability, and responsiveness to light stimuli. These systems exhibit minimal immunogenicity and high functional integration with biological systems, making them ideal candidates for biomedical applications.

For instance, *Chlamydomonas reinhardtii*–based optically controlled living micromotors have been developed to achieve advanced biomedical tasks, including manipulation and disruption of biological targets such as blood clots. These systems function effectively in complex biological fluids like serum, plasma, and even bone marrow and are capable of performing operations like indirect manipulation and aggregate disruption. Moreover, they can be assembled into programmable arrays, enabling collective actions for coordinated bio-tasks, further underscoring the utility of biological systems in constructing biocompatible, multifunctional platforms [184]. In a parallel effort, a bio-micromotor tweezers system was introduced using optically trapped rotating *Chlamydomonas reinhardtii* (CR) cells. These microalgae possess intrinsic motility and phototactic behavior, making them suitable microrobotic agents. By harnessing localized hydrodynamic flows generated by rotating CR cells, the system enabled noncontact manipulation and delivery of biological cargos, including cells and drug particles. The platform avoided harmful laser exposure and complex loading/unloading procedures, significantly preserving the bioactivity and viability of cargo. The technique was further validated through targeted drug delivery into single cancer cells, illustrating its potential in precise, cell-specific therapy [185].

A novel class of opto-hydrodynamic diatombots (OHDs) was developed using *Phaeodactylum tricornutum*, actuated via light and hydrodynamic effects for the noninvasive trapping and removal of nano-biothreats. The diatombots achieved high removal efficiency of pathogens such as adenoviruses, mycoplasmas, and bacteria without adversely affecting the viability of co-cultured sensitive cells such as hippocampal neurons. This biohybrid platform not only demonstrated effective biothreat elimination but also enabled gene delivery, emphasizing its multifunctionality and high compatibility with cellular systems [186].

Furthermore, light-controlled actuation of biological microrobots is presented in developing a photonic nanojet-regulated soft microalga robot (saBOT) using *Euglena gracilis*. Using *Euglena gracilis* as the chassis, deformation was induced via localized light focusing on the organism’s photoreceptor, channelrhodopsin-2 (ChR2). It enabled precise control of shape and motion in confined microenvironments. Due to their adaptability and inherent biological compliance, the saBOTs demonstrated navigational capabilities in dense cellular matrices and successfully delivered therapeutic agents to targeted locations [187]. Further innovation led to the “Ebot,” a light-controlled soft microrobot derived from *Euglena gracilis*. The Ebot employs light-regulated polygonal flagellar beating to achieve controllable locomotion and deformation. The system could traverse narrow and curved biological environments such as intestinal mucosa. The Ebot successfully executed biomedical tasks, including targeted drug delivery, selective removal of diseased cells, and photodynamic therapy. The high deformability and biocompatibility of the Ebot present a versatile platform for *in situ* medical interventions in complex and inaccessible tissues [188].

Together, these studies demonstrate the growing maturity and promise of light-driven biological µn-Bots. By exploiting the inherent properties of motile microorganisms and leveraging precise light-based control strategies, these systems offer high spatial and temporal resolution, environmental adaptability, and, most importantly, superior biocompatibility. Their ability to operate safely in diverse physiological media and perform targeted biomedical tasks underscores their potential for future clinical translation and therapeutic deployment.

## 6 Multimodal collaborative control strategy with optical actuation: hybrid actuated soft µn-Bots

**Figure 28:**
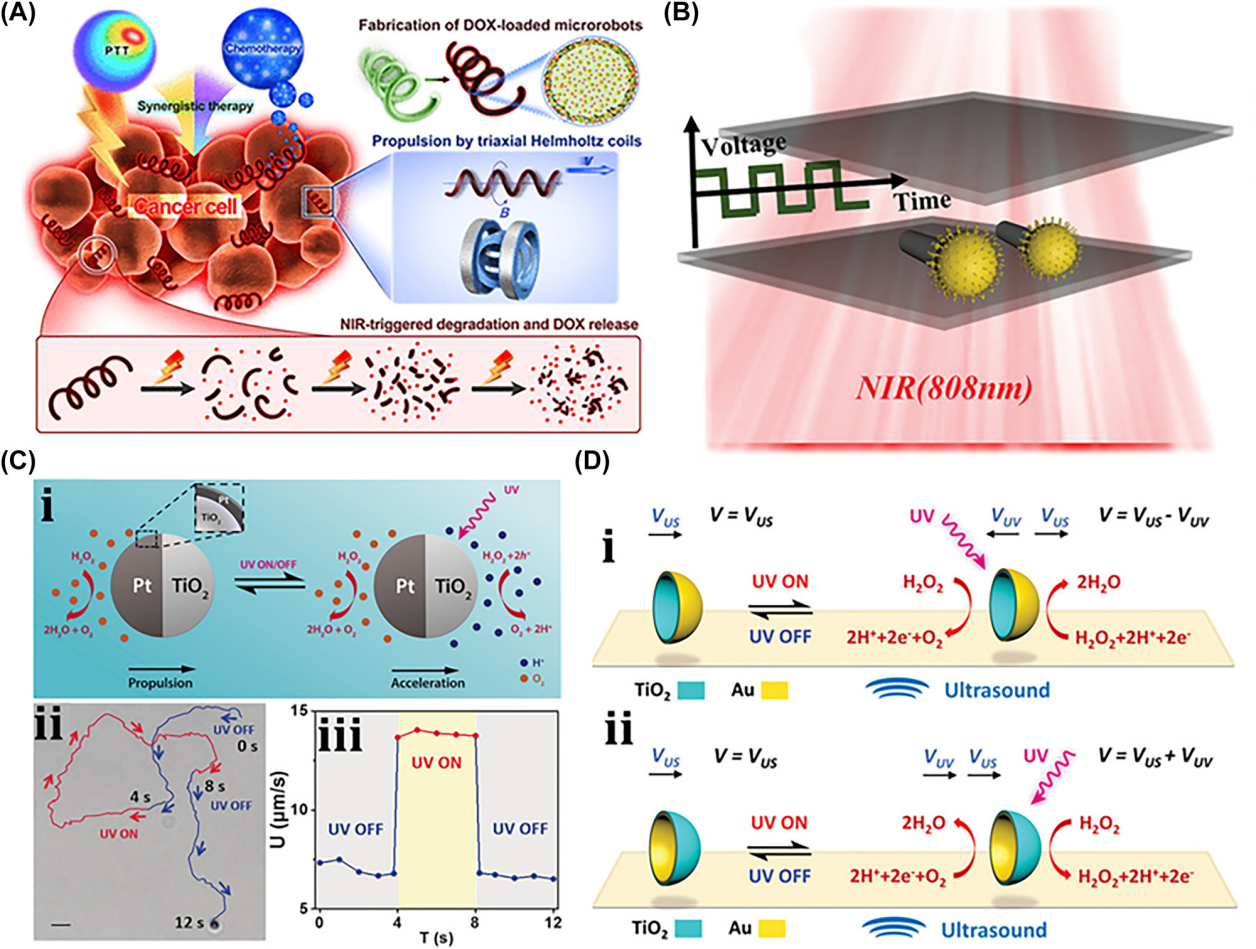
Biomedical applications. (A) Pd@Au/Fe_3_O_4_ template loaded with doxorubicin drug–based µn-Bots under optical–magnetic fields. Reproduced with copyright permission from [189]. (B) Actuation of Gold@TiO_2_ − SiO_2_ Janus nanotree microswimmer under optical–electrical fields. Reproduced with copyright permission from [190]. (C) “on-the-fly” behaviors of TiO_2_/Au/Pt micromotors due to optical–chemical actuation. Reproduced with copyright permission from [191]. (D) TiO_2_ − Au and Au − TiO_2_ configurations microbowl actuation under optical and acoustic fields. Reproduced with copyright permission from [192].

Micro-robotics has made significant strides in developing various actuation methods to navigate µn-Bots through complex environments. Light-based actuation offers advantages such as high spatiotemporal resolution, remote control, and noninvasive functionality. However, its limitations – such as limited penetration depth in biological tissue and restricted propulsion forces – pose challenges in practical applications. Recent studies have emphasized multimodal collaborative control strategies to overcome such limitations, where optical actuation is synergistically integrated with other physical stimuli such as magnetic, electric, chemical, or acoustic fields as depicted in Figure 28, respectively. This hybridization approach not only enhances the controllability of µn-Bots but also introduces new functionalities such as swarming behavior, adaptive shape transformation, and bioimaging-guided therapy. More importantly, the orthogonality of light to other fields allows the division of roles – for example, one mode for propulsion and another for navigation or therapy. Such rational integration ushers in a broadened design space and sophisticated control strategies necessary for real-world biomedical, sensing, and manufacturing applications.

### 6.1 Optical–magnetic actuation strategy

Magnetic and optical actuation integration is the most extensively studied among all hybrid strategies due to its complementary features. Magnetic fields provide deep tissue penetration and stable control, while light brings tunability in energy, wavelength, and polarization for localized interaction and responsive behavior. This dual-mode control enables both global maneuvering and localized activation.

Biohybrid microrobots based on Pd@Au/Fe_3_O_4_ template loaded with doxorubicin drug explored in the application targeted drug delivery and synergistic chemo-photothermal therapy are shown in Figure 28(A) [189]. The resulting microrobots can be propelled through an external rotating magnetic field to facilitate drug delivery and perform synergistic chemo-photothermal therapy to kill EC109 and 769-P cancer cells with the irradiation of 808 nm NIR laser light. Significantly, the biohybrid microrobots can decompose under NIR irradiation and discharge pharmaceuticals into the surrounding environment. It demonstrates a comprehensive approach to the fabrication of multifunctional microrobots that feature rapid swimming speed, accurate directional control, exceptional drug loading efficiency, and effective photothermal conversion aimed at applications in targeted drug delivery and combined chemo-photothermal therapy within a singular microrobotic platform.

Similarly, a photosynthetic biohybrid nanoswimmer (PBN) system based on *Spirulina platensis*, a magnetically modified bacterium for the target imaging and treatment of the tumor [193], was synthesized with the utilization of superparamagnetic magnetite (Fe_2_O_4_ NPs) by a dip-coating technique to facilitate tumor targeting and magnetic resonance imaging capabilities after intravenous injection. Here, PBNs can function as oxygenators for *in situ* O_2_ production in hypoxic solid tumors with photosynthesis, which changes the tumor microenvironment (TME) to enhance the efficacy of radiotherapy (RT). Also, the inherent chlorophyll emitted from the RT-treated PBNs, functioning as a photosensitizer, can generate lethal reactive oxygen species upon laser irradiation to facilitate photodynamic therapy. This research offers an innovative framework for attaining tumor-specific augmented combination therapy guided by fluorescence, photoacoustic, and magnetic resonance imaging.

A multi-responsive soft material–based microrobot was fabricated through a magnetic-field–assisted gradient assembly of Fe_2_O_4_ NPs and GO that can be actuated through both magnetic field and light [194]. However, the microrobot fabricated with Fe_2_O_4_ NPs@GO composite film was sensitive to various stimuli such as magnetic field, light, moisture, and sound, which allowed it to execute complex motions, including translation, rotation, bending, grabbing, and releasing. Similar to above, Figure 24(A) in Section 5.2 shows a remote controlled soft microgripper that shows proficiency in loading, conveying, turning, and releasing cargo, which have already been discussed.

### 6.2 Optical–electric actuation strategy

The combination of optical and electrical fields presents a powerful microrobot propulsion and control strategy. This multimodal actuation exploits optical stimuli’s high tunability and spatial resolution with the penetrative and stable manipulation characteristics of electric fields, enabling enhanced maneuverability, directional control, and functionality in microscale systems. Several recent advances demonstrate the effective synergy between these two fields in enabling self-electrophoresis, induced-charge electrophoresis, electrorotation, and optically programmable alignment.

A light-programmable micro/nanomotors composed of silicon nanowires suspended in aqueous solution and actuated in external electric fields were demonstrated [195]. Their motion is governed by optically tunable in-phase electric polarization, which modulates alignment direction and rate within an AC field under visible light exposure. By selectively illuminating the nanowires, their real-part electrical polarizability is modified, resulting in instantaneous alignment switching between parallel and perpendicular configurations with respect to the electric field. This light-tunable behavior enables on-demand switching and encoding of mechanical signals, as demonstrated by nanowires transmitting Morse code via controlled reorientations. This mechanism offers a highly responsive and reconfigurable approach to controlling microrobot orientation, with potential applications in signal communication and collaborative behaviors. Also, the multifold reconfiguration of Si nanowires rotation in electric field was assisted by visible light stimulation [196]. The orientation and rotation speed of silicon nanowires were modulated by varying light intensity from visible to IR regime and AC electric field. This resulted in multiple rotational regimes, including acceleration, deceleration, reversal, and even complete stop, depending on the interplay between light intensity and AC frequency.

Optoelectronic rotor mechanisms have also been realized with the halide perovskites (HP) CsPb(Br/I)_3_ nanorods [197]. HP nanorods undergo polarization under a rotating electric field, and subsequent light illumination generates charge carriers that amplify the rotational torque, surpassing Brownian motion to initiate controlled rotation. Also, it exhibits reversible switching between “ON” and “OFF” states through light modulation, with its rotational speed precisely tunable via the intensity of incident light, highlighting a promising functional application in micro/nanobots. In addition to above, a gold-loaded titania-silica (Gold@TiO_2_ − SiO_2_)-based Janus nanotree microswimmer as shown in Figure 28(B) exhibits multimode self-propulsion in the presence of light, heat, and electric field includes optochemical self-electrophoresis with UV and visible light, self-thermophoresis with NIR light, and induced-charge electrophoresis under AC electric field, respectively [190]. The direction and velocity of migration can be tuned by light intensity and surface charge modification, demonstrating the potential of photoelectrochemical hybrid propulsion in micro/nanobots.

These studies highlight the promise of optical–electrical hybrid actuation, which leverages both light-induced photophysical effects and electric field interactions for precise, remote, and reconfigurable microrobot control.

### 6.3 Optical–chemical actuation strategy

Integrating optical and chemical propulsion mechanisms represents a transformative approach in microrobot design, enabling enhanced functionality, adaptability, and precision in complex environments. By combining light-driven actuation with chemical fuels, these hybrid systems leverage the advantages of both modalities: optical control offers spatiotemporal precision and remote operation, while chemical propulsion provides high energy density and sustained motion. This synergy addresses limitations inherent to single-mode systems, such as fuel dependency or limited directional control, and unlocks new possibilities in environmental remediation, targeted therapy, and microscale manipulation.

A hybrid optical–chemical powered Janus hybrid micromotor designed by incorporating photocatalytic TiO_2_ and catalytic Pt surfaces, separated by an intermediate Au layer as shown Figure 28(C) [191]. The propulsion of TiO_2_/Au/Pt micromotors is governed by the competing catalytic decomposition of hydrogen peroxide (H_2_O_2_) on the Pt side and the UV-triggered photoelectrochemical reactions on the TiO_2_ side. Since photocatalytic activity on the TiO_2_ surface can mitigate the propulsion force produced by the Pt catalyst, that enables the fine regulation of speed, direction, and optical braking through modulation of light intensity or fuel concentration. An intermediary Au layer is essential for modulating the optical braking effect. Without the Au layer, light exposure induces motion acceleration due to the combined catalytic and photocatalytic activity effects. Light stimuli facilitate many dynamic behaviors, including slowing, braking, reversing, and acceleration, offering more “on-the-fly” behaviors to improve real-time motion control capabilities.

Another optical–chemical driven Janus nanomotor fabricated by embedding Pt and CuS nanoparticles on dendritic porous silica nanoparticles (DPSNs) as carriers via a Pickering emulsion and electrostatic self-assembly method exhibiting dual-propulsion with H_2_O_2_ and NIR light [198]. These Janus nanomotor’s motion utilizes chemical propulsion–induced self-diffusiophoresis due to Pt-catalyzed decomposition of H_2_O_2_ and optical propulsion owing by self-thermophoresis caused by the photothermal effect of CuS NP under NIR light. Their speed can be precisely modulated by adjusting the H_2_O_2_ concentration and NIR light intensity. Overall, optical–chemical hybrid micromotors leverage the reactivity of chemical fuels with the precision of light-based control. The interplay between catalytic surfaces and photo-responsive components enables switchable and programmable propulsion modes, offering advanced strategies for autonomous operation, targeted delivery, and on-demand decontamination.

Also, a water-driven TiO_2_/Au/Mg spherical micromotors developed that have bubble propulsion and photocatalytic degradation of chemical and biological warfare agents [199]. The magnesium core reacts with environmental water, producing hydrogen bubbles that autonomously propel the micromotors without external fuel. Simultaneously, UV-activated TiO_2_ surfaces generate reactive oxygen species (ROS), enabling efficient photocatalytic oxidation. This dual-functionality was applied to degrade nerve agents like methyl paraoxon and bis(4-nitrophenyl) phosphate and inactivate *Bacillus globigii* spores. Self-propulsion enhances fluid mixing and mass transport, accelerating decontamination. Control experiments confirmed that motion and light activation are critical for achieving high degradation efficiency, illustrating the synergistic effect of optical–chemical integration. A PMMA − Ag based Janus colloidal motor was developed that exhibited tunable pulsating motion by integrated optical–chemical actuation in H_2_O_2_ and KCl solutions under UV illumination [200]. The propulsion arises from a redox cycle between Ag and AgCl, generating periodic ionic gradients that drive motion through self-diffusiophoresis. The pulsation frequency and amplitude are modulated by light intensity and chemical concentrations. A final propulsion burst occurs upon light-off, attributed to Ag-catalyzed H_2_O_2_ decomposition. It also demonstrates oscillatory interactions with tracer particles and negative gravitaxis. This model establishes a controllable, light-responsive chemical oscillator with potential in active matter and microscale robotic systems.

### 6.4 Optical–acoustic actuation strategy

Acoustic fields offer long-range, high-penetration manipulation, instrumental in *in vivo* environments. The synergistic use of light and acoustic fields enables manipulation strategies that mimic biological collective behavior for swarm aggregation or dispersal, bioimaging, and mechanical interactions like cell membrane penetration. Also, it offers functional enhancements like ultrasound-guided levitation with light-directed activation.

A hybrid light-acoustic powered micro bowl motor made with TiO_2_ − Au and Au − TiO_2_ configurations that have been designed to be propelled by an acoustic field toward its bowl’s exterior surface as shown in Figure 28(D) (i–ii) [192]. However, UV light triggers self-phoretic propulsion of the TiO_2_ surface of the microbowl motor in the H_2_O_2_ medium, allowing for acceleration, braking, or reversal motion depending on whether the TiO_2_ surface is configured on the microbowl’s exterior or interior. Furthermore, the modulation of UV light can control structure-dependent collective swarm behavior, including expansion or compaction, which mimics biological systems’ aggregation or separation behavior. Such structure-dependent control of motion enables a wide range of sophisticated microscale tasks, including cargo transportation and targeted drug delivery, with potential applications in both biomedical and environmental fields.

Furthermore, light-acoustic nanomotors have been demonstrated to mimic biological systems aggregation/separation behavior, in which aggregation is induced through an acoustic field and achieves “firework” separation triggered by an external light signal, resulting from the modulation of acoustic streaming by optical force [201]. Moreover, the configuration of the “firework” can be altered by adjusting the irradiation position and angle. This method applies to metallic, metalloid, and polymeric materials, offering potential for chemical sensing, cargo transport, and biological applications.

In addition to manipulating mobility and collective behavior, NIR light can also facilitate photomechanical penetration of cancer cell membranes when combined with ultrasound-driven polymer nanoswimmers, which are made by gold-nano shell functionalized layer-by-layer deposition tubular structures [202]. A polymer nanoswimmer was constructed using layer-by-layer deposition and functionalized with gold nanoshells. This tubular nanoswimmer method utilizes an acoustic field to target cells. The acoustic driving force is significantly less than the critical force of the cell membrane; hence, the nanoswimmer requires supplementary photomechanical force from NIR light to effectively breach the cell membrane. Controllable nanoswimmers capable of perforating cell membranes may facilitate advancements in fast drug delivery, subcellular surgery, and therapeutic interventions. Also, a detection platform that integrates the acoustic aggregation of modified Au nanorods, which produces an increased Raman signal, facilitating ultra-trace fast biomolecule detection in a 1-μL solution [203].

It is inferred that incorporating an additional light/ultrasound signal may result in atypical functionalities, including investigating auxiliary motion mechanisms in µn-Bots and active sensing. Due to their greater density compared to the medium, artificial µn-Bots typically migrate toward the border as a result of natural sedimentation. The charged border will substantially influence the velocity and trajectory of µn-Bots through hydrodynamic, electrostatic, or phoretic mechanisms [192], [201], [202], [203]. The impact of the substrate remains a persistent enigma; nevertheless, ultrasound manipulation presents a viable method for levitating µn-Bots, thereby mitigating substrate effects and elucidating the underlying mechanism. A photoactive dielectric-AgCl Janus motor operated by ultrasound, engineered to capture particles on a pressure nodal plane using megahertz acoustic standing waves. The border exerts a negligible influence on the velocity or directional behavior of PMMA − AgCl Janus micromotors [204].

Despite their promise, multimodal microrobots face significant engineering and application challenges. Integrating multiple actuation systems into a microscale platform often increases complexity, material incompatibility, and reduced efficiency. Synchronizing responses across different stimuli with unique spatial and temporal characteristics requires precise calibration and feedback systems. It emphasizes the need for advanced material synthesis and design strategies. For example, hierarchical structures that compartmentalize stimuli-responsive domains or nanocomposites that exhibit multifunctional responsiveness can support compact integration. Furthermore, real-time imaging and signal feedback mechanisms, especially optical or acoustic modalities, are essential to close the control loop in future autonomous µn-Bots systems. Multimodal µn-Bots are poised to revolutionize applications such as minimally invasive surgery, targeted therapy, biosensing, environmental remediation, and micro-manufacturing. Research should focus on scalability, biocompatibility, energy-efficient powering (e.g., wireless recharging via light or electromagnetic fields), and robustness in heterogeneous environments like blood, tissue, or industrial fluids. The future vision includes programmable microrobotic swarms that communicate via encoded light signals, adapt to environmental stimuli, and perform collective intelligent behaviors. The development of modular platforms, where different actuation modules can be interchanged or activated on demand, may offer practical routes to reconfigurable microrobots tailored to specific tasks.

## 7 Conclusions

In the recent past, light-driven µn-Bots have garnered substantial attention and they have been integrated into a diverse range of applications such as manipulators, space exploration, cell positioning, and drug delivery. These µn-Bots have touch-free control, propulsion, energy efficiency, intelligence, navigation, and tracking, which have prominent potential in medical, defense, and industrial fields. Within the scope of present study, we delve into the µn-Bots and explore actuation mechanisms, designs, functionalities, and material perspectives, then more specific categories of light-powered µn-Bots. Lastly, we showcased the advances in state-of-the-art optical and nonoptical actuated µn-Bots in their broad applications in sectors encompassing biomedical, terrestrial, and aerial µn-Bots, environmental conservation, and transportation research. These applications serve as catalysts, motivating us to explore the unexplored potential of light-powered µn-Bots in various multidisciplinary domains. The latest trends and state of the art in nonoptically driven soft µn-Bots comprised of diverse actuation mechanisms. Magnetic field–driven µn-Bots can be controlled by an external magnetic field, which allows for precise movement without physical contact, making them suitable for medical applications like drug delivery, target therapy, cargo transport, and environmental remediation. However, challenges such as magnetization, film coating, and fabrication methods remain. Likewise, examples of electrically controlled µn-Bots include microgripper, microsurgery, and microassembly, where orientation, position, and speed can be achieved by either the applied electric field or remote-control techniques such as electrorotation, electro-percolation, and electrophoretic motion. However, they require a special buffer and have compatibility issues with bio samples in highly ionic media. The propulsion in thermally actuated µn-Bots is mainly due to joule heating, infrared radiation, or laser heating and can produce complex motion patterns but requires thermal management, especially in biomedical applications. Compared to others, chemical reactions are advantageous for µn-Bots propulsion in fluid environments like the human body, as they generate motion without external forces or power sources. Also, such µn-Bots include microtubular jet microrobots, catalytic micro/nanomotors, and electro-osmotic microswimmers, which have been demonstrated in applications like medication administration, environmental tracking, microfluidics, regulating fluid movement, and real-time environmental observation. Another biocompatible µn-Bots, acoustic field–propelled µn-Bots, are much more compatible with biological environments due to their on-demand and noninvasive motions.

As compared to all actuation, optical actuation in µn-Bots has gained popularity because it has a further subclass of actuation, including an optical tweezer, optochemical reaction, photothermal, and photomechanical that depends not only on material properties but also on the interest of a specific application. For instance, optochemically driven µn-Bots are famous in water purification, drug delivery, environmental protection, and biomedical industries, but optomechanical and optothermal mechanisms powered µn-Bots for the pick-up and place of objects, mimic flowers and act as fly traps. With this, we summarize the advantages and disadvantages of various actuation mechanisms for µn-Bots in Table 3. Subsequently, various types of materials used in the design of µn-Bots have been studied and already summarized in Table 1 in Section 2. Also, we had also outlined in Table 2 the feasible material in the design of the µn-Bots.

**Table 3:** Advantages and disadvantages of µn-Bots actuation mechanism.

Actuation mechanism	Advantages	Disadvantages
Magnetic actuations	Noncontact operation	Complex equipment for operation
	Fast response time	High power required for operation.
	High precision	Magnetic materials may be expensive.
	High force output	Magnetic fields interference with nearby electronics
	Good biocompatibility	
	High penetration depth	
Electrical actuations	Low power consumption	Limited locomotion range
	Ease of control	Limited force output
	Excellent compatibility with other than biomaterials	Biocompatibility issue
	Contact free.	
	Inexpensive	
Thermal actuations	Low power consumption	Limited force output
	Highly responsive	Thermal management required
	Compatibility with many materials	
Chemical actuations	Highly responsive	Limited force output
	Energy efficient	Difficult to control direction and speed.
	Autonomous motion	Necessity of surrounding chemical environment.
	Dynamic *in situ* chemical processes	Limited actuation lifetimes
	Good for biological systems	
	Low power consumption	
Acoustic actuations	Noninvasive	Limited force output
	Noncontact actuation	Specialized equipment for operation
	High penetration depth	Difficult to control precisely.
	Biocompatibility in the MHz range	Affected by the environment
	Excellent degree of tunability	
Optical actuations	Noncontact actuation	Limited light spot size
	Higher energy efficiency	Limited motion range
	Optical materials are much softer and lower density similar to biological tissues.	Hazardous in case of UV light
	More facile	Limited penetration
	Scalable fabrication processes	
	Adjustable radiation energy	
	Highly spatially selective	
	Biocompatible and biodegradable	

It was also observed that the µn-Bots driven with optochemical actuation include self-diffusion, self-electrophoresis, self-thermophoresis, and bubble jet techniques, all of which are carried out primarily in liquid environments in the presence of light. However, they predominantly suffer from corrosion that affects their aging or life cycle. Also, the optical actuation depends on the µn-Bots body, whether it is rigid or soft. The optical tweezer techniques are prominently utilized for the movement of rigid µn-Bots through a focused laser, under which momentum transfer, OET and photothermal-induced techniques also play a role in their movement. Moreover, the soft µn-Bots mostly rely upon either photothermal or photomechanical actuation where the material characteristics play a major role. LCP, hydrogel, and photo switch doped LCP are a few of them utilized in such actions. It has been inferred that all the state-of-the-art with various actuation mechanisms presented in Section 3 and Section 5 are inspired by living organisms of nature that have benefits including small size, lightweight, low power, life cycle, and biocompatibility. Also, we summarize the comparative various light-driven µn-Bots actuation schemes perspective of material type, light regimes, propulsion medium, hybrid actuation compatibility, swarm, and biocompatibility in Table 4.

**Table 4:** Summary of various light-driven µn-Bots actuation schemes.

Optical actuation schemes		Material type	Light regimes	Propulsion medium	Hybrid actuation compatibility	Swarm behavior	Biocompatibility
Optical tweezer		Rigid/soft	–	Liquid	–	Yes	Limited
OET		Rigid/soft	–	Liquid	–	Yes	Limited
Momentum transfer		Rigid/soft	NIR	Liquid	–	Yes	Limited
Optochemical	Diffusion	Rigid/soft	UV	Liquid	Magnetic, electrical, chemical, acoustic	Yes	Limited
	Electrophoresis	Rigid/soft	UV, NIR	Liquid		Yes	Limited
	Bubble-jet	Rigid/soft	UV	Liquid		Yes	Limited
	Thermophoresis	Rigid/soft	UV, NIR	Liquid		Yes	Limited
Photothermal		Soft	UV, visible, NIR	Liquid, air	Magnetic	No	–
Photomehanical		Soft	UV, visible, NIR	Liquid, air	Magnetic	No	–

**Figure 29:**
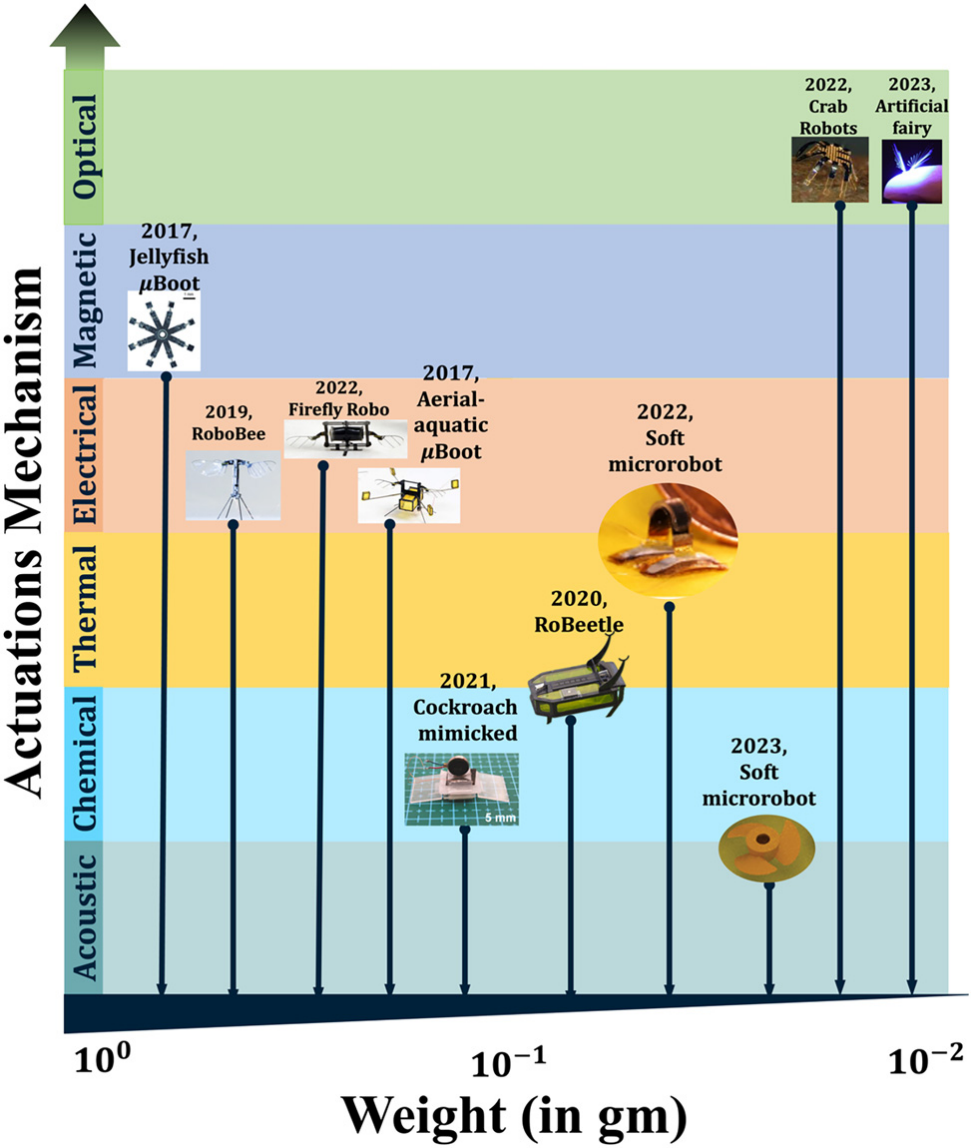
Actuation mechanism versus weight trade off in µn-Bots.

A lot of challenges are still present because of their application-specific operation that will be in prone to areas like defense, biomedical, and many more. For example, the µn-Bots in biomedical application necessitate biocompatibility and safety hazard, while defense requires secure and secret surveillance that requires a small size, is lightweight, and needs a remote control. A beautiful design trend is presented in Figure 29 depicting the actuation mechanism versus weight of μn-Bots evolved in the course of time.

It diagrammatically projects that a light-driven microrobot is much more prominent compared to other actuation mechanisms in terms of size, weight, remote controllability, biocompatibility, etc. At the meantime, the light-driven approaches have also got a lot of capabilities to address the design issue of microrobots, even compatible with some other actuation mechanisms leading into the possibility of more functional hybrid actuation discussed in the Section 6. Such hybrid actuation in µn-Bots is the future direction that may become beneficial as well as more practical to address the current challenges involved in many applications.
